# GWAS reveals genomic associations with swine inflammation and necrosis syndrome

**DOI:** 10.1007/s00335-023-10011-6

**Published:** 2023-08-01

**Authors:** Katharina Gerhards, Sabrina Becker, Josef Kuehling, Mirjam Lechner, Jochen Bathke, Hermann Willems, Gerald Reiner

**Affiliations:** 1 Department of Veterinary Clinical Sciences, Clinic for Swine, Justus Liebig University Giessen, Frankfurter Strasse 112, 35392 Giessen, Germany; 2 UEG Hohenlohe, Am Wasen 20, 91567 Herrieden, Germany; 3 Institute of Animal Breeding and Genetics, Justus Liebig University Giessen, Ludwigstraße 21, 35390 Giessen, Germany

## Abstract

The recently identified swine inflammation and necrosis syndrome (SINS) occurs in high prevalence from newborn piglets to fattening pigs and resembles an important concern for animal welfare. The primary endogenous syndrome affects the tail, ears, teats, coronary bands, claws and heels. The basis of clinical inflammation and necrosis has been substantiated by histopathology, metabolomic and liver transcriptomic. Considerable variation in SINS scores is evident in offspring of different boars under the same husbandry conditions. The high complexity of metabolic alterations and the influence of the boar led to the hypothesis of a polygenic architecture of SINS. This should be investigated by a genome-wide association study. For this purpose, 27 sows were simultaneously inseminated with mixed semen from two extreme boars. The mixed semen always contained ejaculate from a Pietrain boar classified as extremely SINS susceptible and additionally either the ejaculate from a Pietrain boar classified as SINS stable or from a Duroc boar classified as SINS stable. The 234 piglets were phenotyped on day 3 of life, sampled and genetically assigned to the respective boar. The piglets showed the expected genetic differentiation with respect to SINS susceptibility. The suspected genetic complexity was confirmed both in the number and genome-wide distribution of 221 significantly associated SNPs, and led to 49 candidate genes. As the SNPs were almost exclusively located in noncoding regions, functional nucleotides have not yet been identified. The results suggest that the susceptibility of piglets to SINS depends not only on environmental conditions but also on genomic variation.

**Supplementary Information:**

The online version contains supplementary material available at 10.1007/s00335-023-10011-6.

## Background

Swine inflammation and necrosis syndrome (SINS) is a newly identified, specific syndrome, resulting from the combined presence and signs of inflammation and necroses in acral body parts. It particularly affects the tail base, tail tip, ears, coronary bands, heels, soles, claw walls, teats, navel, and face and can be observed in suckling piglets, weaners, and finishing pigs (Reiner and Lechner 2019; Reiner et al. 2019, 2020, 2021a; Kühling et al. 2021a, b). Signs of inflammation and the loss of the integrity of body parts indicate serious impairment of animal welfare and reflect one of the major challenges in pig farming (EFSA 2012, 2014).

Three main observations support the assumption that SINS is primarily an endogenous disease, even though it may be modified by technopathies and other mechanical stressors: (1) The simultaneous occurrence in such disparate body parts as the tail, teats and claws (Reiner et al. 2019; Kühling et al. 2021a, b); (2) Evidence that SINS can be expressed before birth (Kühling et al. 2021a); (3) Evidence that inflammation originating from blood vessels was proven by histopathology before birth when biting and mechanical irritation (e.g., from soil) are excluded, in piglets with (still) intact epidermis (Reiner et al. 2020; Kühling et al. 2021a).

The histopathological background of clinical inflammation is vasculitis, thrombosis, intimal proliferation, oedema, and hyperaemia accompanied by an intact epidermis (Reiner et al. 2020; Kühling et al. 2021a). Inflammation was characterized by granulocytes in considerable numbers, macrophages, and lymphocytes in piglets not older than 2 h, indicating an onset of inflammation at least 4 days before birth (Betz 1994). Bristle loss was associated with inflammatory processes in the deeper parts of the hair follicles (Reiner et al. 2020; Kühling et al. 2021a). Significant proportions of neonates can be affected. In the study by Kühling et al. (2021a) on a conventional farm, 40 to 80% of neonatal piglets were affected by haemorrhages of the claw wall, coronal inflammation, redness of heels, bristle loss, and redness of the tail and ears.

The syndrome is also accompanied by a huge series of clinical-chemical, metabolic (Löwenstein et al. 2021) and transcriptomic (Ringseis et al. 2021) alterations. Practical experience from pig farms with uniform sow base regularly shows evidence of boar effects on progeny SINS scores.

Providing insight into the genetic architecture of SINS would be an important milestone in combating the syndrome, as husbandry improvement measures, often insufficient on their own, could be supported by targeted selection using less sensitive boars. It would make control more effective and sustainable.

This background was confirmed by a study with 19 boars (4 Duroc and 15 Pietrain boars) mated to 39 sows (Kühling et al. 2021b), where the offspring of the boars to be tested were born in the same litter. Offspring from Duroc boars had significantly lower SINS scores (4.87 ± 0.44) than offspring from Pietrain boars (10.13 ± 0.12). Even within the Pietrain breed, SINS scores of offspring were significantly affected by the boar. Total SINS scores in the offspring of the best Pietrain boars was almost 40% lower than that of offspring in the poorest Pietrain boars. These findings confirmed considerable genetic effects on the outcome of SINS under a given husbandry. The genetic background of SINS has recently been confirmed with a heritability of 0.2 (Leite et al. 2023), together with further interesting population parameters.

The present study was conducted to characterize the genetic background of these effects in a genome-wide association study (GWAS) approach. To examine whether evidence of associations with SINS phenotypes can be detected in the porcine genome. In addition, their distribution and magnitude should be evaluated, and candidate genes should be identified, particularly in the areas of inflammation, vasculitis, and necrosis.

## Material and methods

### Study design

The animal experiment was carried out in the conventional pig breeding stable of the Oberer Hardthof teaching and research station at Justus-Liebig University Giessen under the approval of the authorities in Giessen, Germany with file number 708_M.

The sow herd used (Topigs x German landrace) was a rotational cross of Topigs with German Landrace. The boars came from completely different breeds and had no breeding relation between them. The environment was the same for all animals.

The herd had a performance of 15 live born and 1.4 dead born piglets per sow. The sows were artificially inseminated with three extreme boars based on the above mentioned study by Kuehling et al. (2021b). Boars were used in pairs as mixed semen, where the semen of two boars was mixed within one dose. This means that piglets from two different boars were present in each litter at the same time. The design was applied to (i) limit the number of litters and experimental animals, (ii) to minimise environmental effects, (iii) to increase genetic variability within the piglets, (iv) to increase the sow-boar combinations and (v) to nevertheless achieve a manageable number of piglets.

The three boars were a Duroc boar whose progeny had the lowest levels of SINS in the preliminary study (4.3) and the two Pietrain boars whose progeny had the lowest (7.26) and highest (12.17) levels of inflammation and necrosis, respectively, within the Pietrain cohort. These boars were selected to achieve segregation of favourable and unfavourable gene variants in the progeny. All sows were inseminated only once, with mixed semen from two boars, so that piglets of the Pietrain boar classified as unfavourable (PI−) occurred in one litter together with piglets of the Duroc boar (DU) (in 13 litters) or together with piglets of the Pietrain boar classified as favourable (PI +) (in 14 litters). Taken together, a total of 27 sows produced 27 litters. Each sow had only one litter, but with piglets from two different boars. 234 piglets were used, if they were anatomically normal developed and their SINS phenotype was recorded at their 3rd day of life. The piglets’ father was detected by paternity testing after phenotyping. The results of paternity testing revealed 14 mixed litters (from 14 sows) with 77 piglets from the favourable Pietrain boar and 39 piglets from the unfavourable Pietrain boar, as well as 13 mixed litters from 13 sows with 48 piglets from the Duroc boar and 70 piglets from the unfavourable Pietrain boar. On average, 8.4 healthy piglets per litter with at least one piglet per inseminated boar were randomly selected in a blinded manner and used.

### Paternity testing

Genetic matches between offspring and boars were used in paternity testing. DNA was extracted from the piglet’s docked tails. Tail docking was done at day 4 of life, one day after clinical scoring (Reiner et al. 2021b). Genotyping was done with 14 microsatellites in 2 multiplex PCRs and microsatellite alleles were determined by capillary gel-electrophoresis.

### Clinical scoring

Inflammation and necrosis were clinically assessed as described by Reiner et al. 2019. To ensure comparability with other studies, the piglets were scored on the 3rd day of life. Clinical signs were clearly visible during this period in all previous studies, and the piglets were not yet as much exposed to environmental effects like weaners and fatteners. To minimize the animal load, clinical signs were recorded using a digital camera (Canon EOS DC 8.1 V, Canon) according to a standardized scheme for later detailed evaluation of the images (Windows Media Player, Version 12, Microsoft GmbH, Germany).

Clinical alterations in the tail base and tail tip, the ears, the teats and navel, coronary bands, wall horn, heels and sole of the feet as well as the face were assessed individually. The following clinical characteristics were considered and scored 0, if the sign was not visible or 1 if the sign was visible (Table 1). The tail base and tail tip were independently scored for loss of bristles, swelling, redness, scab formation (tail tip only), rhagades, exudation, necrosis, bleeding (tail tip only), and ring-shaped constrictions (tail tip only). Ears were scored for a shiny skin, the loss of bristles, necrosis and congested ear veins. The face was scored for the absence or presence of edema at the eye lids and nose back. Teats were scored for swelling, reddening, scab formation, necrosis and congested blood vessels. The navel was scored for signs of inflammation in the form of redness or swelling. Claws were scored qualitatively for any signs of inflammation at the coronary bands (swelling, redness or exudation), wall bleeding, swelling and bleeding of the heels. The examined binary scores are presented by organ system as percentage of affected piglets.

**Table 1:** Overview of the collected individual characteristics and scores

	Skin shiny	No bristles	Swel-ling	Red-ness	Scab Forma-tion	Rhagades	Exuda-tion	Necrosis	Bleeding	Ring-shapedconstric-tions	Veinconges-tion	Edema		Additive body part scores	Additive SINS score
Tail base	Ns	0/1^c^	0/1	0/1	ns	0/1	0/1	0/1	ns	ns	ns	ns	➜	0–6	
Tail tip	Ns	0/1	0/1	0/1	0/1	0/1	0/1	0/1	0/1	0/1	ns	ns	➜	0–9	
Ears	0/1	0/1	ns	ns	ns	ns	ns	0/1	ns	ns	0/1	ns	➜	0–4	
Face^a^	ns	ns	ns	ns	ns	ns	ns	Ns	ns	ns	ns	0/1	➜	0–2	
Teats	ns	ns	0/1	0/1	0/1	ns	ns	0/1	ns	ns	0/1	ns	➜	0–5	0–36
Navel	ns	ns	0/1	0/1	ns	ns	ns	Ns	ns	ns	Ns	ns	➜	0–2	
Coronary bands^b^	ns	ns	0/1	0/1	ns	ns	ns	Ns	ns	ns	ns	ns	➜	0–2	
Claw wall^b^	ns	ns	ns	ns	ns	ns	ns	Ns	0/1	ns	ns	ns	➜	0–2	
Heels^b^	ns	ns	0/1	ns	ns	ns	ns	Ns	0/1	ns	ns	ns	➜	0–4	

ns: not scored; ^a^eye lids and nose back are separately scored; ^b^hind and front limbs are separately scored; ^c^present (1) or absent (0)

All findings were summed up to give an additive body part score (Table 1). Scores could reach 2 to 9 points. All scores were summed up for the SINS score in an unweighted manner. This resulted in possible SINS scores between 0 and 36 for each piglet. All scores were assigned by two experienced persons together. An overview on the evaluated phenotypes is given in Table 1. Additionally, the SINS score was used after Z-transformation (ZSINS). For the descriptive presentation of the phenotypes of the progeny from the three boars, a generalised linear model with boar as effect was used in the case of binary data (Supplemental Table 1). The metric data (Table 2) were calculated using Anova, considering the boar as effect.

**Table 2:** Body part scores of suckling piglets by boar

Scores	Offspring	*N*	Mean	± SE	LCI95	UCI95	Min	Max	*P*_boar_	*R*^2^
Tail base	All	234	1.1	0.1	1.0	1.3	0	3	< 0.001	6.7
	DU boar^a^	48	0.8	0.1	0.5	1.1	0	3		
	PI + boar^a^	77	0.9	0.1	0.7	1.2	0	3		
	PI− boar^b^	109	1.5	0.1	1.2	1.7	0	3		
Tail tip	All	234	0.7	0.1	0.6	0.8	0	5	0.008	4.1
	DU boar^a^	48	0.4	0.1	0.2	0.6	0	3		
	PI + boar^a^	77	0.6	0.1	0.4	0.7	0	4		
	PI− boar^b^	109	0.9	0.1	0.7	1.2	0	5		
Ears	All	234	2.3	0.1	2.1	2.4	0	3	< 0.001	15.8
	DU boar^a^	48	1.5	0.2	1.2	1.8	0	3		
	PI + boar^b^	77	2.5	0.1	2.3	2.7	0	3		
	PI− boar^b^	109	2.4	0.1	2.3	2.6	0	3		
Face	All	230	1.0	0.0	1.0	1.1	0	2	0.494	0.6
	DU boar	47	1.1	0.1	1.0	1.2	0	2		
	PI + boar	77	1.0	0.0	0.9	1.1	0	2		
	PI− boar	106	1.0	0.0	0.9	1.1	0	2		
Teats	All	234	0.8	0.1	0.7	0.9	0	5	< 0.001	7.1
	DU boar^a^	48	0.3	0.1	0.1	0.5	0	2		
	PI + boar^b^	77	0.9	0.1	0.7	1.0	0	3		
	PI− boar^b^	109	0.9	0.1	0.7	1.1	0	5		
Coronary bands	All	230	1.1	0.1	1.0	1.2	0	2	0.001	5.8
	DU boar^a^	47	1.0	0.1	0.7	1.2	0	2		
	PI + boar^a^	75	0.9	0.1	0.7	1.0	0	2		
	PI− boar^b^	108	1.3	0.1	1.1	1.4	0	2		
Wall	All	230	1.7	0.0	1.6	1.8	0	2	0.180	1.5
	DU boar	47	1.6	0.1	1.4	1.8	0	2		
	PI + boar	75	1.8	0.1	1.7	1.9	0	2		
	PI− boar	108	1.7	0.1	1.6	1.8	0	2		
Heels	All	233	3.9	0.0	3.8	3.9	0	4	0.012	3.8
	DU boar^a^	48	3.6	0.1	3.3	3.9	0	4		
	PI + boar^b^	76	3.9	0.0	3.9	4.0	2	4		
	PI− boar^b^	109	3.9	0.0	3.8	4.0	0	4		
SINS	All	226	12.7	0.2	12.3	13.2	0	22	< 0.001	14.4
	DU boar^a^	46	10.4	0.6	9.3	11.6	0	18		
	PI + boar^b^	75	12.7	0.3	12.1	13.2	7	18		
	PI− boar^c^	105	13.8	0.3	13.2	14.4	0	22		

^a,b,c^Offspring groups with different letters are statistically significantly different at *P* <  = 0.05. *SE* standard error; *LCI95* lower 95% confidence interval, *HCI95* higher 95% confidence interval. *DU* Duroc boar, PI + : favourable Pietrain boar; PI−: unfavourable Pietrain boar. *P*_Boar_: significance of boar effect. *R*^2^ coefficient of determination

### DNA extraction and sequencing

DNA was extracted from the piglet’s docked tails using the smart DNA prep (m) kit (Analytik Jena, Jena, Germany) and quantified by Qubit Flex Fluorometer (Invitrogen, Thermo Fisher Scientific, Waltham, MA, USA) using the Qubit dsDNA broad range assay kit (Invitrogen, Thermo Fisher Scientific, Waltham, MA, USA). The DNA was diluted to a uniform concentration of 50 ng/μl. During library preparation, the samples were prepared to be compatible for sequencer processing. The paired-end library generated in this process was amplified.

Complete genome sequencing was performed using the Illumina NextSeq500/550 v2 and Illumina NovaSeq 6000 (Illumina, San Diego, USA). In this process, 150 bp paired-end reads were generated with a coverage of 15x.

### Bioinformatics workflow

The files received from the sequencing company were decoded and converted from.bz2 to gzipped fastq files.

### OVarFlow pipeline

The available raw data were transferred to the open source workflow OVarFlow for further bioinformatic analysis. This workflow is used for variant discovery of single nucleotide polymorphisms (SNPs) and indels, insertions and deletions in model and non-model organisms (Bathke and Lühken 2021). The workflow enables automation, documentation and the associated reproducibility of the individual evaluation steps.

As part of the preparation of the workflow, the required input files were compiled in a configuration file in comma separated values (CSV) format. This file contains the reference genome and the annotation (Sus scrofa 11.1, Genebank Assembly Accession: GCA_000003025.6), the min sequence length (value = 1) and sample information on the Illumina short read sequencing data used in the analysis.

The reads were undergone quality control using FastQC version 0.11.9 (https://www.bioinformatics.babraham.ac.uk/projects/fastqc/). Mapping to the reference genome was done using the mem algorithm introduced in the Burrows Wheeler Alignment Software (version 0.7.17-r1188; Li et al. 2013). The compressed BAM files were created by piping directly into Samtools version 1.11 (http://www.htslib.org/). These files were passed to gatk SortSam (https://gatk.broadinstitute.org/hc/en-us/articles/360051307011-SortSam-Picard-) and sorted in the next step. MarkDuplicates (https://gatk.broadinstitute.org/hc/en-us/articles/360051306171-MarkDuplicates-Picard-) was used to locate and mark multiple reads.

### Variant calling

OVarflow performs variant detection at several intervals per individual to enable the highest possible degree of parallelisation. The actual variant calling was done using gatk Haplotype Caller (https://gatk.broadinstitute.org/hc/en-us/articles/360050814612-HaplotypeCaller). First, active regions were identified in which a significant number of reads showed variations beyond the expected background noise. De Bruijn-like graphs were created for these regions which were used to recreate a possible sequence and develop haplotype candidates. To narrow down potential sites of variation, each possible haplotype was aligned with the reference sequence using the Smith-Waterman algorithm. The existing reads were then aligned with each of the possible haplotypes and a matrix was created with the expected likelihoods of occurrence. Therefore, the PairHMM algorithm was used. The assignment of the most likely genotypes for the available samples was done according to Bayes’ theorem.

The individual Genome Variant Call Format (GVCF) files of the analysed intervals, which were output after variant calling, were recombined for each individual and then merged into an aggregated file containing the variant information for all analysed individuals. Embedded in the workflow, gatk GatherVcfs (https://gatk.broadinstitute.org/hc/en-us/articles/360050814232-GatherVcfs-Picard-) and gatk CombineGVCFs (https://gatk.broadinstitute.org/hc/en-us/articles/360050815372-CombineGVCFs) were used for this purpose.

With gatk GenotypeGVCFs (https://gatk.broadinstitute.org/hc/en-us/articles/360050816072-GenotypeGVCFs), the data preprocessed with HaplotypeCaller were subjected to an additional joint genotyping across several individuals. The genotype information lost during the combination of the GVCF files was restored and the genotyping accuracy was improved.

The separation of the detected SNPs and indels into separate files was performed with gatk SelectVariants (https://gatk.broadinstitute.org/hc/en-us/articles/360051305531-SelectVariants).

### Quality control

In order to detect false positive variants of the variant call set and remove them, a hard filtering according to GATK was performed. For SNPs and indels, different thresholds of the filters in gatk VariantFiltration (https://gatk.broadinstitute.org/hc/en-us/articles/360050815032-VariantFiltration) were chosen. The following filters were passed through (filter name, filter, parameters for SNPS and indels):

I. ‘QD2’: The quality by depth (QualByDepth, QD), Assessment of the quality score (Qual) in relation to the present sequencing depth (Depth); SNPs: QD < 2.0, Indels: QD < 2.0.
II. ‘QUAL30’: general quality score (Qual); SNPs: Qual < 30, Indels: QUAL < 30.0
III. ‘SOR3’: Determination whether there is allele-specific strand bias, estimated by the symmetrical odds ratio test (StrandOddsRatio; SOR); SNPs: SOR > 3.0
IV. ‘FS60’/‘FS200’: detection of strand bias by Fisher exact tests and output as phred-scaled *P*-value (FisherStrand, FS); SNPs: FS > 60.0, Indels: FS > 200.0
V. ‘MQ40’: the root mean square (RMS) mapping quality of reads across samples (MQ); SNPs: MQ < 40.0
VI. ‘MQRankSum-12.5’: the Mann–Whitney–Wilcoxon rank sum test for the mapping quality of reference and alternative reads (MappingQualityRankSumTest, MQRanksum); SNPs: MQRankSum < -12.5
VII. ‘ReadPosRankSum-8’/‘ReadPosRankSum-20’: Allele-specific rank sum test according to Mann, Whitney and Wilcoxon for the relative positioning of the reference allele versus the alternative allele within the reads (ReadPosRank-SumTest, ReadPosRankSum); SNPs: ReadPosRankSum < − 8.0, Indels: ‘ReadPosRankSum < − 20.0

The variants that did not pass the filter criteria were marked.

Following the quality control, the split SNP and indel files were combined with gatk SortVcf (https://gatk.broadinstitute.org/hc/en-us/articles/360050815732-SortVcf-Picard-) again. The tool gatk SelectVariants removed the previously marked variants.

### Variant effect prediction

The functional annotation of the previously found genetic variants was carried out using snpEff, version 5.0 (Cingolani et al. 2012). Information on genetic coordinates and the effect of the respective variant is output, for instance if the variant is located in a coding region of a gene and whether it is a synonymous, non-synonymous or nonsense mutation.

### Genome-wide association study (GWAS)

The preparation of the data output by OvarFlow for the GWAS and the actual execution of the GWAS was done in R, version 4.2.1 (R Core Team 2022). RStudio (RStudio Team 2022) was used as the graphical user interface.

The annotated VCF file output at the end of the OVarFlow workflow was converted to binary PLINK format using the PLINK package (Purcell et al. 2007; version 1.9) with the option that only SNPs are included. The first quality control of the genotype data were done with PLINK. Only variants and individuals that passed the following criteria were included in the GWAS:

- Missingness per marker < 0,01
- Missingness per individual < 0,1
- Minor-Allele-Frequency (MAF) > 0,05
- Hardy–Weinberg equilibrium (HWE) p > 0.000001

With a special focus on the X chromosome, a second quality control was carried out with GenABEL version 1.0.0 (Aulchenko et al. 2007). This should detect incorrect heterozygous male X-linked genotypes and exclude them from the analysis.

A reduced SNP dataset was created in order to check for any population structure that might be present. SNP pruning was based on linkage disequilibrium and pairwise genotypic correlation using PLINK. With the reduced dataset, a principal component analysis (PCA) was performed using TASSEL 5.0 (Bradbury et al. 2007). Tassel 5.0 was also used for kinship analyses creating genetic distances between offspring of the three boars.

The actual GWAS was conducted by GAPIT (Wang and Zhang 2021; version 3) using the BLINK model (Huang et al. 2019). For this purpose, the genotype data were converted from PLINK to hapmap format using TASSEL 5.0. Bonferroni-corrected genome-wide and chromosome-wide significance thresholds were given for a significance level of *α* = 0.05.

For GWAS, effects of boar, sow (litter), contemporary group, sex, and PCA were used as fixed effects and birth weight was used as covariate. For PCA, 4 principal components were considered. Because each sow was used only once in the study, effects of litter and sow were identical.

Manhattan plots and quantile–quantile plots were generated using the R package qqman, version 0.1.8 (Turner 2018).

After GWAS, associations were excluded from further analysis, if one of the expressions of the characteristics (0 vor 1) was represented by less than 5% of the cases (i.e., 12 out of 234 animals).

### Statistical analysis of SNP effects on SINS characteristics

The effects of the genotypes of the significant SNPs from GWAS on the SINS characteristics were tested with Anova (metric data) and with a General Linear Model (bivariate data) in IBM-SPSS, version 27 (Statistical package for Social Sciences, IBM, Munich, Germany). SNPs were only included if they had at least three genotypes, if the effect was additive, i.e., if the heterozygote value did not exceed the value of the highest homozygous or fall below the value of the lowest genotype by more than 20%, and if the negative decadic logarithm of significance exceeded 8.4. We tested not only the effects of SNPs on the phenotypes with significant association in GWAS, but on all SINS genotypes. The rationale for this approach was the assumption that due to the syndrome character of SINS, involved gene loci should affect different SINS characteristics at the same time.

### Kandidate gene prediction

Positional candidate genes were identified based on their distance from SNPs. The Genome data viewer (https://www.ncbi.nlm.nih.gov/genome/gdv/?org=sus-scrofa) based on release Sscrofa11.1 (GCF_000003025.6) was used for this purpose. Positional candidate genes were selected as such if they were located no further than 1 Mbp from the SNP in either direction.

Information on positional candidate genes were obtained with GeneCards (Safran et al. 2022) human gene data base. This program was used to detect genes involved in inflammation, vasculitis and necrosis. Genes were displayed in order of the GeneCards Score, were best fitting genes obtain the highest order.

### KASP

To confirm the genotypes of the 234 animals that were whole genome sequenced, 8 SNPs were selected from the set of predicted candidate genes and genotyped using KASP. With the intention of randomly checking the results of the GWAS, not all SNPs were selected. The specific selection of the 8 SNPs was based on their proximity to candidate genes (see Table 5) and their significance level.The KASP assay technology is based on competitive allele-specific PCR and allows bi-allelic scoring of SNPs and indels at specific loci.

For sample preparation, DNA was uniformly diluted to a concentration of 50 ng/μl and 25 μl were placed in 96-well plates. The actual genotyping was performed in the laboratory of LGC Genomics (Hoddesdon, United Kingdom). The primers were designed according to the rs numbers and sequences covering the range of 50 bases around the polymorphism that we had provided for the different SNPs.

In the initial PCR cycle, the matching allele-specific forward primer binds to the target region together with the common reverse primer. During amplification, the tail sequence located at the 5′-end of the primer is added to the newly synthesised strand. In the following cycles, further amplification of these takes place.

The KASP master mix used for the assay contains universal FRET (fluorescence resonance energy transfer) cassettes with Fam- or Hex-labelled regions. These regions correspond to one of the allele-specific tail sequences and enable their binding. In this case, the FRET cassette is no longer quenched and emits the corresponding fluorescence signal. If the genotype of the examined animal is homozygous at the SNP, only one out of two possible fluorescence signals is generated. In case of heterozygosity, a mixed fluorescence signal can be detected. The effects of the genotypes predicted by KASP were analysed by onefactorial variance analysis (Anova) in metric and with a generalised linear model in bivariat data.

## Results

### Phenotypes

More than 86% of the three-day-old suckling piglets already showed swelling and bleeding of the heels and haemorrhages in the claw wall (Supplemental Table 1). The piglets were affected by eyelid oedema at the same level. The ears showed vein congestion in 86% and a shiny surface and bristle loss in over 65% of the animals. About 40% to 60% of the piglets showed swelling and redness of the tail base, venous congestion at the teats and signs of inflammation at the coronary bands of the front and hind limbs. Over 20% of the piglets had no bristles at the tail base, scab formation at the tail tip and swelling at the teats. As expected, the more severe symptoms, such as bleeding and necroses, were only detectable in 4 to 8% of the piglets at this age (Supplemental Table 1).

Any of the additive body part scores except for face and claw wall showed a significant effect of the boar. There were significant differences in the SINS scores in offspring groups from all three boars (*P* = 2.9 × 10^–8^) (Table 2). The effect of the boar explained up to 14% of the phenotypic variance. In 56% of the phenotypic characteristics, phenotypes in offspring of Pietrain boars previously classified as unfavourable (PI−) were significantly worse than in DU-offspring (DU) and in 27% they were significantly worse than in offspring of Pietrain boars classified as favourable (PI +). In 29% of the cases, the DU-offspring were also significantly superior to the offspring of the favourable Pietrain boars (PI +) (Supplemental Tables 1 and 2).

With the exception of the face and the navel, the summarized scores of the individual body parts were significantly correlated with each other in accordance with the syndrome characteristics and correlations with the SINS score were between 0.3 and 0.62. Only the heels were correlated to a lesser degree with other SINS features (Table 3).

**Table 3:** Spearman correlations between phenotypic body part scores

	Ear	Face	Tail base	Tail tip	Navel	Teats	Coronary bands	Claw wall	Heels	SINS
Ear		0.061	0.252**	0.114	0.04	0.246**	0.183**	0.208**	0.250**	0.616**
Face			0.099	0.049	0.065	− 0.074	0.12	0.211**	0.130*	0.204**
Tail base				0.105	− 0.097	0.212**	0.004	0.310**	0.089	0.635**
Tail tip					0.043	0.167*	− 0.025	− 0.261**	0.076	0.376**
Navel						0.079	0.111	− 0.075	0.033	0.139*
Teats							0.04	0.009	0.05	0.523**
Coronary bands								0.298**	0.292**	0.439**
Claw wall									0.280**	0.399**
Heels										0.299**

*P <  = 0.05; **P <  = 0.01

### GWAS

**Fig. 1:**
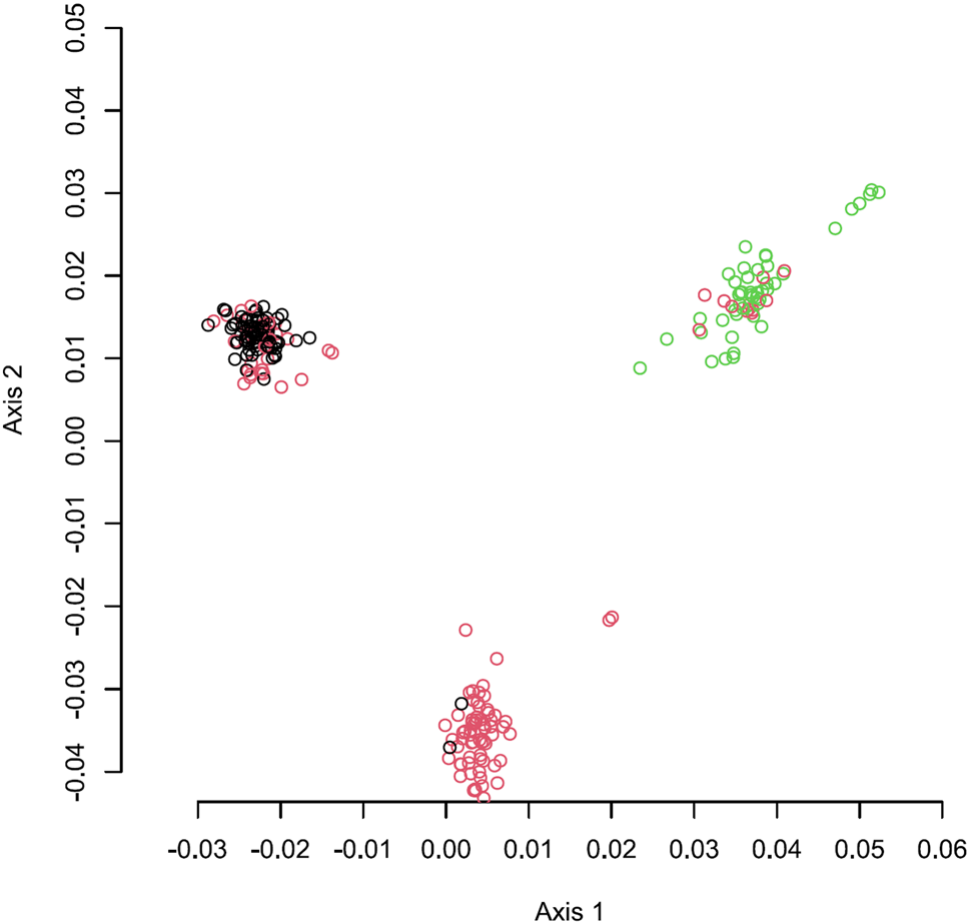
Principal Coordinates Analysis (PCA)-Plot based on the distance matrix as calculated with Tassel 5.0. Black = PI + offspring, red = PI− offspring, green = DU-offspring. Axis 1 and 2 represent the two main principal components creating genetic distances between offspring of the three boars

There was a significant stratification of the data (Fig. 1) which was addressed by the inclusion of PCA data into the statistical model.

Associations were excluded from further analysis, if one of the expressions of the characteristics (0 or 1) was represented by less than 5% of the cases (i.e., 12 out of 234 animals). GWAS identified 221 significant SNPs, from which 56 were chromosome-wide (*P* ≤ 6.4) and 165 were genome-wide significant (*P* ≤ 8.4) (Supplemental Table 2). With few exceptions, the SNPs were already known and listed under their rs-ID. These SNPs were associated with 25 different phenotypic signs. Seventy SNPs were closer to each other than 10^6^ basepairs. They were condensed into 71 chromosomal regions, e.g., 1, 1 to 1, 9 on SSC1 (Supplemental Table 3). Twentynine of the SNPs were associated with more than one phenotype (Supplemental Table 4).

**Fig. 2:**
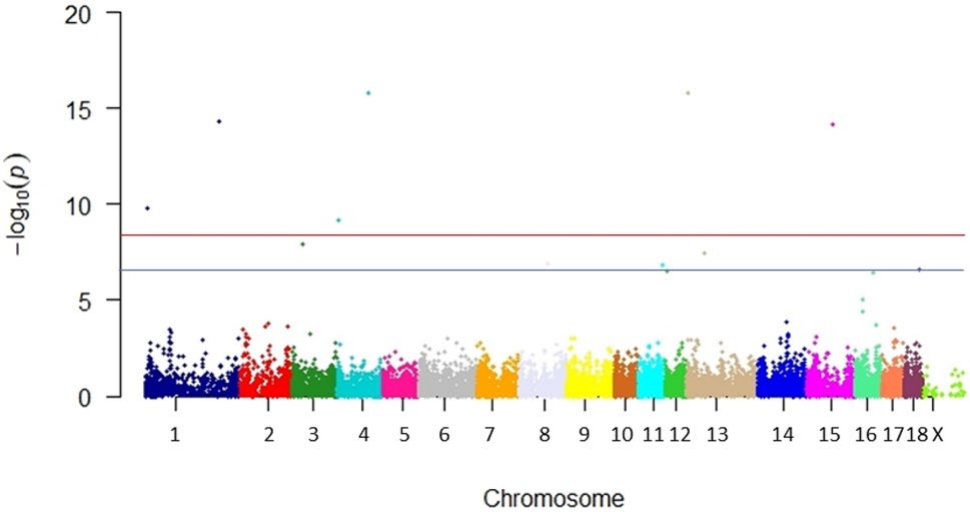
Manhattan plot of GWAS with *P*-values for Z-transformed SINS scores. Negative decadic logarithm of the significance of SNPs in the genome-wide association study. The blue and red lines indicate chromosome-wide and genome-wide significance, respectively

**Fig. 3:**
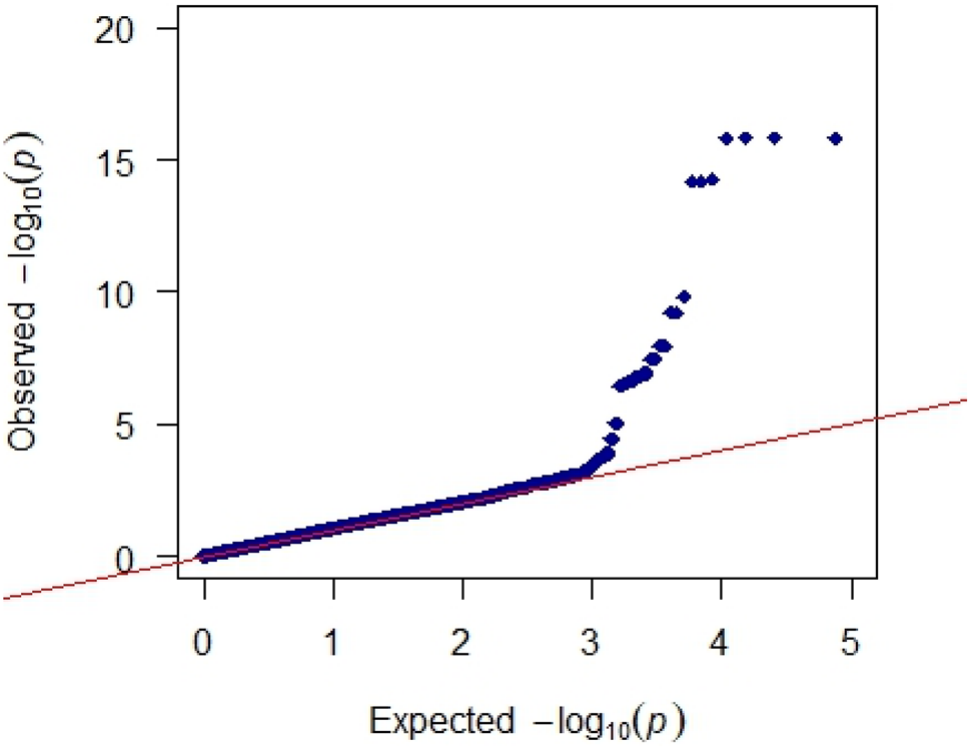
Q-Q plot of GWAS p-values Z-SINS (Z-transformed SINS scores)

The Manhattanplot of Fig. 2 summarizes the effects of SNPs in association with the SINS score. Details are given in Supplemental Table 2. Effects were found on numerous chromosomes. The QQ plot and the genomic inflation factor (*λ* = 1.09) did not indicate population stratification and/or cryptic relatedness between animals (Fig. 3).

### Anova

Significant SNPs in GWAS were assigned to the responsible genotypes. The distribution of genotypes as well as mean values and standard error of the respective phenotype expressions can be found in Supplemental Table 5. Because some SNPs were significantly associated with several phenotypes of the syndrome, a total of 203 significant relationships could be found and the favorable and unfavorable genotypes for the respective SNPs could be derived. The SNPs explained between 14.7 and 30.7% of the phenotypic variance. The corresponding negative decadal logarithms of significance were between 8.1 and 18.6.

**Fig. 4:**
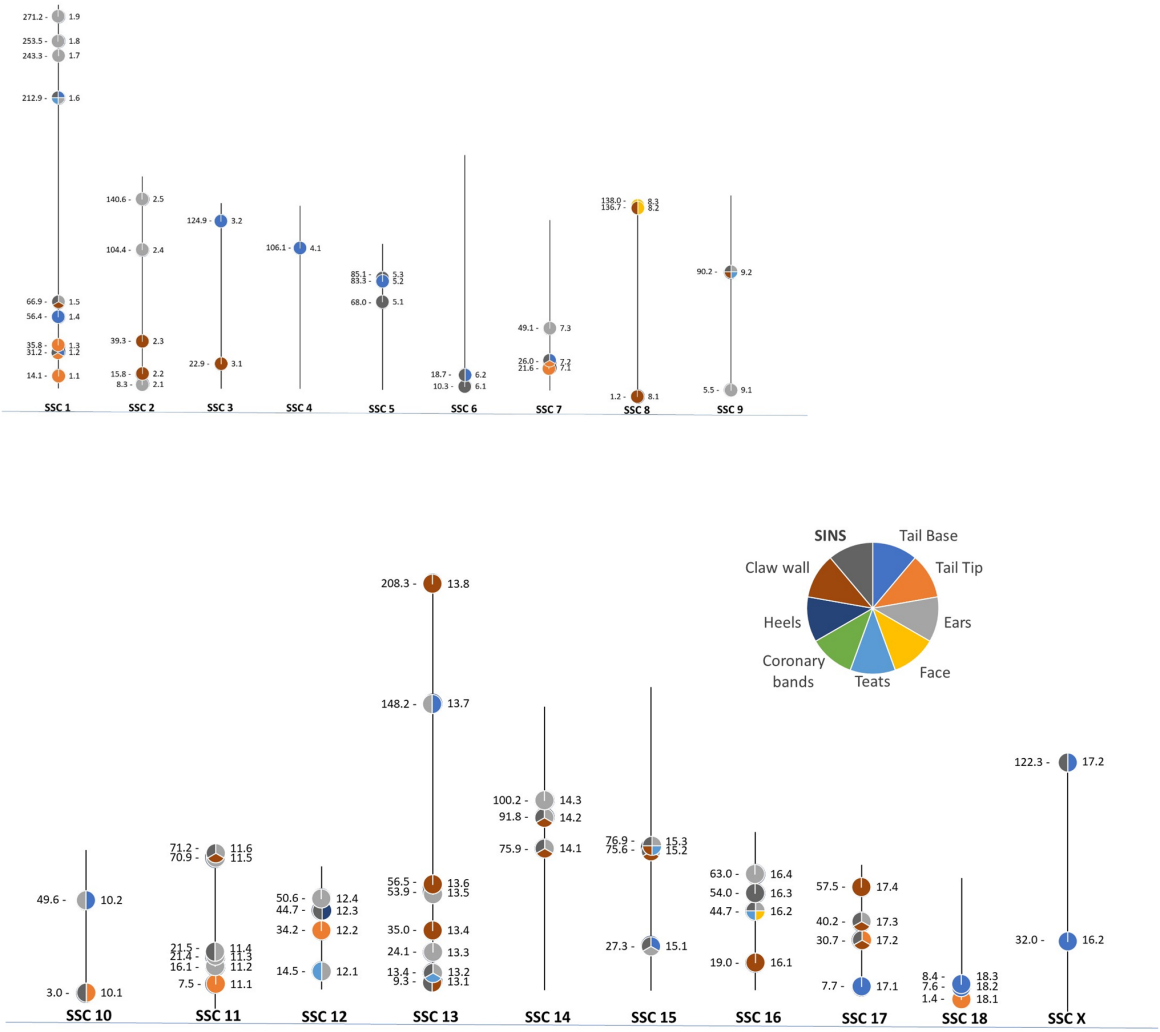
Distribution of significant SNPs with association to the different SINS genotypes across. The vertical lines characterise the extent of the 18 autosomes and the X chromosome in pigs (in Mbp). The pie charts on the lines correspond to the location of the SNPs with association to the SINS signs. The colours correspond to the indications in the legend. In the area of pie charts with several colours, there are associations with inflammation/necrosis in the area of several body parts. To the left of the vertical are the positional data in Mbp, to the right the numbers of the identified 71 chromosomal regions with significant associations in the GWAS

In 42 of these associations GWAS and ANOVA phenotypes were identical. In a further 76 associations GWAS and ANOVA traits represented the same body part. Several SNPs were associated with more than one body part and different signs of SINS. An overview of the localization of the most important significant SNPs in the genome including the associated phenotypes is shown in Fig. 4. The SNPs did not occur singularly but were distributed over the entire genome. In the area of numerous chromosomal regions (numbers right to the chromosome), associations with several signs of SINS were found.

Signs at the ears and claw wall were most often associated with SNPs (86 and 44 times, respectively). Tail base (*n* = 24) and SINS score (*n* = 25) were also associated with numerous SNPs. Fewer associations were detected for tail tip, teats, face and heels.

### Verification of selected SNPs by KASP

**Fig. 5:**
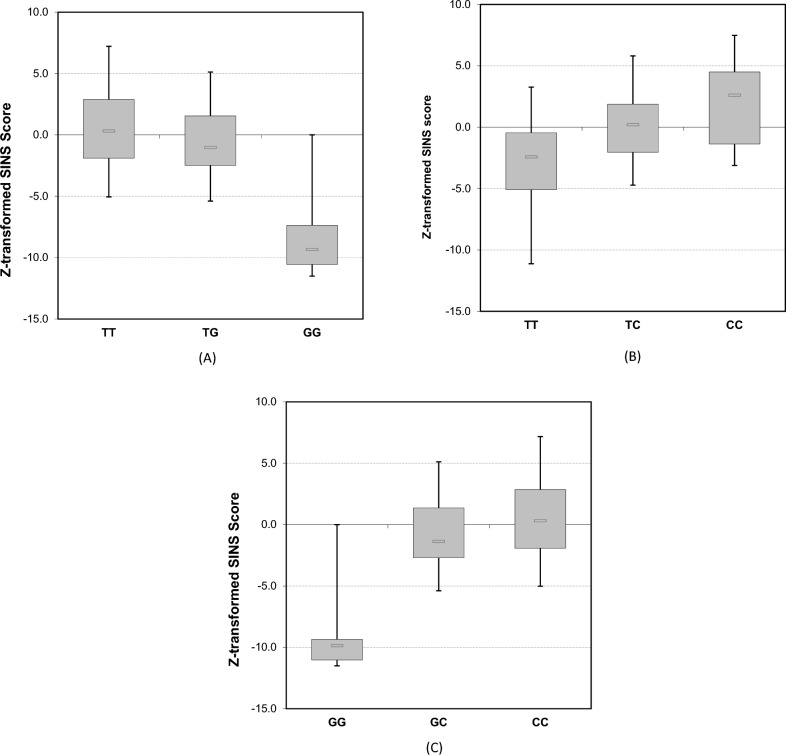
Exemplary effects of selected SNPs on SINS phenotypes. The box-plots with whiskers represent the distribution of 90% of the piglets’ values. **A** SNP 9_90241577 (SSC_position); TT: *n* = 166; TG: *n* = 53; GG: *n* = 7. **B** SNP 12_44738423 (SSC_position); TT: *n* = 47; TC: *n* = 119; CC: *n* = 59. **C** SNP 15_76941405 (SSC_position); GG: *n* = 5; CG: *n* = 53; CC: *n* = 168

**Fig. 6:**
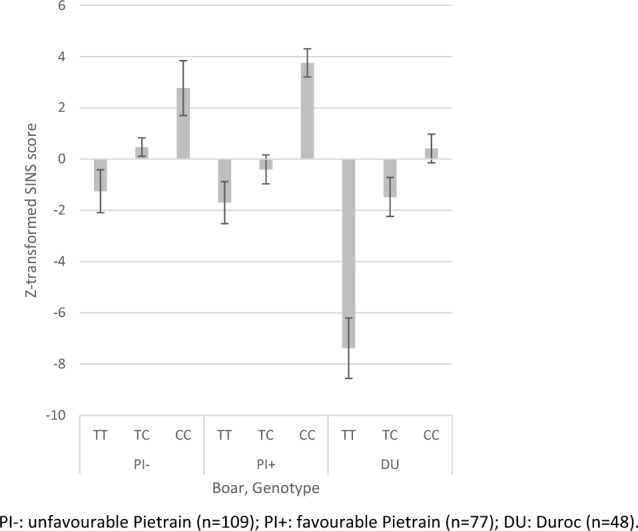
Exemplary effects of SNP 12_44738423 on Z-transformed SINS scores by boar progeny. PI−: unfavourable Pietrain (*n* = 109); PI + : favourable Pietrain (*n* = 77); DU: Duroc (*n* = 48)

Eight SNPs on SSC 9, 12, 13, 14, and 15 were selected for verification of the genome-wide sequencing results by KASP. Differences in genotypes between genomic sequencing and KASP were found in 1.6% of the individuals. They represented exchange between the major homozygote and the heterozygote genotype. Accordingly, there were no differences between sequencing and KASP in the effects of alleles on phenotypes. The 95% confidence intervals for e.g., SINS of the SNP 12_44738423 genotypes were clearly separated (TT: 10.6–13.3; CT: 15.4–16.9; CC: 17.5–20.1) (Table 4). The effects of this and two further SNPs are exemplarily shown in Fig. 5A–C. Additionally effects of distinct SNPs could be found in the offspring of all three boars, although to different degrees (Fig. 6).

**Table 4:** Verification of SNP-results by KASP with selected SNPs

SNP	Trait	Mean			95% CI			*P*
		Genotype/number			Genotype			(− log)
SSC12_44738423		TT/47	CT/119	CC/59	TT	CT	CC	
	Z-SINS	− 3.1	0.2	2.2	− 4.2−1.9	− 0.4–0.8	1.2–3.2	11.1787456
SSC13_208309290		GG/194	GA/29	AA/1	GG	GA	AA	
	Claw wall score	1.8	1.0	0.0	1.8–1.9	0.7–1.3	0	12.9146093
SSC9_5511145		AA/163	TA/63	TT/1	AA	TA	TT	
	Ear Score	2.7	1.6	0.0	2.5–2.8	1.3–1.9	0	10.4909981
SSC9_90241577		TT/166	TC/53	CC/7	TT	TC	CC	
	Z-SINS	0.6	− 0.6	− 9.0	0–1.1	− 1.6–0.4	− 11–6.9	9.62529167
	Ear Score	2.6	1.8	0.3	2.5–2.8	1.5–2.1	− 0.2–0.7	12.2498954
	Claw wall score	1.8	1.8	0.1	1.7–1.9	1.6–1.9	− 0.2–0.5	11.6720769
SSC14_75918198		GG/165	AG/53	AA/5	GG	AG	AA	
	Z-SINS	0.6	− 0.7	− 9.5	0–1.2	− 1.7–0.2	− 11.3–− 7.6	8.21856566
	Ear Score	2.7	1.7	0.2	2.5–2.8	1.4–2	− 0.4–0.8	12.4989599
	Claw wall score	1.8	1.8	0.2	1.7–1.8	1.7–2	− 0.4–0.8	7.78305613
SSC15_76941405		GG/5	CG/53	CC/168	GG	CG	CC	
	Z-SINS	− 10.1	− 0.7	0.6	− 11.3–− 9	− 1.7–0.2	0–1.1	9.05809803
	Ear Score	0.0	1.7	2.7	0–0	1.4–2	2.5–2.8	13.3111565
	Claw wall score	0.0	1.8	1.7	0–0	1.7–2	1.7–1.8	9.46548588
SSC15_76926106		GG/159	AG/60	AA/1	GG	AG	AA	
	Ear Score	2.7	1.6	0.0	2.5–2.8	1.3–1.9	0	10.4089183
SSC14_91808934		TT/167	CT/53	CC/6	TT	CT	CC	
	Z-SINS	2.7	1.7	0.2	2.5–2.8	1.4–2	− 0.3–0.6	14.1030788
	Teat score	1.8	1.8	0.2	1.7–1.8	1.7–2	− 0.3–0.6	9.60846104

### Positional candidate genes

Of over 11,000 genes linked to inflammation, necrosis and vasculitis functions in GeneCards, 2300 were within 5 Mbp of a significant SNP. Fortynine genes were located no further than 100 kbp from a SNP and three no further than 10 kbp (data not shown). Of the genes located within a range of at most 100 kbp from a significant SNPs, 15 with direct involvement in inflammation, vasculitis, and necrosis were defined as candidate genes (Table 5). 281 Genes located within a distance of 500 kbp are additionally shown in Supplemental Table 6.

**Table 5:** Kandidate genes involved in inflammation, vasculitis and necrosis (according to GeneCards) in the proximity of SNP markers

Gene symbol	NCBI gene ID	SSC	Start	Stop	SNP position	Distance from SNP (bp)	Anno. effect
RTN3	100512087	2	8291750	8354920	8270586	52749	Intergenic region
F2	100144442	2	15793257	15819151	15794456	11749	Downstream gene variant
FAH	100623036	7	49047833	49087790	49086851		Intron 13 variant
TRIM68	100738170	9	5502931	5516936	5511145*	− 1211	3 ‘ UTR variant
DNAH11	100620543	9	90420044	90768327	90241577*	352609	Intergenic region
SLC13A2	100628018	12	44733106	44753898	44738423*		Intron 3 variant
RFTN1	100155924	13	3454448	3675072	3498072		Intron 7 variant
ZCWPW2	100627543	13	14785464	14927384	148170559		Intron 1 variant
SLC19A1	100579176	13	207986094	208007855	208809290*	− 312316	Intergenic region
NUDT3	100737442	14	375918056	75958187	75918198*		Exon 1 silent mutation
P4HA1	100037299	14	75827207	75919121	75918198*		Intron 1 variant
CXCL12	494460	14	91516383	91543857	91808934*	− 278814	Intergenic variant
ERICH2	100155783	15	76939796	76972605	76941405*		Intron 1 variant
KLHL23	100520221	15	76029145	76051670	76926106*	461803	Intergenic reagion
DOCK2	100512021	16	53843248	54248708	53952975		Intron 24 variant

*SNP was verified by KASP (see Table 4)

## Discussion

SINS is a syndrome in pigs characterized by inflammation and necrosis of various parts of the body that can lead to pain, suffering and damage. Various studies showed symptoms at the base of the tail, tail tip, ears, teats, navel, coronary bands, claws and heels (for review see Reiner et al. 2021a). The syndrome starts with inflammatory loss of bristles, swelling, and redness. Later, rhagades, exudation, and possibly necrosis occure. The inflammation was detected histopathologically in newborn piglets, suckling piglets, weaners and fattening pigs (Reiner et al. 2020; Kühling et al. 2021a, b). Severe vasculitis, vascular thrombosis and lymphoplasmacellular inflammation (Kühling et al. 2021a) seem to be associated with a shortage of supply of the downstream tissues (Reiner and Lechner 2019) in the sense of ischaemia as already proposed by Penny et al. (1971) and Blowey and Done (2003).

Observations in the field that offspring of different boars developed SINS to a significantly different extent under the same husbandry conditions were reproduced in a targeted mating experiment under station conditions (Kühling et al. 2021b). Already in these experiments, the boars in question were used as mixed semen simultaneously on the same sow to minimize environmental effects. The heritability for SINS was recently estimated at 0.2 in a very informative study (Leite et al. 2023). For the current study, three extreme boars were selected from the study of Kühling et al. (2021b) and used again on a different sow herd (again as mixed semen). This experimental design was intended to increase the variability of SINS symptomatology and comparability of the boars used, while minimizing the number of experimental animals required. Indeed, the boars′ effects regarding SINS of their offspring could be reproduced and repeated. However, a weakness of the study arises from the selection of 3-day-old suckling piglets. This was thought to minimize environmental effects that increase with age (Reiner et al. 2020). However, it was also clear that, although massive inflammatory symptoms would occur in these young animals, the severe forms, such as exudation and necrosis, would be less frequent. In the end, some forms of necrosis to organ systems occurred so rarely that they could not be considered in GWAS.

The degree of overlap of symptoms in different parts of the body was striking in recent as well as in the present study. The simultaneous occurrence in such different parts of the body, the evidence of vasculitis, thrombosis, and intimal proliferation with intact epidermis, and the histopathologic evidence of granulocytes, macrophages, and lymphocytes in the affected tissue in newborn piglets argues for the endogenous genesis of the disease. Thus, the syndrome initially occurs independently of external factors such as biting or technopathies, although it can be modified by environmental conditions later in its course (Reiner et al. 2021a).

The importance of inflammation was also confirmed at various levels of clinical chemistry, metabolic, and transcriptomic findings (Löewenstein et al. 2021; Ringseis et al. 2021). Signs of inflammation were found in the liver of affected animals. In 3-day-old suckling piglets, mRNA levels of FGF21, haptoglobin, and IL-6 were elevated as a sign of the onset of an acute-phase. Increased ICAM1, TNF, and reduced IL-8 mRNA levels were indicative of stimulation of an inflammatory response (Ringseis et al. 2021). A significant increase in SOD1 mRNA can be interpreted as a response to oxidative stress by ischaemic injury. Overall, there was a consistent picture of increased numbers of monocytes and neutrophils, altered blood coagulation in weaners and thrombocytopenia in fatteners, as well as increased acute-phase proteins (especially C-reactive protein [CRP] and fibrinogen), altered serum metabolites and increased serum liver enzymes (Löewenstein et al. 2021).

221 SNPs associated with SINS-signs were mapped throughout the porcine genome. The significant SNPs explained between 10 and 35% of the phenotypic variance of the respective characteristics. These results support a polygenic architecture of SINS. It seems that a multitude of genetic variants could be involved in the phenotypic expression of the syndrome. Many of the SNPs were simultaneously associated with phenotypic variation in several traits. This is consistent with the general expectation for a syndrome in which different signs on multiple body parts are thought to be due to a common inflammatory cause. This aspect has already been extensively demonstrated in several experiments (see Reiner et al. 2021a, b).

However, SNPs were preferentially found in non-coding gene regions, so their function could not be easily inferred. Thus, they could be non-functional markers in linkage disequilibrium with the as yet unidentified functional gene variant, because of the relatively low coverage of the sequence data it is not to be expected that all existing gene variants were already detected by the present study. On the other hand, other high-throughput genomic studies during the last years show that around 90% of more than a hundred of gene variants associated with immune-mediated diseases that have been identified, are located in non-coding regions, making it difficult to assign them to molecular functions (Farh et al. 2015; Tak and Farnham 2015; Hindorff et al. 2009). Such SNPs are often associated with long non-coding RNAs which have been identified in farm animals (Kosinska-Selbi et al. 2020), but also in several inflammatory diseases, even in regard with the stimulation of human endothelial cells with lipopolysaccharide (LPS) (Castellanos-Rubio and Ghosh 2019), a model that hits the assumptions for the pathogenesis of SINS (Reiner et al. 2021a). In 2018, a database of over 10,000 lncRNAs was made available (lncRNAnet; Liang et al. 2018). However, screening with the SNPs of the current study did not match the listed lncRNAs. It remains for future studies to elucidate possible associations with the available SNPs and other, not yet described potential lncRNAs. Other forms of RNA, such as circular RNA (Yang et al. 2018), that can have regulatory effects on genes are also not excluded, but could not be investigated in the present study. On the other hand, 98% of the current SNPs of the present study are already known (Zhou et al. 2017; PigVar—The Pig Variations and Positive Selection Database; http://202.200.112.245/pigvar/index.jsp).

Thus, the identification of candidate genes was difficult. TRIM68 plays a critical role as a negative regulator of type I IFN production in viral and bacterial contact, which is demonstrated by the development of spontaneous inflammation and disease in mice lacking these proteins (Wynne et al. 2014). We discovered a 5 prime UTR premature start codon gain variant in TRIM68 on SSC9 that was associated with ear score and wall bleeding. Nothing is known about TRIM68 in pigs, but generally, TRIM68 turns off type I IFN production and thus reduces proinflammatory cytokine production. Thus, the discovered variation in TRIM68 might be involved in differing susceptibility to SINS, according to the hypothesis that SINS can be triggered by MAMPs leading to inflammation (e.g., Reiner et al. 2021a, b). F2 (Thrombin) is a candidate gene on SSC2 that is associated with SNPs for wall score, including wall bleeding and also ear vein congestion. F2 is involved in blood homeostasis, inflammation and wound healing (Glenn et al. 1988), which are important features in SINS (Löewenstein et al. 2021; Ringseis et al. 2021). CD96 as a candidate gene on SSC13 also seems to be involved in inflammation (e.g., LPS-mediated) and immune response (Gaudet et al. 2011). This chromosomal region was associated with inflammation in tail base and ears.

ITIH4, the trypsin inhibitor inter-alpha heavy chain 4 is a type II acute-phase protein (APP) involved in inflammatory responses to trauma and acute ischemia in humans (Kashyap et al. 2009). It is induced by IL6 in hepatocytes and may also play a role in liver development and regeneration. It is located on SSC13 and associated with ear score and wall bleeding. The roles of ischemia (Reiner and Lechner 2019), IL6 (Ringseis et al. 2021) and acute-phase reaction (Löewenstein et al. 2021) in SINS have been demonstrated. The synthesis of acute-phase proteins takes place mainly in the liver under the stimulus of the pro-inflammatory cytokines IL-1β, IL-6 and TNF-α (Petersen et al. 2004).

Reticulon 3 (RTN3) is involved in endoplasmatic reticulum stress, apoptosis and inflammation (Wan et al. 2007). It is a repressor of NFkB and thus a candidate gene in SINS, as all three aspects have been described with the syndrome (Ringseis et al. 2021). It was located on SSC2, associated with inflammation of the ears.

SARM1 is involved in innate immune response in mammals. It is a negative Toll-like receptor regulator and inhibits TICAM1/TRIF- and MYD88-dependent activation of JUN/AP-1, TRIF-dependent activation of NF-kappa-B and IRF3, and the phosphorylation of MAPK14/p38 (Carty et al. 2006). This makes it a further interesting candidate gene for SINS that has a potential to regulate the LPS signal to inflammation which is an important part in SINS pathogenesis (Reiner et al. 2021a). It is located 72 kbp from a SNP on SSC12 associated with SINS and heels bleeding.

ZFAND6 (A20-type zinc finger domain) is involved in the regulation of monocytes and the regulation of TNF-alpha-induced NF-kappa-B activation and apoptosis (Fenner et al. 2009). The according SNP lies on SSC7 and is associated with ear and face score and fits well to the important role of monocytes and apoptosis in SINS (Löewenstein et al. 2021). NUDT3 is also involved in the regulation of the NF-κB signaling pathway (Warner et al. 2013). P4HA1 is involved in collagenogenesis (Annunen et al. 1997). This gene may therefore be involved in the variability of skin and skin appendage sensitivity to inflammation and necrosis.

DOCK2 (Dedicator of cytogenesis domain 2) supports lymphocyte migration in response to chemokines by cytoskeletal rearrangement (Kulkarni et al. 2011). The involvement of chemokins that are involved in lymphocyte migration (ICAM1) have been described (Ringseis et al. 2021).

Some interesting candidate genes are located further apart from the SNPs. One example is CCL2 (distance: 3.9 Mbp from significant SNP) in the region of SSC12, associated with SINS. As a member of the chemokine superfamily of secreted proteins involved in immunoregulatory and inflammatory processes, CCL2 displays chemotactic activity for monocytes and basophils in humans (Weber et al. 1996). An earlier name for CCL2 was monocyte chemoattractant protein-1 (MCP-1). It has been implicated in the pathogenesis of diseases characterized by monocytic infiltrates, like psoriasis, rheumatoid arthritis and atherosclerosis (Zhang et al. 1994). CCL2 seems to be involved in the recruitment of monocytes into the arterial wall during the disease process of human atherosclerosis (Li et al. 1993). This process seems to be a typical finding of SINS (Kühling et al. 2021a, b, Löewenstein et al. 2021, Ringseis et al. 2021). Different gene variants (cis or trans) might lead to higher monocyte recruitment rates followed by higher degrees of SINS. It was shown that porcine CCL2 mechanisms works in the same way than in mouse and human and that porcine monocyte subsets differ in the expression of CCL2 and in their responsiveness to CCL2 (Moreno et al. 2010).

The relatively low number of animals is a significant weakness in this study. The reason lies in the extremely time-consuming recording of phenotypes. Here, future studies could work out possibilities to enable reduced data collection while retaining as much information as possible regarding SINS. Population stratification is one of the major confounding factors in GWAS (Liu et al. 2021, Yan et al. 2022). If case and control samples are drawn disproportionally from different populations and allel frequencies are differing in different populations, an inflation of type 1 error rates can arise (Freedman et al. 2004). This problem increases with the increasing numbers in sample-size in large-scale association studies (Reich and Goldstein 2001; Price et al. 2006; Hellwege et al. 2017; Liu et al. 2021). BLINK was used to incorporate principal components as covariates to reduce false positives due to population stratification and to iteratively incorporate associated markers as covariates to eliminate their connection to the cryptic relationship among individuals (Wang and Zhang 2021). Using principal components is a suitable tool to control populations stratification, but it also leads to negative bias (Bouaziz et al. 2011; Zhao et al 2018). Depending on the LD structure of the data it might happened that a substantial proportion of the genetic variation is removed from the data set, which leads to the increase in the number of false positives and a reduction of the power. Although Principal component analysis was used to control the stratification, there is still a risk regarding false positives and the loss in power.

Because of the relatively low coverage, the relatively low number of individuals with relatively complex scores and the hypothesis that SINS as a syndrome might have evolved as a side effect of complex selection in swine, it could not necessarily be expected to have large effects of major gene character and that all effects could actually be mapped by the present study.

The use of mixed semen also did not only show the expected favourable effects on the environmental control. In about a third of the litters, one of the two boars prevailed particularly clearly, which influenced the distribution of piglets. The reason for this effect remains open, because in some litters boar 1 prevailed over boar 2, in some it was the other way round. As a result, both the number of offspring per boar was largely balanced in the end, and the degrees of relationship were reduced. Furthermore, the distributions were taken into account by considering the litter and the PCA values in the GWAS.

Nevertheless, the postulated genome-wide distribution of effects with association to the various clinical signs of the syndrome was confirmed. In fact, effects explaining more than 10 to 30% of the phenotypic variance also occurred. But the aforementioned aspects of the study meant that no functional SNP could be mapped. Future studies are reserved to determine whether functional SNPs are present in linkage with the mapped SNPs and whether these could be lncRNAs, variation in promoter regions, exon mutations with amino acid exchange, or other genomic variants.

## Conclusion

Swine inflammation and necrosis syndrome can affect a number of body parts with varying degrees of inflammation and necrosis. The inflammatory basis of the syndrome is well characterized and points to a variety of potential genetic factors. The present study is the first to demonstrate the genome-wide association of the syndrome with gene markers. The results suggest a polygenic inheritance. Candidate genes can be defined in the vicinity of numerous SNPs. The identification of the responsible functional polymorphisms is reserved for future studies.

### Supplementary Information

Below is the link to the electronic supplementary material.

Supplementary file1 (DOCX 124 KB)

## Data Availability

The datasets generated and analysed during this study are available from the corresponding author on reasonable request.
