# Supplementary material for: GWAS reveals genomic associations with swine inflammation and necrosis syndrome
Source: Mamm Genome. 2023 Aug 1;34(4):586–601. doi: 10.1007/s00335-023-10011-6 (PMC10627913; doi:10.1007/s00335-023-10011-6)
Supplement: Supplementary file 1 — Supplementary file1 (DOCX 124 KB) [file 335_2023_10011_MOESM1_ESM.docx]

**GWAS reveals genomic associations with Swine Inflammation and Necrosis Syndrome**

**Mammalian Genome**

Katharina Gerhards, Sabrina Becker, Josef Kuehling, Mirjam Lechner, Jochen Bathke, Hermann Willems, Gerald Reiner

Supplemental Tables

**Supplementary Table 1**  Single findings in Suckling piglets by body part and boar. All data in percent. Data were analysed with a General Linear Model considering the effect of the boar.

| **Body part** | **Phenotype** | **Offspring** | **N** | **Mean** | **± SE** | **LCI95** | **UCI95** | **P_boar_** | **R^2^** |
| --- | --- | --- | --- | --- | --- | --- | --- | --- | --- |
| Tail base | No bristles | All | 234 | 35.0 | 3.1 | 28.9 | 41.2 | <0.001 | 10.8 |
|  |  | DU boar^a^ | 48 | 14.6 | 5.1 | 4.2 | 24.9 |  |  |
|  |  | PI+ boar^a^ | 77 | 24.7 | 4.9 | 14.8 | 34.5 |  |  |
|  |  | PI- boar^b^ | 109 | 51.4 | 4.8 | 41.8 | 60.9 |  |  |
|  | Swelling | All | 234 | 42.3 | 3.2 | 35.9 | 48.7 | <0.001 | 11.3 |
|  |  | DU boar^a^ | 48 | 20.8 | 5.9 | 8.9 | 32.8 |  |  |
|  |  | PI+ boar^a^ | 77 | 31.2 | 5.3 | 20.6 | 41.8 |  |  |
|  |  | PI- boar^b^ | 109 | 59.6 | 4.7 | 50.3 | 69.0 |  |  |
|  | Redness | All | 234 | 36.8 | 3.2 | 30.5 | 43.0 | 0.504 | 0.6 |
|  |  | DU boar | 48 | 43.8 | 7.2 | 29.2 | 58.3 |  |  |
|  |  | PI+ boar | 77 | 36.4 | 5.5 | 25.4 | 47.4 |  |  |
|  |  | PI- boar | 109 | 33.9 | 4.6 | 24.9 | 43.0 |  |  |
|  | Exudation | All | 234 | 0.4 | 0.4 | -0.4 | 1.3 | 0.566 | 0.5 |
|  |  | DU boar | 48 | 0.0 | 0.0 | 0.0 | 0.0 |  |  |
|  |  | PI+ boar | 77 | 0.0 | 0.0 | 0.0 | 0.0 |  |  |
|  |  | PI- boar | 109 | 0.9 | 0.9 | -0.9 | 2.7 |  |  |
|  | Necrosis | All | 234 | 0.0 | 0.0 | 0.0 | 0.0 | n.s. | - |
|  |  | DU boar | 48 | 0.0 | 0.0 | 0.0 | 0.0 |  |  |
|  |  | PI+ boar | 77 | 0.0 | 0.0 | 0.0 | 0.0 |  |  |
|  |  | PI- boar | 109 | 0.0 | 0.0 | 0.0 | 0.0 |  |  |
| Tail tip | No bristles | All | 234 | 7.7 | 1.7 | 4.3 | 11.1 | 0.001 | 6.1 |
|  |  | DU boar^a^ | 48 | 0.0 | 0.0 | 0.0 | 0.0 |  |  |
|  |  | PI+ boar^a^ | 77 | 2.6 | 1.8 | -1.0 | 6.2 |  |  |
|  |  | PI- boar^b^ | 109 | 14.7 | 3.4 | 7.9 | 21.4 |  |  |
|  | Swelling | All | 234 | 8.5 | 1.8 | 4.9 | 12.2 | 0.001 | 5.5 |
|  |  | DU boar^a^ | 48 | 2.1 | 2.1 | -2.1 | 6.3 |  |  |
|  |  | PI+ boar^a^ | 77 | 2.6 | 1.8 | -1.0 | 6.2 |  |  |
|  |  | PI- boar^b^ | 109 | 15.6 | 3.5 | 8.7 | 22.5 |  |  |
|  | Scab formation | All | 234 | 28.6 | 3.0 | 22.8 | 34.5 | 0.042 | 2.7 |
|  |  | DU boar^a^ | 48 | 16.7 | 5.4 | 5.7 | 27.6 |  |  |
|  |  | PI+ boar^ab^ | 77 | 26.0 | 5.0 | 16.0 | 36.0 |  |  |
|  |  | PI- boar^b^ | 109 | 35.8 | 4.6 | 26.6 | 44.9 |  |  |
|  | Rhagades | All | 234 | 4.3 | 1.3 | 1.7 | 6.9 | 0.606 | 0.4 |
|  |  | DU boar | 48 | 6.3 | 3.5 | -0.9 | 13.4 |  |  |
|  |  | PI+ boar | 77 | 2.6 | 1.8 | -1.0 | 6.2 |  |  |
|  |  | PI- boar | 109 | 4.6 | 2.0 | 0.6 | 8.6 |  |  |
|  | Exudation | All | 234 | 15.0 | 2.3 | 10.4 | 19.6 | 0.605 | 0.4 |
|  |  | DU boar | 48 | 14.6 | 5.1 | 4.2 | 24.9 |  |  |
|  |  | PI+ boar | 77 | 18.2 | 4.4 | 9.4 | 27.0 |  |  |
|  |  | PI- boar | 109 | 12.8 | 3.2 | 6.5 | 19.2 |  |  |
|  | Necrosis | All | 234 | 3.8 | 1.3 | 1.4 | 6.3 | 0.123 | 1.8 |
|  |  | DU boar^a^ | 48 | 0.0 | 0.0 | 0.0 | 0.0 |  |  |
|  |  | PI+ boar^ab^ | 77 | 2.6 | 1.8 | -1.0 | 6.2 |  |  |
|  |  | PI- boar^b^ | 109 | 6.4 | 2.4 | 1.7 | 11.1 |  |  |
|  | Ring-shaped constrictions | All | 234 | 0.4 | 0.4 | -0.4 | 1.3 | 0.566 | 0.5 |
|  |  | DU boar | 48 | 0.0 | 0.0 | 0.0 | 0.0 |  |  |
|  |  | PI+ boar | 77 | 0.0 | 0.0 | 0.0 | 0.0 |  |  |
|  |  | PI- boar | 109 | 0.9 | 0.9 | -0.9 | 2.7 |  |  |
|  | Bleeding | All | 234 | 1.3 | 0.7 | -0.2 | 2.7 | 0.838 | 0.2 |
|  |  | DU boar | 48 | 2.1 | 2.1 | -2.1 | 6.3 |  |  |
|  |  | PI+ boar | 77 | 1.3 | 1.3 | -1.3 | 3.9 |  |  |
|  |  | PI- boar | 109 | 0.9 | 0.9 | -0.9 | 2.7 |  |  |

| **Body part** | **Phenotype** | **Offspring** | **N** | **Mean** | **± SE** | **LCI95** | **UCI95** | **P_boar_** | **R^2^** |
| --- | --- | --- | --- | --- | --- | --- | --- | --- | --- |
| Ears | Shiny skin | All | 234 | 74.8 | 2.8 | 69.2 | 80.4 | <0.001 | 10.4 |
|  |  | DU boar^a^ | 48 | 47.9 | 7.3 | 33.3 | 62.6 |  |  |
|  |  | PI+ boar^b^ | 77 | 85.7 | 4.0 | 77.7 | 93.7 |  |  |
|  |  | PI- boar^b^ | 109 | 78.9 | 3.9 | 71.1 | 86.7 |  |  |
|  | No bristles | All | 234 | 65.8 | 3.1 | 59.7 | 71.9 | <0.001 | 12.2 |
|  |  | DU boar^a^ | 48 | 33.3 | 6.9 | 19.5 | 47.2 |  |  |
|  |  | PI+ boar^b^ | 77 | 76.6 | 4.9 | 67.0 | 86.3 |  |  |
|  |  | PI- boar^b^ | 109 | 72.5 | 4.3 | 64.0 | 81.0 |  |  |
|  | Venous congestion | All | 234 | 86.3 | 2.3 | 81.9 | 90.8 | <0.001 | 6.8 |
|  |  | DU boar^a^ | 48 | 68.8 | 6.8 | 55.1 | 82.4 |  |  |
|  |  | PI+ boar^b^ | 77 | 90.9 | 3.3 | 84.3 | 97.5 |  |  |
|  |  | PI- boar^b^ | 109 | 90.8 | 2.8 | 85.3 | 96.3 |  |  |
| Face (edema) | Eye lids | All | 230 | 95.2 | 1.4 | 92.4 | 98.0 | 0.845 | 0.1 |
|  |  | DU boar | 47 | 95.7 | 3.0 | 89.8 | 101.7 |  |  |
|  |  | PI+ boar | 77 | 96.1 | 2.2 | 91.7 | 100.5 |  |  |
|  |  | PI- boar | 106 | 94.3 | 2.3 | 89.9 | 98.8 |  |  |
|  | Nose back | All | 230 | 7.8 | 1.8 | 4.3 | 11.3 | 0.313 | 1.0 |
|  |  | DU boar | 47 | 12.8 | 4.9 | 2.9 | 22.7 |  |  |
|  |  | PI+ boar | 77 | 5.2 | 2.5 | 0.1 | 10.3 |  |  |
|  |  | PI- boar | 106 | 7.5 | 2.6 | 2.4 | 12.7 |  |  |
| Navel | Inflammation | All | 234 | 16.2 | 2.4 | 11.5 | 21.0 | 0.718 | 0.3 |
|  |  | DU boar | 48 | 14.6 | 5.1 | 4.2 | 24.9 |  |  |
|  |  | PI+ boar | 77 | 14.3 | 4.0 | 6.3 | 22.3 |  |  |
|  |  | PI- boar | 109 | 18.3 | 3.7 | 11.0 | 25.7 |  |  |
| Teats | Swelling | All | 234 | 21.8 | 2.7 | 16.5 | 27.1 | 0.027 | 3.1 |
|  |  | DU boar^a^ | 48 | 12.5 | 4.8 | 2.8 | 22.2 |  |  |
|  |  | PI+ boar^ab^ | 77 | 16.9 | 4.3 | 8.3 | 25.4 |  |  |
|  |  | PI- boar^b^ | 109 | 29.4 | 4.4 | 20.7 | 38.0 |  |  |
|  | Redness | All | 234 | 1.7 | 0.8 | 0.0 | 3.4 | 0.550 | 0.5 |
|  |  | DU boar | 48 | 0.0 | 0.0 | 0.0 | 0.0 |  |  |
|  |  | PI+ boar | 77 | 2.6 | 1.8 | -1.0 | 6.2 |  |  |
|  |  | PI- boar | 109 | 1.8 | 1.3 | -0.7 | 4.4 |  |  |
|  | Scab formation | All | 234 | 1.7 | 0.8 | 0.0 | 3.4 | 0.939 | 0.1 |
|  |  | DU boar | 48 | 2.1 | 2.1 | -2.1 | 6.3 |  |  |
|  |  | PI+ boar | 77 | 1.3 | 1.3 | -1.3 | 3.9 |  |  |
|  |  | PI- boar | 109 | 1.8 | 1.3 | -0.7 | 4.4 |  |  |
|  | Necrosis | All | 234 | 7.7 | 1.7 | 4.3 | 11.1 | 0.731 | 0.3 |
|  |  | DU boar | 48 | 6.3 | 3.5 | -0.9 | 13.4 |  |  |
|  |  | PI+ boar | 77 | 6.5 | 2.8 | 0.9 | 12.1 |  |  |
|  |  | PI- boar | 109 | 9.2 | 2.8 | 3.7 | 14.7 |  |  |
|  | Venous congestion | All | 234 | 44.9 | 3.3 | 38.5 | 51.3 | <0.001 | 11.5 |
|  |  | DU boar^a^ | 48 | 12.5 | 4.8 | 2.8 | 22.2 |  |  |
|  |  | PI+ boar^b^ | 77 | 58.4 | 5.7 | 47.2 | 69.7 |  |  |
|  |  | PI- boar^b^ | 109 | 49.5 | 4.8 | 40.0 | 59.1 |  |  |
| Coronary bands (inflammation | Hind limb | All | 231 | 58.4 | 3.2 | 52.0 | 64.8 | 0.014 | 3.7 |
|  |  | DU boar^a^ | 47 | 51.1 | 7.4 | 36.2 | 65.9 |  |  |
|  |  | PI+ boar^a^ | 76 | 48.7 | 5.8 | 37.2 | 60.2 |  |  |
|  |  | PI- boar^b^ | 108 | 68.5 | 4.5 | 59.6 | 77.4 |  |  |
|  | Front limb | All | 233 | 48.5 | 3.3 | 42.0 | 55.0 | 0.010 | 3.9 |
|  |  | DU boar^ab^ | 48 | 43.8 | 7.2 | 29.2 | 58.3 |  |  |
|  |  | PI+ boar^a^ | 76 | 36.8 | 5.6 | 25.7 | 47.9 |  |  |
|  |  | PI- boar^b^ | 109 | 58.7 | 4.7 | 49.3 | 68.1 |  |  |

| **Body part** | **Phenotype** | **Offspring** | **N** | **Mean** | **± SE** | **LCI95** | **UCI95** | **P_boar_** | **R^2^** |
| --- | --- | --- | --- | --- | --- | --- | --- | --- | --- |
| Wall | Bleeding (hind) | All | 231 | 86.6 | 2.2 | 82.2 | 91.0 | 0.161 | 1.6 |
|  |  | DU boar^a^ | 47 | 78.7 | 6.0 | 66.6 | 90.9 |  |  |
|  |  | PI+ boar^b^ | 76 | 90.8 | 3.3 | 84.1 | 97.4 |  |  |
|  |  | PI- boar^ab^ | 108 | 87.0 | 3.2 | 80.6 | 93.5 |  |  |
|  | Bleeding (front) | All | 233 | 85.8 | 2.3 | 81.3 | 90.3 | 0.282 | 1.1 |
|  |  | DU boar | 48 | 81.3 | 5.7 | 69.8 | 92.7 |  |  |
|  |  | PI+ boar | 76 | 90.8 | 3.3 | 84.1 | 97.4 |  |  |
|  |  | PI- boar | 109 | 84.4 | 3.5 | 77.5 | 91.3 |  |  |
| Heels | Swelling (hind) | All | 234 | 97.4 | 1.0 | 95.4 | 99.5 | <0.001 | 6.4 |
|  |  | DU boar^a^ | 48 | 89.6 | 4.5 | 80.6 | 98.5 |  |  |
|  |  | PI+ boar^b^ | 77 | 100.0 | 0.0 | 100.0 | 100.0 |  |  |
|  |  | PI- boar^b^ | 109 | 99.1 | 0.9 | 97.3 | 100.9 |  |  |
|  | Swelling (front) | All | 233 | 97.4 | 1.0 | 95.4 | 99.5 | 0.013 | 3.7 |
|  |  | DU boar^a^ | 48 | 91.7 | 4.0 | 83.6 | 99.8 |  |  |
|  |  | PI+ boar^b^ | 76 | 100.0 | 0.0 | 100.0 | 100.0 |  |  |
|  |  | PI- boar^b^ | 109 | 98.2 | 1.3 | 95.6 | 100.7 |  |  |
|  | Bleeding (hind) | All | 234 | 94.4 | 1.5 | 91.5 | 97.4 | 0.635 | 0.4 |
|  |  | DU boar | 48 | 91.7 | 4.0 | 83.6 | 99.8 |  |  |
|  |  | PI+ boar | 77 | 94.8 | 2.5 | 89.7 | 99.9 |  |  |
|  |  | PI- boar | 109 | 95.4 | 2.0 | 91.4 | 99.4 |  |  |
|  | Bleeding (front) | All | 233 | 97.4 | 1.0 | 95.4 | 99.5 | 0.018 | 3.4 |
|  |  | DU boar^a^ | 48 | 91.7 | 4.0 | 83.6 | 99.8 |  |  |
|  |  | PI+ boar^b^ | 76 | 98.7 | 1.3 | 96.1 | 101.3 |  |  |
|  |  | PI- boar^b^ | 109 | 99.1 | 0.9 | 97.3 | 100.9 |  |  |

a, b, c: Offspring groups with different letters are statistically significantly different at P <= 0.05. P_Boar_: Significance of boar effect. R^2^: Coefficient of determination.

**Supplemental Table 2**  Significant SNPs with position, significance level and associated SINS sign

| SNP_ID | SSC | Position | P | MAF | N | FDR | negLog10(P) | Level of P | Sign | REF | ALT |
| --- | --- | --- | --- | --- | --- | --- | --- | --- | --- | --- | --- |
| rs342873320 | 1 | 4508329 | 1.56E-10 | 0.38 | 225 | 1.60E-04 | 9.8 | genome | SINS | A | T |
| rs319332000 | 1 | 14157691 | 7.68E-11 | 0.09 | 234 | 5.26E-05 | 10.1 | genome | Tail swelling | C | A |
| rs321888252 | 1 | 16224794 | 2.23E-08 | 0.06 | 234 | 4.57E-02 | 7.7 | chromosome | Tail exudate | A | G |
| rs326246725 | 1 | 19763293 | 8.35E-12 | 0.07 | 234 | 1.24E-05 | 11.1 | genome | Teat necrosis | C | T |
| rs340236852 | 1 | 31190769 | 6.61E-12 | 0.15 | 234 | 1.36E-05 | 11.2 | genome | Tail swelling | C | A |
| rs707149464 | 1 | 35812331 | 8.20E-10 | 0.05 | 234 | 1.68E-03 | 9.1 | genome | Tail tip score | A | C |
| rs3470810184 | 1 | 47534745 | 2.58E-09 | 0.06 | 234 | 2.65E-03 | 8.6 | genome | Ears no bristles | C | A |
| rs333260188 | 1 | 56360947 | 7.68E-09 | 0.14 | 234 | 5.26E-03 | 8.1 | chromosome | Ears vein congestion | C | T |
| rs337978801 | 1 | 66905225 | 1.21E-08 | 0.16 | 230 | 8.27E-03 | 7.9 | chromosome | Wall bleeding hind | G | A |
| rs320250964 | 1 | 75609421 | 8.90E-10 | 0.06 | 234 | 4.57E-04 | 9.1 | genome | Tail swelling | T | C |
| rs1112134158 | 1 | 212887602 | 5.20E-15 | 0.15 | 225 | 1.07E-08 | 14.3 | genome | SINS | A | C |
| rs3472729085 | 1 | 224713985 | 1.20E-11 | 0.21 | 234 | 1.24E-05 | 10.9 | genome | Teat necrosis | T | C |
| rs319104772 | 1 | 243296440 | 7.58E-09 | 0.43 | 234 | 5.19E-03 | 8.1 | chromosome | Ear score | A | G |
| rs327474318 | 1 | 253523847 | 1.33E-09 | 0.20 | 234 | 9.13E-04 | 8.9 | genome | Teat necrosis | T | C |
| rs337681369 | 1 | 271201482 | 3.85E-11 | 0.18 | 234 | 7.92E-05 | 10.4 | genome | Tail base score | G | A |
| rs329833250 | 1 | 271968856 | 3.94E-12 | 0.28 | 234 | 4.05E-06 | 11.4 | genome | Ear score | G | A |
| rs344431745 | 1 | 273401709 | 7.42E-13 | 0.12 | 233 | 1.53E-06 | 12.1 | genome | Coronary band inflammation front | C | T |
| rs331316020 | 1 | 274101520 | 5.92E-12 | 0.39 | 233 | 1.22E-05 | 11.2 | genome | Wall bleeding front | C | A |
| rs339107366 | 2 | 8270586 | 4.96E-11 | 0.06 | 234 | 1.02E-04 | 10.3 | genome | Ears vein congestion | G | A |
| rs3470030364 | 2 | 13484586 | 7.16E-13 | 0.30 | 234 | 1.47E-06 | 12.1 | genome | Ear score | G | T |
| rs334848645 | 2 | 15794455 | 4.62E-08 | 0.21 | 229 | 4.74E-02 | 7.3 | chromosome | Wall score | T | C |
| rs329718710 | 2 | 23751648 | 5.55E-11 | 0.13 | 234 | 5.26E-05 | 10.3 | genome | Tail swelling | G | A |
| rs340576798 | 2 | 39322886 | 2.29E-10 | 0.07 | 230 | 2.35E-04 | 9.6 | genome | Wall bleeding hind | C | T |
| rs3470445636 | 2 | 39324482 | 1.53E-08 | 0.07 | 229 | 3.14E-02 | 7.8 | chromosome | Wall score | T | C |
| rs342594533 | 2 | 86288691 | 2.45E-08 | 0.15 | 234 | 1.26E-02 | 7.6 | chromosome | Ear score | T | C |
| rs330531331 | 2 | 104384195 | 5.28E-11 | 0.23 | 234 | 1.08E-04 | 10.3 | genome | Ears no bristles | G | A |
| rs327196951 | 2 | 118205756 | 6.48E-09 | 0.27 | 234 | 5.26E-03 | 8.2 | chromosome | Ears vein congestion | C | T |
| rs337366176 | 2 | 140645128 | 1.81E-11 | 0.46 | 230 | 3.72E-05 | 10.7 | genome | Wall bleeding hind | T | C |
| rs336841690 | 3 | 1988881 | 5.20E-09 | 0.36 | 234 | 8.87E-03 | 8.3 | chromosome | Tail base score | C | T |
| rs330814531 | 3 | 16013886 | 3.00E-12 | 0.38 | 234 | 7.15E-06 | 11.5 | genome | Tail scabs | G | T |
| 3_22988098 | 3 | 22988098 | 5.34E-10 | 0.29 | 233 | 1.27E-03 | 9.3 | genome | Wall bleeding front |  |  |
| rs325504291 | 3 | 22988098 | 6.48E-09 | 0.29 | 229 | 1.54E-02 | 8.2 | chromosome | Wall score | G | A |
| rs322856282 | 3 | 30052974 | 1.15E-08 | 0.17 | 225 | 9.09E-03 | 7.9 | chromosome | SINS | A | G |
| 3_30546515 | 3 | 30546515 | 5.59E-14 | 0.05 | 230 | 1.33E-07 | 13.3 | genome | Nose back edema |  |  |
| rs322403572 | 3 | 53445482 | 4.76E-11 | 0.06 | 234 | 5.67E-05 | 10.3 | genome | Tail scabs | T | C |
| rs3472170549 | 3 | 55155672 | 7.46E-09 | 0.41 | 234 | 8.87E-03 | 8.1 | chromosome | Tail base score | G | C |
| rs1110025725 | 3 | 55171658 | 5.25E-11 | 0.44 | 234 | 1.25E-04 | 10.3 | genome | Tail base no bristles | A | G |
| rs344364402 | 3 | 60845061 | 1.90E-12 | 0.14 | 229 | 4.53E-06 | 11.7 | genome | Coronary band score | A | G |
| rs345937656 | 3 | 64131673 | 2.08E-08 | 0.14 | 230 | 4.95E-02 | 7.7 | chromosome | Eye edema | C | T |
| rs323112370 | 3 | 75265156 | 2.64E-08 | 0.45 | 234 | 2.10E-02 | 7.6 | chromosome | Ears vein congestion | C | T |
| rs697469710 | 3 | 101050773 | 1.13E-08 | 0.09 | 234 | 2.68E-02 | 7.9 | chromosome | Tail swelling | C | A |
| rs3473008952 | 3 | 124855843 | 6.46E-10 | 0.15 | 234 | 7.69E-04 | 9.2 | genome | Ears vein congestion | G | A |
| rs329334728 | 4 | 2511700 | 5.18E-10 | 0.13 | 230 | 6.17E-04 | 9.3 | genome | Wall bleeding hind | C | T |
| rs330403525 | 4 | 4813837 | 6.15E-10 | 0.09 | 225 | 7.32E-04 | 9.2 | genome | SINS | T | C |
| rs336133151 | 4 | 11059334 | 1.06E-08 | 0.49 | 229 | 1.27E-02 | 8 | chromosome | Coronary band score | A | G |
| rs339755081 | 4 | 15775626 | 1.69E-09 | 0.30 | 234 | 2.01E-03 | 8.8 | genome | Ear score | T | C |
| rs337370032 | 4 | 72908203 | 6.81E-11 | 0.19 | 234 | 1.62E-04 | 10.2 | genome | Tail base redness | G | T |
| rs346157884 | 4 | 89152946 | 2.45E-09 | 0.06 | 234 | 5.82E-03 | 8.6 | genome | Ears shiny skin | A | C |
| rs344992278 | 4 | 91282935 | 1.52E-16 | 0.08 | 225 | 3.62E-10 | 15.8 | genome | SINS | T | C |
| rs702001148 | 4 | 106109743 | 1.75E-09 | 0.14 | 230 | 2.08E-03 | 8.8 | genome | Nose back edema | G | A |
| rs787532906 | 5 | 46569482 | 6.56E-11 | 0.06 | 234 | 1.56E-04 | 10.2 | genome | Ears vein congestion | C | T |
| rs1109404276 | 5 | 66261808 | 2.58E-08 | 0.41 | 234 | 6.14E-02 | 7.6 | chromosome | Ears no bristles | G | T |
| rs325386142 | 5 | 68033728 | 7.59E-11 | 0.25 | 233 | 9.03E-05 | 10.1 | genome | Heels score | A | G |
| rs696848580 | 5 | 74130702 | 6.05E-11 | 0.07 | 234 | 1.44E-04 | 10.2 | genome | Teat swelling | G | A |
| rs707595743 | 5 | 80628823 | 1.95E-12 | 0.32 | 234 | 4.64E-06 | 11.7 | genome | Heels Bleeding hind | C | T |
| rs333396853 | 5 | 81674515 | 6.67E-12 | 0.16 | 230 | 1.59E-05 | 11.2 | genome | Wall bleeding hind | G | A |
| rs345547323 | 5 | 83317374 | 5.14E-12 | 0.14 | 233 | 1.22E-05 | 11.3 | genome | Heels score | T | G |
| rs343731406 | 5 | 85068157 | 2.81E-08 | 0.15 | 230 | 6.68E-02 | 7.6 | chromosome | Face score | C | T |
| rs345598409 | 5 | 86636102 | 4.66E-11 | 0.08 | 234 | 1.11E-04 | 10.3 | genome | Ear score | C | A |
| rs325751412 | 5 | 100172331 | 2.07E-09 | 0.06 | 234 | 2.46E-03 | 8.7 | genome | Heels Bleeding hind | G | T |
| rs327642865 | 6 | 10345325 | 2.31E-08 | 0.17 | 230 | 2.05E-02 | 7.6 | chromosome | Wall bleeding hind | A | G |
| rs324472847 | 6 | 18668130 | 5.30E-11 | 0.16 | 230 | 1.70E-04 | 10.3 | genome | Face score | A | G |
| rs3470068869 | 6 | 43583063 | 2.41E-11 | 0.11 | 233 | 8.54E-05 | 10.6 | genome | Coronary band inflammation front | A | C |
| rs707228272 | 6 | 45590628 | 3.47E-14 | 0.24 | 229 | 1.23E-07 | 13.5 | genome | Coronary band score | C | T |
| rs694593359 | 6 | 87438411 | 1.10E-14 | 0.11 | 234 | 1.95E-08 | 14 | genome | Tail swelling | A | G |
| 6_126898383 | 6 | 126898383 | 2.76E-10 | 0.06 | 234 | 3.26E-04 | 9.6 | genome | Ears shiny skin |  |  |
| rs1108119965 | 6 | 127841510 | 1.55E-10 | 0.14 | 230 | 1.09E-04 | 9.8 | genome | Nose back edema | G | A |
| rs343846785 | 6 | 152291242 | 3.72E-13 | 0.16 | 234 | 1.32E-06 | 12.4 | genome | Heels Bleeding hind | T | G |
| rs340950675 | 6 | 153984413 | 1.48E-09 | 0.07 | 234 | 1.05E-03 | 8.8 | genome | Tail swelling | A | G |
| rs320586244 | 7 | 6378335 | 8.28E-11 | 0.36 | 234 | 2.93E-04 | 10.1 | genome | Tail base no bristles | T | C |
| rs1108090519 | 7 | 7831022 | 9.05E-14 | 0.22 | 234 | 3.21E-07 | 13 | genome | Ears shiny skin | T | G |
| rs80971262 | 7 | 9776278 | 3.65E-11 | 0.24 | 234 | 1.30E-04 | 10.4 | genome | Ear score | C | T |
| rs690595812 | 7 | 21333865 | 3.46E-10 | 0.07 | 234 | 1.23E-03 | 9.5 | genome | Tail scabs | G | A |
| rs3471905095 | 7 | 22949346 | 8.42E-11 | 0.05 | 234 | 7.46E-05 | 10.1 | genome | Tail swelling | C | T |
| rs338509948 | 7 | 26025361 | 5.09E-17 | 0.10 | 234 | 1.81E-10 | 16.3 | genome | Tail swelling | C | G |
| rs3472975616 | 7 | 37255799 | 3.88E-09 | 0.06 | 234 | 6.87E-03 | 8.4 | chromosome | Tail scabs | C | G |
| rs321766265 | 7 | 43205907 | 2.20E-08 | 0.28 | 234 | 3.91E-02 | 7.7 | chromosome | Tail base no bristles | A | C |
| rs330979347 | 7 | 49086851 | 9.58E-11 | 0.13 | 230 | 1.70E-04 | 10 | genome | Face score | A | G |
| rs329474420 | 7 | 96238503 | 5.83E-11 | 0.12 | 233 | 1.03E-04 | 10.2 | genome | Wall bleeding front | A | C |
| rs690809420 | 7 | 97946588 | 1.55E-08 | 0.34 | 234 | 9.18E-03 | 7.8 | chromosome | Tail swelling | T | A |
| rs334430515 | 8 | 1208125 | 4.89E-10 | 0.05 | 230 | 8.67E-04 | 9.3 | genome | Wall bleeding hind | G | A |
| rs319241944 | 8 | 72440718 | 2.16E-10 | 0.15 | 230 | 1.09E-04 | 9.7 | genome | Nose back edema | G | A |
| rs343636853 | 8 | 127385592 | 5.64E-10 | 0.11 | 234 | 9.99E-04 | 9.2 | genome | Ear score | A | G |
| rs1110251978 | 8 | 131758708 | 6.79E-09 | 0.22 | 229 | 1.20E-02 | 8.2 | chromosome | Coronary band score | G | A |
| rs322919962 | 8 | 136683047 | 2.34E-12 | 0.13 | 230 | 4.15E-06 | 11.6 | genome | Nose back edema | G | A |
| rs3473385413 | 8 | 137928158 | 2.96E-11 | 0.42 | 230 | 3.50E-05 | 10.5 | genome | Nose back edema | T | C |
| rs323072014 | 8 | 138016195 | 3.95E-14 | 0.10 | 230 | 1.40E-07 | 13.4 | genome | Nose back edema | C | G |
| rs3471021281 | 9 | 2974915 | 1.95E-08 | 0.13 | 230 | 2.05E-02 | 7.7 | chromosome | Wall bleeding hind | A | G |
| rs331434680 | 9 | 5432233 | 1.19E-10 | 0.25 | 233 | 2.11E-04 | 9.9 | genome | Coronary band inflammation front | G | T |
| rs327654839 | 9 | 5511145 | 1.68E-14 | 0.14 | 230 | 5.95E-08 | 13.8 | genome | Wall bleeding hind | A | T |
| rs691783128 | 9 | 13753130 | 5.76E-10 | 0.07 | 233 | 6.80E-04 | 9.2 | genome | Wall bleeding front | C | A |
| rs334770866 | 9 | 17634045 | 1.45E-10 | 0.10 | 230 | 1.09E-04 | 9.8 | genome | Nose back edema | T | C |
| rs338652172 | 9 | 20779267 | 6.14E-13 | 0.12 | 234 | 1.09E-06 | 12.2 | genome | Ears shiny skin | C | T |
| rs345143905 | 9 | 40724411 | 2.04E-11 | 0.34 | 233 | 7.25E-05 | 10.7 | genome | Wall bleeding front | C | T |
| rs333761367 | 9 | 70120548 | 1.90E-10 | 0.17 | 230 | 1.09E-04 | 9.7 | genome | Nose back edema | G | C |
| rs341512035 | 9 | 90241577 | 8.26E-09 | 0.15 | 233 | 7.32E-03 | 8.1 | chromosome | Wall bleeding front | C | T |
| rs345982251 | 9 | 137464138 | 1.94E-11 | 0.08 | 234 | 2.29E-05 | 10.7 | genome | Tail swelling | G | T |
| rs1112005127 | 10 | 4367731 | 4.90E-11 | 0.05 | 229 | 8.74E-05 | 10.3 | genome | Wall score | G | A |
| rs319202275 | 10 | 8414213 | 3.79E-15 | 0.13 | 234 | 6.75E-09 | 14.4 | genome | Tail exudate | T | A |
| rs329796204 | 10 | 20062150 | 5.47E-10 | 0.06 | 229 | 4.88E-04 | 9.3 | genome | Coronary band score | A | T |
| rs343748570 | 10 | 21027752 | 1.43E-09 | 0.07 | 230 | 1.14E-03 | 8.8 | genome | Nose back edema | G | T |
| rs1110473419 | 10 | 30282321 | 2.01E-17 | 0.15 | 234 | 3.59E-11 | 16.7 | genome | Tail tip score | C | T |
| rs343219622 | 10 | 35048601 | 1.49E-09 | 0.05 | 230 | 1.14E-03 | 8.8 | genome | Nose back edema | T | A |
| rs336009072 | 10 | 36728786 | 2.90E-08 | 0.14 | 234 | 1.55E-02 | 7.5 | chromosome | Tail tip score | T | A |
| rs701606975 | 10 | 38611132 | 3.49E-08 | 0.06 | 234 | 1.55E-02 | 7.5 | chromosome | Tail tip score | C | G |
| rs701836987 | 10 | 49609959 | 6.70E-13 | 0.14 | 230 | 1.20E-06 | 12.2 | genome | Wall bleeding hind | T | C |
| rs338813550 | 10 | 49785901 | 3.73E-08 | 0.13 | 230 | 1.33E-02 | 7.4 | chromosome | Wall bleeding hind | A | G |
| rs338071751 | 11 | 16097396 | 8.32E-09 | 0.07 | 234 | 4.95E-03 | 8.1 | chromosome | Ears shiny skin | A | G |
| rs787551972 | 11 | 18448156 | 2.42E-08 | 0.29 | 234 | 1.08E-02 | 7.6 | chromosome | Ear score | T | A |
| rs691071989 | 11 | 21365824 | 2.56E-11 | 0.10 | 233 | 4.57E-05 | 10.6 | genome | Coronary band inflammation front | C | A |
| rs3473630143 | 11 | 21453639 | 7.77E-12 | 0.12 | 229 | 1.39E-05 | 11.1 | genome | Coronary band score | C | T |
| rs3473317911 | 11 | 24197536 | 3.93E-09 | 0.31 | 234 | 7.01E-03 | 8.4 | chromosome | Tail base score | T | C |
| rs326029081 | 11 | 70939109 | 2.43E-09 | 0.14 | 230 | 2.17E-03 | 8.6 | genome | Wall bleeding hind | C | T |
| rs3475903338 | 11 | 71239679 | 1.17E-09 | 0.13 | 234 | 1.05E-03 | 8.9 | genome | Ears shiny skin | T | C |
| rs319083466 | 11 | 72467167 | 3.67E-10 | 0.47 | 234 | 6.54E-04 | 9.4 | genome | Heels Bleeding hind | T | C |
| rs788007661 | 11 | 74707488 | 1.98E-11 | 0.22 | 234 | 1.77E-05 | 10.7 | genome | Tail tip score | A | G |
| rs330798787 | 12 | 10426293 | 1.59E-10 | 0.42 | 234 | 2.83E-04 | 9.8 | genome | Tail base redness | G | A |
| rs3473452848 | 12 | 14407795 | 2.70E-08 | 0.13 | 230 | 1.20E-02 | 7.6 | chromosome | Wall bleeding hind | G | A |
| rs3472836618 | 12 | 14498120 | 1.69E-08 | 0.13 | 230 | 1.01E-02 | 7.8 | chromosome | Wall bleeding hind | G | T |
| rs334574118 | 12 | 15058247 | 6.63E-10 | 0.37 | 233 | 5.91E-04 | 9.2 | genome | Coronary band inflammation front | C | T |
| rs693371806 | 12 | 15265438 | 1.91E-09 | 0.08 | 230 | 1.14E-03 | 8.7 | genome | Nose back edema | G | A |
| rs325406143 | 12 | 16344077 | 4.48E-11 | 0.28 | 234 | 7.99E-05 | 10.3 | genome | Ear score | C | T |
| rs320627295 | 12 | 17689507 | 8.37E-16 | 0.07 | 234 | 1.49E-09 | 15.1 | genome | Ears shiny skin | C | T |
| rs3471894350 | 12 | 34198370 | 1.45E-08 | 0.38 | 234 | 2.59E-02 | 7.8 | chromosome | Tail scabs | T | C |
| rs326005134 | 12 | 39668035 | 3.00E-08 | 0.13 | 233 | 5.34E-02 | 7.5 | chromosome | Wall bleeding front | G | A |
| rs705048853 | 12 | 40475317 | 5.23E-09 | 0.09 | 230 | 2.33E-03 | 8.3 | chromosome | Nose back edema | A | G |
| rs1112423847 | 12 | 44738423 | 4.01E-09 | 0.46 | 234 | 3.57E-03 | 8.4 | chromosome | Heels Bleeding hind | T | C |
| rs339057889 | 12 | 50607044 | 1.79E-08 | 0.32 | 234 | 1.07E-02 | 7.7 | chromosome | Ear score | G | T |
| rs341558878 | 12 | 54902440 | 2.69E-09 | 0.21 | 234 | 2.39E-03 | 8.6 | genome | Ear score | G | A |
| rs1107742844 | 13 | 4986899 | 1.52E-16 | 0.16 | 225 | 3.90E-10 | 15.8 | genome | SINS | A | G |
| rs323634102 | 13 | 6150834 | 5.52E-10 | 0.13 | 230 | 2.02E-04 | 9.3 | genome | Nose back edema | A | G |
| rs326432252 | 13 | 9300436 | 1.22E-18 | 0.05 | 230 | 3.14E-12 | 17.9 | genome | Wall bleeding hind | G | C |
| rs338318401 | 13 | 13362832 | 8.76E-11 | 0.19 | 234 | 7.50E-05 | 10.1 | genome | Teat necrosis | T | C |
| rs3475055648 | 13 | 20840635 | 2.76E-16 | 0.06 | 230 | 2.36E-10 | 15.6 | genome | Nose back edema | G | A |
| rs322867298 | 13 | 24140025 | 2.43E-15 | 0.13 | 234 | 6.23E-09 | 14.6 | genome | Ears no bristles | G | A |
| rs342274535 | 13 | 25884213 | 2.17E-10 | 0.05 | 234 | 9.27E-05 | 9.7 | genome | Tail swelling | A | C |
| rs324960842 | 13 | 31441960 | 2.69E-23 | 0.05 | 234 | 2.31E-17 | 22.6 | genome | Tail swelling | A | G |
| rs338636090 | 13 | 34502858 | 1.64E-10 | 0.10 | 234 | 2.10E-04 | 9.8 | genome | Ear score | C | A |
| rs337373577 | 13 | 34980722 | 6.36E-12 | 0.06 | 230 | 3.26E-06 | 11.2 | genome | Wall bleeding hind | C | T |
| rs1109234151 | 13 | 42212034 | 1.38E-12 | 0.07 | 233 | 3.54E-06 | 11.9 | genome | Coronary band inflammation front | G | GT |
| rs3471825821 | 13 | 43345670 | 2.70E-10 | 0.26 | 233 | 1.39E-04 | 9.6 | genome | Wall bleeding front | C | T |
| rs326265377 | 13 | 48553790 | 1.11E-08 | 0.07 | 234 | 7.11E-03 | 8 | chromosome | Teat necrosis | A | T |
| rs345003493 | 13 | 50633738 | 2.88E-19 | 0.09 | 234 | 7.38E-13 | 18.5 | genome | Teat necrosis | T | G |
| rs320518959 | 13 | 53856072 | 1.60E-08 | 0.44 | 234 | 4.09E-02 | 7.8 | chromosome | Tail base score | C | T |
| rs3476305457 | 13 | 56534476 | 1.09E-08 | 0.05 | 230 | 3.98E-03 | 8 | chromosome | Wall bleeding hind | C | G |
| rs3469769005 | 13 | 125703968 | 2.76E-11 | 0.08 | 234 | 3.54E-05 | 10.6 | genome | Tail exudate | A | G |
| rs340238661 | 13 | 130690390 | 1.74E-25 | 0.05 | 234 | 4.45E-19 | 24.8 | genome | Tail swelling | T | C |
| rs321570558 | 13 | 131787576 | 1.13E-09 | 0.05 | 234 | 4.13E-04 | 8.9 | genome | Tail swelling | T | A |
| rs327606603 | 13 | 144290398 | 1.47E-08 | 0.14 | 234 | 4.71E-03 | 7.8 | chromosome | Tail swelling | A | G |
| rs332086972 | 13 | 147948513 | 1.59E-11 | 0.15 | 233 | 2.04E-05 | 10.8 | genome | Heels score | T | C |
| rs318421720 | 13 | 148170559 | 1.11E-14 | 0.43 | 234 | 2.86E-08 | 14 | genome | Tail base redness | T | C |
| rs703734816 | 13 | 192132372 | 1.33E-12 | 0.06 | 234 | 3.42E-06 | 11.9 | genome | Tail exudate | C | G |
| rs332789869 | 13 | 193355481 | 3.24E-09 | 0.06 | 234 | 4.15E-03 | 8.5 | genome | Ears shiny skin | A | T |
| rs338331350 | 13 | 204539983 | 2.42E-24 | 0.07 | 230 | 6.21E-18 | 23.6 | genome | Nose back edema | G | A |
| rs712614263 | 13 | 205064145 | 1.26E-11 | 0.09 | 230 | 8.05E-06 | 10.9 | genome | Nose back edema | T | C |
| rs3475049353 | 13 | 208309290 | 1.26E-13 | 0.07 | 233 | 1.08E-07 | 12.9 | genome | Wall bleeding front | G | A |
| rs335756388 | 14 | 556732 | 4.09E-20 | 0.13 | 233 | 1.05E-13 | 19.4 | genome | Wall bleeding front | G | A |
| rs339983250 | 14 | 993599 | 2.97E-16 | 0.33 | 233 | 3.81E-10 | 15.5 | genome | Wall bleeding front | A | G |
| rs324371668 | 14 | 1102290 | 5.05E-10 | 0.08 | 234 | 4.32E-04 | 9.3 | genome | Tail exudate | A | G |
| rs699809009 | 14 | 2066273 | 5.27E-11 | 0.14 | 234 | 1.35E-04 | 10.3 | genome | Tail tip score | T | C |
| rs333554635 | 14 | 8070158 | 3.25E-18 | 0.05 | 230 | 4.16E-12 | 17.5 | genome | Wall bleeding hind | A | T |
| rs321860975 | 14 | 8630844 | 2.29E-11 | 0.07 | 230 | 9.81E-06 | 10.6 | genome | Wall bleeding hind | G | A |
| rs338522367 | 14 | 26263106 | 5.04E-09 | 0.15 | 233 | 2.16E-03 | 8.3 | chromosome | Wall bleeding front | G | A |
| rs338395204 | 14 | 26593306 | 6.61E-12 | 0.14 | 233 | 4.24E-06 | 11.2 | genome | Wall bleeding front | T | C |
| rs320799300 | 14 | 56443800 | 7.60E-11 | 0.06 | 234 | 3.90E-05 | 10.1 | genome | Tail swelling | A | G |
| rs323488836 | 14 | 75918198 | 2.18E-13 | 0.15 | 234 | 1.40E-07 | 12.7 | genome | Tail swelling | A | G |
| rs319638183 | 14 | 84853550 | 1.49E-21 | 0.14 | 230 | 1.91E-15 | 20.8 | genome | Nose back edema | C | T |
| rs345801073 | 14 | 90016513 | 3.25E-10 | 0.26 | 230 | 1.39E-04 | 9.5 | genome | Nose back edema | A | G |
| rs3470725133 | 14 | 90932616 | 2.91E-10 | 0.10 | 230 | 1.39E-04 | 9.5 | genome | Nose back edema | G | A |
| rs320348093 | 14 | 91588343 | 2.30E-08 | 0.10 | 230 | 7.36E-03 | 7.6 | chromosome | Nose back edema | C | T |
| rs342612561 | 14 | 91808934 | 3.63E-14 | 0.15 | 230 | 2.33E-08 | 13.4 | genome | Wall bleeding hind | C | T |
| rs330551796 | 14 | 100186814 | 3.11E-12 | 0.14 | 234 | 3.99E-06 | 11.5 | genome | Teat necrosis | T | C |
| rs327555764 | 14 | 111411277 | 1.93E-08 | 0.07 | 234 | 5.51E-03 | 7.7 | chromosome | Tail swelling | G | C |
| rs324202033 | 14 | 118250950 | 3.04E-09 | 0.20 | 234 | 7.79E-03 | 8.5 | genome | Tail scabs | G | A |
| rs698043782 | 14 | 126453069 | 5.65E-09 | 0.08 | 234 | 3.62E-03 | 8.2 | chromosome | Ear score | C | T |
| rs324993441 | 14 | 128639642 | 2.68E-08 | 0.23 | 233 | 9.84E-03 | 7.6 | chromosome | Wall bleeding front | A | G |
| rs340142193 | 15 | 10126780 | 2.70E-23 | 0.13 | 234 | 2.31E-17 | 22.6 | genome | Tail swelling | A | G |
| rs345453829 | 15 | 24838653 | 3.05E-09 | 0.12 | 229 | 7.83E-03 | 8.5 | genome | Wall score | A | C |
| rs335716920 | 15 | 27275926 | 2.63E-09 | 0.22 | 234 | 2.25E-03 | 8.6 | genome | Ear score | G | A |
| rs325378176 | 15 | 75578605 | 6.50E-15 | 0.14 | 225 | 8.34E-09 | 14.2 | genome | SINS | G | C |
| rs339270582 | 15 | 76926106 | 1.71E-17 | 0.14 | 230 | 1.47E-11 | 16.8 | genome | Wall bleeding hind | G | A |
| 15_76941405 | 15 | 76941405 | 9.24E-13 | 0.15 | 234 | 2.37E-06 | 12 | genome | Ears shiny skin |  |  |
| rs335520546 | 15 | 76941405 | 2.22E-13 | 0.15 | 234 | 5.71E-07 | 12.7 | genome | Ear score | G | C |
| rs338362730 | 15 | 120350252 | 6.60E-12 | 0.24 | 233 | 1.69E-05 | 11.2 | genome | Heels score | C | T |
| rs344579823 | 15 | 129041019 | 3.14E-09 | 0.31 | 234 | 8.05E-03 | 8.5 | genome | Ears vein congestion | G | A |
| rs346249294 | 16 | 371042 | 4.47E-15 | 0.10 | 234 | 6.34E-09 | 14.4 | genome | Tail exudate | A | G |
| rs319346920 | 16 | 422104 | 2.04E-11 | 0.19 | 234 | 1.45E-05 | 10.7 | genome | Tail exudate | A | G |
| rs331979572 | 16 | 5072741 | 1.50E-12 | 0.18 | 233 | 2.13E-06 | 11.8 | genome | Coronary band inflammation front | A | T |
| rs330625782 | 16 | 18970172 | 1.64E-08 | 0.13 | 229 | 4.66E-03 | 7.8 | chromosome | Wall score | A | G |
| rs318637041 | 16 | 24090428 | 3.30E-10 | 0.06 | 234 | 1.17E-04 | 9.5 | genome | Ears shiny skin | T | A |
| rs331217455 | 16 | 44669358 | 5.42E-12 | 0.16 | 234 | 7.70E-06 | 11.3 | genome | Heels Bleeding hind | A | G |
| rs322693439 | 16 | 47984548 | 6.83E-09 | 0.06 | 230 | 9.70E-03 | 8.2 | chromosome | Nose back edema | G | T |
| rs1108479026 | 16 | 53952975 | 1.34E-10 | 0.16 | 233 | 1.90E-04 | 9.9 | genome | Heels score | G | A |
| rs324596069 | 16 | 62806092 | 1.21E-08 | 0.14 | 234 | 1.72E-02 | 7.9 | chromosome | Ears vein congestion | C | T |
| rs329854729 | 16 | 63636863 | 8.25E-12 | 0.17 | 234 | 5.85E-06 | 11.1 | genome | Ears shiny skin | A | T |
| rs318526142 | 16 | 71895984 | 1.42E-11 | 0.12 | 234 | 1.01E-05 | 10.8 | genome | Tail scabs | A | T |
| rs702105304 | 16 | 73639946 | 9.92E-10 | 0.15 | 234 | 1.41E-03 | 9 | genome | Tail base score | A | G |
| rs333860307 | 16 | 75464401 | 3.35E-11 | 0.09 | 234 | 1.58E-05 | 10.5 | genome | Ears shiny skin | G | T |
| rs337229945 | 17 | 7734948 | 3.60E-09 | 0.25 | 234 | 2.56E-03 | 8.4 | genome | Tail base score | T | G |
| rs329704525 | 17 | 12412530 | 5.04E-12 | 0.06 | 234 | 5.85E-06 | 11.3 | genome | Ears shiny skin | G | T |
| rs3476426989 | 17 | 13518191 | 1.15E-10 | 0.12 | 229 | 4.09E-05 | 9.9 | genome | Wall score | G | A |
| rs321021079 | 17 | 30561262 | 6.75E-15 | 0.06 | 234 | 9.58E-09 | 14.2 | genome | Tail scabs | G | A |
| 17_30666157 | 17 | 30666157 | 1.70E-08 | 0.07 | 233 | 2.41E-02 | 7.8 | chromosome | Wall bleeding front |  |  |
| rs80875010 | 17 | 30666157 | 2.57E-18 | 0.07 | 229 | 3.64E-12 | 17.6 | genome | Wall score | C | T |
| rs329424770 | 17 | 32863890 | 3.81E-09 | 0.11 | 234 | 2.70E-03 | 8.4 | chromosome | Heels Bleeding hind | T | C |
| rs341628611 | 17 | 40157128 | 1.71E-15 | 0.15 | 229 | 1.22E-09 | 14.8 | genome | Wall score | G | A |
| rs342812393 | 17 | 57012344 | 5.68E-10 | 0.09 | 234 | 2.01E-04 | 9.2 | genome | Tail exudate | T | A |
| rs329612020 | 17 | 57447033 | 1.22E-09 | 0.07 | 234 | 8.68E-04 | 8.9 | genome | Tail base redness | C | A |
| rs329323638 | 17 | 57531936 | 7.28E-11 | 0.32 | 229 | 3.44E-05 | 10.1 | genome | Wall score | T | C |
| rs3473148823 | 18 | 1374220 | 4.13E-09 | 0.05 | 234 | 1.47E-03 | 8.4 | chromosome | Tail scabs | A | G |
| rs1110145306 | 18 | 7597561 | 7.58E-09 | 0.46 | 234 | 3.59E-03 | 8.1 | chromosome | Tail base score | T | C |
| rs710514292 | 18 | 8384320 | 3.10E-09 | 0.47 | 234 | 1.47E-03 | 8.5 | genome | Tail base redness | C | T |
| rs706720575 | 18 | 10834858 | 4.47E-08 | 0.17 | 234 | 1.27E-02 | 7.3 | chromosome | Tail base redness | G | C |
| rs340268930 | 18 | 23346365 | 6.51E-12 | 0.06 | 234 | 9.23E-06 | 11.2 | genome | Tail tip score | C | T |
| rs324500936 | 18 | 30764926 | 9.13E-13 | 0.05 | 234 | 1.30E-06 | 12 | genome | Tail base redness | G | A |
| rs345137043 | 18 | 42907967 | 5.65E-12 | 0.21 | 229 | 8.02E-06 | 11.2 | genome | Coronary band score | G | A |
| rs327672558 | 18 | 42921245 | 1.49E-10 | 0.20 | 230 | 2.11E-04 | 9.8 | genome | Coronary band inflammation hind | G | A |
| rs320301831 | 18 | 43223181 | 4.06E-10 | 0.08 | 234 | 1.92E-04 | 9.4 | genome | Tail exudate | C | T |
| rs1110489293 | 18 | 46555922 | 1.67E-08 | 0.07 | 234 | 5.93E-03 | 7.8 | chromosome | Tail base redness | A | G |
| rs324264677 | 18 | 47288714 | 1.47E-10 | 0.05 | 234 | 2.09E-04 | 9.8 | genome | Tail base no bristles | T | C |
| rs3470367968 | 18 | 48117118 | 1.06E-09 | 0.16 | 234 | 5.03E-04 | 9 | genome | Tail scabs | A | C |
| X_121002068 | X | 122362632 | 1.20E-13 | 0.11 | 236 | 4.39E-09 | 12.9 | genome | Tail bleedingn | A | G |
| X_32069776 | X | 32069776 | 1.77E-10 | 0.13 | 236 | 6.80E-05 | 9.8 | genome | Tail base swelling | G | A |

Light grey: SNPS with a distance below 1 Million bp, associated with a similar phenotype. Dark grey; SNPs with a distance below 1 Million bp associated with different phenotypes. Slightly differences in grey were used to differentiate between two neighbouring positions.

**Supplemental Table 3**  Phenotypes associated with chromosomal regions (<1 Mio bp) and additional phenotypes

|  |  | Phenotypes | Additional phenotypes |  |  |
| --- | --- | --- | --- | --- | --- |
| SSC-Locus | Position | GWAS | ANOVA | R^2^ | -logP |
| 1,1 | 14157691 | Tail swelling | Tail no bristles | 15.5 | 8.5 |
| 1,2 | 31190769 | Tail swelling | Tail base no bristles | 25.0 | 14.6 |
|  |  |  | Tail base swelling | 23.3 | 13.4 |
|  |  |  | Tail no bristles | 19.6 | 11.0 |
|  |  |  | SINS | 17.6 | 9.4 |
| 1,3 | 35812331 | Tail tip score | Tail no bristles | 23.6 | 13.6 |
|  |  |  | Tail tip score | 19.1 | 10.7 |
| 1,4 | 56360947 | Ears vein congestion | Tail base no bristles | 25.7 | 14.9 |
|  |  |  | Tail base score | 21.3 | 12.1 |
|  |  |  | Tail base swelling | 21.3 | 12.1 |
| 1,5 | 66905225 | Wall bleeding hind | Ear score | 20.1 | 11.4 |
|  |  |  | Ears shiny skin | 17.1 | 9.5 |
|  |  |  | SINS | 17.4 | 9.3 |
|  |  |  | Wall bleeding hind | 18.0 | 9.8 |
|  |  |  | Wall score | 17.2 | 9.3 |
| 1,6 | 212887602 | Tail swelling | Tail base swelling | 25.0 | 14.6 |
|  |  | SINS | Ear score | 20.9 | 11.8 |
|  |  |  | Ears shiny skin | 18.1 | 10.1 |
|  |  |  | SINS | 17.3 | 9.2 |
|  |  |  | Teats vein congestion | 15.5 | 8.5 |
| 1,7 | 243296440 | Ear score | Ear score | 14.7 | 8.1 |
| 1,8 | 253523847 | Teat necrosis | Ear score | 19.9 | 11.2 |
|  |  |  | Ears no bristles | 16.0 | 8.8 |
|  |  |  | Ears shiny skin | 19.4 | 10.9 |
| 1,9 | 271201482 | Tail base score | Ear score | 16.4 | 9.0 |
| 2,1 | 8270586 | Ears vein congestion | Ears vein congestion | 16.2 | 8.9 |
| 2,2 | 15794455 | Wall score | Wall score | 16.4 | 8.9 |
| 2,3 | 39324482 | Wall bleeding hind | Wall bleeding hind | 26.3 | 15.1 |
|  |  |  | Wall bleeding front | 22.7 | 13.0 |
|  |  | Wall score | Wall score | 27.2 | 15.7 |
|  | 39322886 | Wall score | Wall bleeding hind | 21.3 | 11.9 |
| 2,4 | 104384195 | Ears no bristles | Ears no bristles | 15.0 | 8.2 |
| 2,5 | 140645128 | Wall bleeding hind | Ears no bristles | 18.4 | 10.3 |
|  |  |  | Ear score | 17.6 | 9.8 |
| 3,1 | 22988098 | Wall bleeding front | Wall bleeding front | 15.1 | 8.2 |
|  |  | Wall score |  |  |  |
| 3,2 | 124855843 | Ears vein congestion | Tail base no bristles | 26.1 | 15.3 |
|  |  |  | Tail base score | 21.0 | 11.9 |
|  |  |  | Tail base swelling | 22.5 | 12.9 |
| 4,1 | 106109743 | Nose back edema | SINS | 17.3 | 9.2 |
| 5,1 | 68033728 | Heels score | SINS | 20.5 | 11.1 |
| 5,2 | 83317374 | Heels score | Tail base no bristles | 25.1 | 14.5 |
| 5,3 | 85068157 | Face score | SINS | 18.1 | 9.7 |
| 6,1 | 10345325 | Wall bleeding hind | SINS | 16.1 | 8.5 |
| 6,2 | 18668130 | Face score | Tail base swelling | 21.5 | 12.2 |
|  |  |  | SINS | 18.5 | 9.9 |
| 7,1 | 21333865 | Tail tip cabs | Tail tip scabs | 15.8 | 8.7 |
| 7,2 | 26025361 | Tail swelling | SINS | 16.4 | 8.6 |
|  |  |  | Tail base no bristles | 16.9 | 9.3 |
|  |  |  | Tail base swelling | 15.5 | 8.5 |
|  |  |  | Tail no bristles | 21.0 | 11.8 |
| 7,3 | 49086851 | Face score | Ears no bristles | 17.7 | 9.8 |
|  |  |  | Ear score | 16.2 | 9.0 |
| 8,1 | 1208125 | Wall bleeding hind | Wall score | 20.2 | 11.2 |
|  |  |  | Wall bleeding hind | 19.6 | 10.8 |
| 8,2 | 136683047 | Nose back edema | Nose back edema | 23.8 | 13.5 |
| 8,3 | 138016195 | Nose back edema | Nose back edema | 24.8 | 14.2 |
| 9,1 | 5511145 | Wall bleeding hind | Ear score | 14.9 | 8.2 |
| 9,2 | 90241577 | Wall bleeding front | Ear score | 23.7 | 13.7 |
|  |  |  | Wall bleeding hind | 20.2 | 11.2 |
|  |  |  | Wall score | 20.3 | 11.2 |
|  |  |  | Ears shiny skin | 19.5 | 11.0 |
|  |  |  | Ears no bristles | 17.0 | 9.4 |
|  |  |  | SINS | 16.5 | 8.8 |
|  |  |  | Teat vein congestion | 15.7 | 8.7 |
| 10,1 | 3028232 | Tail tip score | SINS | 16.8 | 9.0 |
|  |  |  | Tail tip score | 21.8 | 12.5 |
| 10,2 | 49609959 | Wall bleeding hind | Ear score | 25.9 | 15.1 |
|  |  |  | Tail base no bristles | 25.0 | 14.6 |
|  |  |  | Ears no bristles | 21.9 | 12.5 |
|  |  |  | Tail base swelling | 21.5 | 12.3 |
|  |  |  | Ears shiny skin | 19.9 | 11.2 |
|  |  |  | Tail base score | 19.7 | 11.1 |
| 11,1 | 7470748 | Tail tip score | Tail tip scabs | 22.3 | 12.7 |
| 11,2 | 16097396 | Ears shiny skin | Ears shiny skin | 16.0 | 8.8 |
| 11,3 | 21365824 | Coronary bands front | Ear score | 15.3 | 8.4 |
|  |  |  | Ears shiny skin | 14.9 | 8.2 |
| 11,4 | 21453639 | Coronary bands score | SINS | 15.8 | 8.3 |
| 11,5 | 70939109 | Wall bleeding hind | Ear score | 22.2 | 12.7 |
|  |  |  | Ears no bristles | 17.6 | 9.8 |
|  |  |  | Ears shiny skin | 18.8 | 10.5 |
| 11,6 | 71239679 | Ears shiny skin | Ear score | 21.1 | 11.9 |
|  |  |  | Ears shiny skin | 17.8 | 9.8 |
|  |  |  | Wall bleeding hind | 17.8 | 9.7 |
|  |  |  | Wall score | 19.1 | 9.2 |
|  |  |  | SINS | 16.3 | 8.6 |
|  |  |  | Ears no bristles | 15.5 | 8.5 |
| 12,1 | 14407795 | Wall bleeding hind | Ear score | 18.7 | 10.5 |
|  |  |  | Ears no bristles | 15.9 | 8.7 |
|  |  |  | Ears shiny skin | 16.1 | 8.9 |
|  |  |  | Teats vein congestion | 15.7 | 8.6 |
| 12,2 | 14498120 | Wall bleeding hind | Ear score | 18.9 | 10.6 |
|  |  |  | Ears no bristles | 15.6 | 8.6 |
|  |  |  | Ears shiny skin | 17.7 | 9.9 |
|  |  |  | Teats vein congestion | 15.1 | 8.3 |
| 12,3 | 15058247 | Coronary bands front | Tail base no bristles | 17.0 | 9.4 |
| 12,4 | 34198370 | Tail tip cabs | Tail tip scabs | 18.2 | 10.1 |
| 12,5 | 44738423 | Heels bleeding hind | Heels bleeding hind | 17.4 | 9.7 |
|  |  |  | SINS | 19.7 | 10.7 |
| 12,6 | 50607044 | Ear score | Ear score | 17.4 | 9.7 |
|  |  |  | Ears shiny skin | 15.4 | 8.4 |
| 13,1 | 9300436 | Wall bleeding hind | Wall bleeding front | 18.9 | 10.6 |
|  |  |  | Wall bleeding hind | 20.6 | 11.5 |
|  |  |  | Wall score | 24.3 | 13.8 |
| 13,2 | 13362832 | Teat necrosis | SINS | 19.7 | 10.6 |
|  |  |  | Ears no bristles | 18.2 | 10.1 |
|  |  |  | Ear score | 17.7 | 9.8 |
|  |  |  | Teats vein congestion | 15.6 | 8.6 |
|  |  |  | Ears shiny skin | 15.6 | 8.6 |
| 13,3 | 24140025 | Ears no bristles | Ear score | 22.4 | 12.8 |
|  |  |  | Ears no bristles | 24.2 | 14.0 |
|  |  |  | Ears shiny skin | 24.9 | 14.5 |
| 13,4 | 34980722 | Wall bleeding hind | Wall bleeding hind | 19.6 | 10.8 |
| 13,5 | 53856072 | Tail base score | Ears no bristles | 18.8 | 10.5 |
|  |  |  | Ears shiny skin | 18.1 | 10.1 |
|  |  |  | Ear score | 17.7 | 9.8 |
| 13,6 | 56534476 | Wall bleeding hind | Wall bleeding hind | 18.9 | 10.4 |
|  |  |  | Wall score | 18.7 | 10.2 |
| 13,7 | 148170559 | Tail base redness | Ear score | 16.3 | 9.0 |
|  |  |  | Ears no bristles | 14.8 | 8.1 |
|  |  |  | Ears shiny skin | 14.7 | 8.1 |
|  |  |  | Tail base score | 17.2 | 9.5 |
| 13,8 | 208309290 | Wall bleeding front | Wall bleeding front | 25.0 | 14.5 |
|  |  |  | Wall bleeding hind | 17.4 | 9.5 |
|  |  |  | Wall score | 24.1 | 13.6 |
| 14,1 | 75918198 | Tail swelling | Ear score | 22.0 | 12.5 |
|  |  |  | Ears shiny skin | 17.4 | 9.7 |
|  |  |  | Wall bleeding hind | 17.7 | 9.6 |
|  |  |  | Wall score | 19.2 | 9.3 |
|  |  |  | Ears no bristles | 15.5 | 8.5 |
|  |  |  | SINS | 15.6 | 8.2 |
| 14,2 | 91808934 | Wall bleeding hind | Ear score | 23.4 | 13.5 |
|  |  |  | Ears no bristles | 16.6 | 9.2 |
|  |  |  | Ears shiny skin | 19.8 | 11.2 |
|  |  |  | SINS | 18.0 | 9.7 |
|  |  |  | Wall bleeding hind | 17.3 | 9.4 |
|  |  |  | Wall score | 16.7 | 9.1 |
| 14,3 | 100186814 | Teat necrosis | Ear score | 17.7 | 9.8 |
|  |  |  | Ears no bristles | 15.3 | 8.4 |
|  |  |  | Ears shiny skin | 15.6 | 8.6 |
| 15,1 | 27275926 | Ear score | Ear score | 19.5 | 10.9 |
|  |  |  | Ears vein congestion | 17.0 | 9.3 |
|  |  |  | SINS | 20.3 | 10.9 |
|  |  |  | Tail base swelling | 18.6 | 10.3 |
| 15,2 | 75578605 | SINS | Ear score | 22.1 | 12.7 |
|  |  |  | Ears no bristles | 16.9 | 9.4 |
|  |  |  | Ears shiny skin | 19.0 | 10.7 |
|  |  |  | SINS | 17.8 | 9.6 |
|  |  |  | Wall bleeding hind | 17.2 | 9.4 |
|  |  |  | Wall score | 16.7 | 9.0 |
| 15,3 | 76926106 | Wall bleeding hind | Ear score | 30.7 | 18.6 |
|  |  |  | Ears no bristles | 20.9 | 11.9 |
|  |  |  | Ears shiny skin | 26.8 | 15.8 |
|  |  |  | Teat vein congestion | 16.7 | 9.3 |
|  |  |  | SINS | 15.3 | 8.0 |
| 15,4 | 76941405 | Ear score | Ear score | 30.7 | 18.6 |
|  |  |  | Teat vein congestion | 16.7 | 9.3 |
|  | 76941405 | Ears shiny skin | Ears no bristles | 20.9 | 11.9 |
|  |  |  | Ears shiny skin | 26.8 | 15.8 |
|  |  |  | SINS | 18.6 | 10.0 |
|  |  |  | Wall bleeding hind | 17.9 | 9.7 |
|  |  |  | Wall score | 17.4 | 9.4 |
| 16,1 | 18970172 | Wall score | Wall bleeding front | 18.2 | 10.0 |
|  |  |  | Wall bleeding hind | 15.5 | 8.3 |
|  |  |  | Wall score | 21.0 | 11.5 |
| 16,2 | 44669358 | Heels bleeding hind | Ear score | 23.9 | 13.8 |
|  |  | Nose back edema | Ears no bristles | 16.4 | 9.0 |
|  |  |  | Ears shiny skin | 19.0 | 10.6 |
|  |  |  | SINS | 19.5 | 10.5 |
|  |  |  | Teats vein congestion | 16.0 | 8.8 |
|  | 47984548 |  | Nose back edema | 19.0 | 10.5 |
| 16,3 | 53952975 | Heels score | SINS | 21.4 | 11.7 |
| 16,4 | 62806092 | Ears vein congestion | Ears vein congestion | 17.0 | 9.5 |
| 16,5 | 63636863 | Ears shiny skin | Ear score | 16.8 | 9.3 |
|  |  |  | Ears no bristles | 16.9 | 9.4 |
|  |  |  | Ears shiny skin | 25.6 | 14.9 |
| 17,1 | 7734948 | Tail base score | Tail base score | 15.1 | 8.3 |
| 17,2 | 30666157 | Wall bleeding front | Tail tip scabs | 16.4 | 9.0 |
|  |  | Wall score | Wall bleeding front | 16.6 | 9.1 |
|  |  |  | Wall score | 17.3 | 9.4 |
| 17,3 | 40157128 | Wall score | Ear score | 20.3 | 11.4 |
|  |  |  | Ears shiny skin | 17.9 | 9.9 |
|  |  |  | SINS | 15.7 | 8.3 |
|  |  |  | Wall bleeding hind | 20.9 | 11.6 |
|  |  |  | Wall score | 21.2 | 11.7 |
| 17,4 | 57531936 | Wall score | Wall bleeding hind | 18.1 | 9.9 |
|  |  |  | Wall score | 19.4 | 10.7 |
| 18,1 | 1374220 | Tail tip cabs | Tail tip scabs | 16.7 | 9.3 |
| 18,2 | 7597561 | Tail base score | Tail base no bristles | 20.8 | 11.8 |
|  |  |  | Tail base score | 23.5 | 13.5 |
|  |  |  | Tail base swelling | 14.9 | 8.2 |
| 18,3 | 8384320 | Tail base redness | Tail base redness | 22.2 | 12.7 |
|  |  |  | Tail base score | 16.4 | 9.1 |
| X,1 | 32069776 | Tail base swelling | Tail base score | 16.2 | 8.9 |
| X,2 | 122362632 | Tail bleeding | SINS | 17.2 | 9.1 |

**Supplemental Table 4**  SNPs associated with the 25 significant phenotypic SINS signs after GWAS

| Phenotypic SINS sign | Associated SNPs |
| --- | --- |
| Coronary band inflammation front | 9_5432233 6_43583063 16_5072741 13_42212034 12_15058247 11_21365824 1_273401709 |
| Coronary band inflammation hind | 18_42921245 |
| Coronary band score | 8_131758708 6_45590628 4_11059334 3_60845061 18_42907967 11_21453639 10_20062150 |
| Ear score | 8_127385592 7_9776278 5_86636102 4_15775626 2_86288691 2_13484586 15_76941405 15_27275926 14_126453069 13_34502858 12_54902440 12_50607044 12_16344077 11_18448156 1_271968856 1_243296440 |
| Ears no bristles | 5_66261808 2_104384195 13_24140025 1_47534745 |
| Ears shiny skin | 9_20779267 7_7831022 6_126898383 4_89152946 17_12412530 16_75464401 16_63636863 16_24090428 15_76941405 13_193355481 12_17689507 11_71239679 11_16097396 |
| Ears vein congestion | 5_46569482 3_75265156 3_124855843 2_8270586 2_118205756 16_62806092 15_129041019 1_56360947 |
| Eye edema | 3_64131673 |
| Face score | 7_49086851 6_18668130 5_85068157 |
| Heels Bleeding hind | 6_152291242 5_80628823 5_100172331 17_32863890 16_44669358 12_44738423 11_72467167 |
| Heels score | 5_83317374 5_68033728 16_53952975 15_120350252 13_147948513 |
| Nose back edema | 9_70120548 9_17634045 8_72440718 8_138016195 8_137928158 8_136683047 6_127841510 4_106109743 3_30546515 16_47984548 14_91588343 14_90932616 14_90016513 14_84853550 13_6150834 13_20840635 13_205064145 13_204539983 12_40475317 12_15265438 10_35048601 10_21027752 |
| SINS | 4_91282935 4_4813837 3_30052974 15_75578605 13_4986899 1_4508329 1_212887602 |
| Tail base no bristles | 7_6378335 7_43205907 3_55171658 18_47288714 |
| Tail base redness | 4_72908203 18_8384320 18_46555922 18_30764926 18_10834858 17_57447033 13_148170559 12_10426293 |
| Tail base score | 3_55155672 3_1988881 18_7597561 17_7734948 16_73639946 13_53856072 11_24197536 1_271201482 |
| Tail exudate | 18_43223181 17_57012344 16_422104 16_371042 14_1102290 13_192132372 13_125703968 10_8414213 1_16224794 |
| Tail base swelling | X_32069776 |
| Tail scabs | 7_37255799 7_21333865 3_53445482 3_16013886 18_48117118 18_1374220 17_30561262 16_71895984 14_118250950 12_34198370 |
| Tail swelling | 9_137464138 7_97946588 7_26025361 7_22949346 6_87438411 6_153984413 3_101050773 2_23751648 15_10126780 14_75918198 14_56443800 14_111411277 13_31441960 13_25884213 13_144290398 13_131787576 13_130690390 1_75609421 1_31190769 1_14157691 |
| Tail tip score | 18_23346365 14_2066273 11_74707488 10_38611132 10_36728786 10_30282321 1_35812331 |
| Teat necrosis | 14_100186814 13_50633738 13_48553790 13_13362832 1_253523847 1_224713985 1_19763293 |
| Teat swelling | 5_74130702 |
| Wall bleeding front | 9_90241577 9_40724411 9_13753130 7_96238503 3_22988098 17_30666157 14_993599 14_556732 14_26593306 14_26263106 14_128639642 13_43345670 13_208309290 12_39668035 1_274101520 |
| Wall bleeding hind | 9_5511145 9_2974915 8_1208125 6_10345325 5_81674515 4_2511700 2_39322886 2_140645128 15_76926106 14_91808934 14_8630844 14_8070158 13_9300436 13_56534476 13_34980722 12_14498120 12_14407795 11_70939109 10_49785901 10_49609959 1_66905225 |
| Wall score | 3_22988098 2_39324482 2_15794455 17_57531936 17_40157128 17_30666157 17_13518191 16_18970172 15_24838653 10_4367731 |

**Supplemental Table 5**  Effects of GWAS Genotypes on SINS-phenotypes

| SSC | Position | SSC-region | Phenotype GWAS | Associated phenotype Anova | GT1 | GT2 | GT3 | N1 | N2 | N3 | Mean1 | Mean2 | Mean3 | SE1 | SE2 | SE3 | Unfavorable allele | Favourable allele | R2 | -logP |
| --- | --- | --- | --- | --- | --- | --- | --- | --- | --- | --- | --- | --- | --- | --- | --- | --- | --- | --- | --- | --- |
| 15 | 76926106 | 15, 3 | Wall bleeding hind | Ear score | GG | GC | CC | 7 | 58 | 171 | 0.29 | 1.59 | 2.73 | 0.18 | 1.29 | 0.07 | C | G | 30.7 | 18.6 |
| 15 | 76941405 | 15, 4 | Ear score | Ear score | GG | GC | CC | 7 | 58 | 171 | 0.29 | 1.59 | 2.73 | 0.18 | 1.29 | 0.07 | C | G | 30.7 | 18.6 |
| 2 | 39324482 | 2, 3 | Wall score | Wall score | TT | TC | CC | 201 | 29 | 1 | 1.85 | 0.93 | 0.00 | 0.03 | 0.59 | 0.00 | T | C | 27.2 | 15.7 |
| 15 | 76926106 | 15, 3 | Wall bleeding hind | Ears shiny skin | GG | GC | CC | 7 | 58 | 171 | 0.00 | 0.45 | 0.88 | 0.00 | 0.32 | 0.03 | C | G | 26.8 | 15.8 |
| 15 | 76941405 | 15, 4 | Ear score | Ears shiny skin | GG | GC | CC | 7 | 58 | 171 | 0.00 | 0.45 | 0.88 | 0.00 | 0.32 | 0.03 | C | G | 26.8 | 15.8 |
| 2 | 39322886 | 2, 3 | Wall bleeding hind | Wall score | TT | CT | CC | 1 | 29 | 201 | 1.00 | 0.90 | 1.85 | 0.00 | 0.55 | 0.03 | C | T | 26.3 | 15.1 |
| 3 | 124855843 | 3, 2 | Ears vein congestion | Tail base no bristles | GG | GA | AA | 167 | 68 | 1 | 0.19 | 0.72 | 1.00 | 0.03 | 0.61 | 0.00 | A | G | 26.1 | 15.3 |
| 10 | 49609959 | 10, 2 | Wall bleeding hind | Ear score | GG | GA | AA | 4 | 58 | 174 | 0.00 | 1.60 | 2.69 | 0.00 | 1.30 | 0.07 | A | G | 25.9 | 15.1 |
| 1 | 56360947 | 1, 4 | Ears vein congestion | Tail base no bristles | TT | CT | CC | 1 | 64 | 170 | 1.00 | 0.73 | 0.20 | 0.00 | 0.62 | 0.03 | T | C | 25.7 | 14.9 |
| 16 | 63636863 | 16, 5 | Ears shiny skin | Ears shiny skin | AA | AT | TT | 164 | 63 | 9 | 0.88 | 0.51 | 0.00 | 0.03 | 0.38 | 0.00 | A | T | 25.6 | 14.9 |
| 5 | 83317374 | 5, 2 | Heels score | Tail base no bristles | GG | TG | TT | 3 | 59 | 173 | 0.67 | 0.75 | 0.20 | 0.33 | 0.63 | 0.03 | G | T | 25.1 | 14.5 |
| 10 | 3028232 | 10, 1 | Tail tip score | Tail base no bristles | TT | CT | CC | 3 | 67 | 166 | 0.67 | 0.72 | 0.19 | 0.33 | 0.61 | 0.03 | T | C | 25.0 | 14.6 |
| 1 | 31190769 | 1, 2 | Tail swelling | Tail base no bristles | AA | CA | CC | 3 | 67 | 166 | 0.67 | 0.72 | 0.19 | 0.33 | 0.61 | 0.03 | A | C | 25.0 | 14.6 |
| 13 | 208309290 | 13, 8 | Wall bleeding front | Wall bleeding front | GG | GA | AA | 204 | 30 | 1 | 0.93 | 0.43 | 0.00 | 0.02 | 0.25 | 0.00 | G | A | 25.0 | 14.5 |
| 13 | 24140025 | 13, 3 | Ears no bristles | Ears shiny skin | GG | GA | AA | 179 | 54 | 3 | 0.87 | 0.39 | 0.00 | 0.03 | 0.25 | 0.00 | G | A | 24.9 | 14.5 |
| 8 | 138016195 | 8, 3 | Nose back edema | Nose back edema | GG | CG | CC | 4 | 39 | 189 | 1.00 | 0.18 | 0.04 | 0.00 | 0.05 | 0.01 | G | C | 24.8 | 14.2 |
| 13 | 9300436 | 13, 1 | Wall bleeding hind | Wall score | GG | GC | CC | 210 | 17 | 4 | 1.82 | 0.82 | 0.50 | 0.03 | 0.33 | 0.50 | G | C | 24.3 | 13.8 |
| 13 | 24140025 | 13, 3 | Ears no bristles | Ears no bristles | GG | GA | AA | 179 | 54 | 3 | 0.79 | 0.26 | 0.00 | 0.03 | 0.14 | 0.00 | G | A | 24.2 | 14.0 |
| 13 | 208309290 | 13, 8 | Wall bleeding front | Wall score | GG | GA | AA | 200 | 30 | 1 | 1.85 | 1.00 | 0.00 | 0.03 | 0.66 | 0.00 | G | A | 24.1 | 13.6 |
| 16 | 44669358 | 16, 2 | Heels bleeding hind | Ear score | GG | AG | AA | 11 | 52 | 172 | 0.45 | 1.87 | 2.66 | 0.28 | 1.58 | 0.07 | A | G | 23.9 | 13.8 |
| 8 | 136683047 | 8, 2 | Nose back edema | Nose back edema | GG | GA | AA | 171 | 59 | 2 | 0.01 | 0.24 | 1.00 | 0.01 | 0.13 | 0.00 | A | G | 23.8 | 13.5 |
| 9 | 90241577 | 9, 2 | Wall bleeding front | Ear score | TT | CT | CC | 170 | 59 | 7 | 2.68 | 1.76 | 0.29 | 0.07 | 1.47 | 0.18 | T | C | 23.7 | 13.7 |
| 1 | 35812331 | 1, 3 | Tail tip score | Tail no bristles | AA | AC | CC | 212 | 23 | 1 | 0.04 | 0.43 | 1.00 | 0.01 | 0.22 | 0.00 | C | A | 23.6 | 13.6 |
| 18 | 7597561 | 18, 2 | Tail base score | Tail base score | TT | TC | CC | 53 | 113 | 70 | 2.02 | 1.13 | 0.47 | 0.15 | 0.94 | 0.10 | T | C | 23.5 | 13.5 |
| 14 | 91808934 | 14, 2 | Wall bleeding hind | Ear score | TT | CT | CC | 172 | 58 | 6 | 2.67 | 1.74 | 0.17 | 0.07 | 1.45 | 0.17 | T | C | 23.4 | 13.5 |
| 1 | 31190769 | 1, 2 | Tail swelling | Tail base swelling | AA | CA | CC | 3 | 67 | 166 | 0.67 | 0.79 | 0.27 | 0.33 | 0.69 | 0.03 | A | C | 23.3 | 13.4 |
| 2 | 39324482 | 2, 3 | Wall score | Wall bleeding front | TT | TC | CC | 205 | 29 | 1 | 0.92 | 0.45 | 0.00 | 0.02 | 0.26 | 0.00 | T | C | 22.7 | 13.0 |
| 3 | 124855843 | 3, 2 | Ears vein congestion | Tail base swelling | GG | GA | AA | 167 | 68 | 1 | 0.27 | 0.78 | 1.00 | 0.03 | 0.68 | 0.00 | A | G | 22.5 | 12.9 |
| 13 | 24140025 | 13, 3 | Ears no bristles | Ear score | GG | GA | AA | 179 | 54 | 3 | 2.67 | 1.48 | 1.00 | 0.07 | 1.17 | 0.00 | G | A | 22.4 | 12.8 |
| 11 | 7470748 | 11, 1 | Tail tip score | Tail tip scabs | GG | AG | AA | 2 | 97 | 136 | 1.00 | 0.53 | 0.11 | 0.00 | 0.42 | 0.03 | G | A | 22.3 | 12.7 |
| 18 | 8384320 | 18, 3 | Tail base redness | Tail base redness | TT | CT | CC | 72 | 104 | 60 | 0.19 | 0.26 | 0.75 | 0.05 | 0.17 | 0.06 | C | T | 22.2 | 12.7 |
| 11 | 70939109 | 11, 5 | Wall bleeding hind | Ear score | TT | CT | CC | 4 | 57 | 175 | 0.00 | 1.68 | 2.66 | 0.00 | 1.39 | 0.07 | C | T | 22.2 | 12.7 |
| 15 | 75578605 | 15, 2 | SINS | Ear score | GG | GC | CC | 177 | 56 | 3 | 2.64 | 1.66 | 0.00 | 0.07 | 1.36 | 0.00 | G | C | 22.1 | 12.7 |
| 14 | 75918198 | 14, 1 | Tail swelling | Ear score | GG | AG | AA | 171 | 58 | 6 | 2.66 | 1.79 | 0.17 | 0.07 | 1.51 | 0.17 | G | A | 22.0 | 12.5 |
| 10 | 49609959 | 10, 2 | Wall bleeding hind | Ears no bristles | GG | GA | AA | 4 | 58 | 174 | 0.00 | 0.31 | 0.79 | 0.00 | 0.19 | 0.03 | A | G | 21.9 | 12.5 |
| 10 | 3028232 | 10, 1 | Tail tip score | Tail tip score | TT | CT | CC | 3 | 67 | 166 | 4.67 | 0.96 | 0.52 | 0.33 | 0.66 | 0.06 | T | C | 21.8 | 12.5 |
| 10 | 3028232 | 10, 1 | Tail tip score | Tail base swelling | TT | CT | CC | 3 | 67 | 166 | 0.67 | 0.78 | 0.27 | 0.33 | 0.67 | 0.03 | T | C | 21.5 | 12.3 |
| 6 | 18668130 | 6, 1 | Face score | Tail base swelling | GG | AG | AA | 6 | 64 | 165 | 0.67 | 0.78 | 0.27 | 0.21 | 0.68 | 0.03 | G | A | 21.5 | 12.2 |
| 16 | 53952975 | 16, 3 | Heels score | SINS | GG | GA | AA | 162 | 56 | 9 | 16.89 | 14.80 | 5.44 | 4.77 | 3.53 | 3.43 | G | A | 21.4 | 11.7 |
| 2 | 39322886 | 2, 3 | Wall bleeding hind | Wall bleeding hind | TT | CT | CC | 1 | 29 | 202 | 0.00 | 0.48 | 0.93 | 0.00 | 0.29 | 0.02 | C | T | 21.3 | 11.9 |
| 2 | 39324482 | 2, 3 | Wall score | Wall bleeding hind | TT | TC | CC | 202 | 29 | 1 | 0.93 | 0.48 | 0.00 | 0.02 | 0.29 | 0.00 | T | C | 21.3 | 11.9 |
| 1 | 56360947 | 1, 4 | Ears vein congestion | Tail base swelling | TT | CT | CC | 1 | 64 | 170 | 1.00 | 0.78 | 0.28 | 0.00 | 0.68 | 0.03 | T | C | 21.3 | 12.1 |
| 1 | 56360947 | 1, 4 | Ears vein congestion | Tail base score | TT | CT | CC | 1 | 64 | 170 | 2.00 | 1.98 | 0.81 | 0.00 | 1.70 | 0.08 | T | C | 21.3 | 12.1 |
| 17 | 40157128 | 17, 3 | Wall score | Wall score | GG | GA | AA | 168 | 55 | 7 | 1.80 | 1.75 | 0.14 | 0.04 | 1.58 | 0.14 | G | A | 21.2 | 11.7 |
| 11 | 71239679 | 11, 6 | Ears shiny skin | Ear score | TT | CT | CC | 6 | 51 | 178 | 0.17 | 1.76 | 2.63 | 0.17 | 1.46 | 0.07 | C | T | 21.1 | 11.9 |
| 7 | 26025361 | 7, 2 | Tail swelling | Tail no bristles | GG | CG | CC | 2 | 40 | 192 | 1.00 | 0.28 | 0.03 | 0.00 | 0.13 | 0.01 | G | C | 21.0 | 11.8 |
| 3 | 124855843 | 3, 2 | Ears vein congestion | Tail base score | GG | GA | AA | 167 | 68 | 1 | 0.80 | 1.93 | 3.00 | 0.08 | 1.66 | 0.00 | A | G | 21.0 | 11.9 |
| 16 | 18970172 | 16, 1 | Wall score | Wall score | GG | AG | AA | 10 | 41 | 178 | 1.00 | 1.27 | 1.88 | 0.33 | 0.99 | 0.03 | A | G | 21.0 | 11.5 |
| 15 | 76926106 | 15, 3 | Wall bleeding hind | Ears no bristles | GG | GC | CC | 7 | 58 | 171 | 0.14 | 0.33 | 0.79 | 0.14 | 0.20 | 0.03 | C | G | 20.9 | 11.9 |
| 15 | 76941405 | 15, 4 | Ear score | Ears no bristles | GG | GC | CC | 7 | 58 | 171 | 0.14 | 0.33 | 0.79 | 0.14 | 0.20 | 0.03 | C | G | 20.9 | 11.9 |
| 17 | 40157128 | 17, 3 | Wall score | Wall bleeding hind | GG | GA | AA | 169 | 55 | 7 | 0.90 | 0.89 | 0.00 | 0.02 | 0.81 | 0.00 | G | A | 20.9 | 11.6 |
| 15 | 76941405 | 15, 4 | Ears shiny skin | Ear score | GG | GA | AA | 176 | 52 | 6 | 2.64 | 1.81 | 0.17 | 0.07 | 1.51 | 0.17 | G | A | 20.9 | 11.8 |
| 1 | 212887602 | 1, 6 | SINS | Ear score | AA | AC | CC | 8 | 55 | 172 | 0.88 | 1.67 | 2.67 | 0.48 | 1.37 | 0.07 | C | A | 20.9 | 11.8 |
| 18 | 7597561 | 18, 2 | Tail base score | Tail base no bristles | TT | TC | CC | 53 | 113 | 70 | 0.72 | 0.32 | 0.11 | 0.06 | 0.23 | 0.04 | T | C | 20.8 | 11.8 |
| 13 | 9300436 | 13, 1 | Wall bleeding hind | Wall bleeding hind | GG | GC | CC | 211 | 17 | 4 | 0.91 | 0.41 | 0.25 | 0.02 | 0.15 | 0.25 | G | C | 20.6 | 11.5 |
| 5 | 68033728 | 5, 1 | Heels score | SINS | GG | AG | AA | 26 | 58 | 142 | 20.00 | 18.28 | 14.23 | 5.08 | 4.24 | 4.47 | G | A | 20.5 | 11.1 |
| 9 | 90241577 | 9, 2 | Wall bleeding front | Wall score | TT | CT | CC | 166 | 58 | 7 | 1.79 | 1.74 | 0.14 | 0.04 | 1.57 | 0.14 | T | C | 20.3 | 11.2 |
| 15 | 27275926 | 15, 1 | Ear score | SINS | GG | GA | AA | 143 | 71 | 11 | 14.24 | 18.46 | 21.00 | 4.22 | 4.85 | 5.71 | A | G | 20.3 | 10.9 |
| 17 | 40157128 | 17, 3 | Wall score | Ear score | GG | GA | AA | 172 | 56 | 7 | 2.63 | 1.84 | 0.29 | 0.07 | 1.53 | 0.18 | G | A | 20.3 | 11.4 |
| 9 | 90241577 | 9, 2 | Wall bleeding front | Wall bleeding hind | TT | CT | CC | 167 | 58 | 7 | 0.90 | 0.88 | 0.00 | 0.02 | 0.79 | 0.00 | T | C | 20.2 | 11.2 |
| 8 | 1208125 | 8, 1 | Wall bleeding hind | Wall score | GG | GA | AA | 209 | 21 | 1 | 1.82 | 0.86 | 1.00 | 0.04 | 0.44 | 0.00 | G | A | 20.2 | 11.2 |
| 1 | 66905225 | 1, 5 | Wall bleeding hind | Ear score | GG | GA | AA | 6 | 64 | 166 | 0.17 | 1.88 | 2.65 | 0.17 | 1.60 | 0.07 | A | G | 20.1 | 11.4 |
| 1 | 253523847 | 1, 8 | Teat necrosis | Ear score | TT | TC | CC | 15 | 63 | 157 | 0.93 | 1.95 | 2.68 | 0.27 | 1.67 | 0.08 | C | T | 19.9 | 11.2 |
| 10 | 49609959 | 10, 2 | Wall bleeding hind | Ears shiny skin | GG | GA | AA | 4 | 58 | 174 | 0.00 | 0.47 | 0.86 | 0.00 | 0.33 | 0.03 | A | G | 19.9 | 11.2 |
| 14 | 91808934 | 14, 2 | Wall bleeding hind | Ears shiny skin | TT | CT | CC | 172 | 58 | 6 | 0.85 | 0.50 | 0.00 | 0.03 | 0.37 | 0.00 | T | C | 19.8 | 11.2 |
| 12 | 44738423 | 12, 5 | Heels bleeding hind | SINS | TT | TC | CC | 39 | 132 | 56 | 11.72 | 15.99 | 18.68 | 4.98 | 4.13 | 4.97 | C | T | 19.7 | 10.7 |
| 10 | 3028232 | 10, 1 | Tail tip score | Tail base score | TT | CT | CC | 3 | 67 | 166 | 1.67 | 1.93 | 0.81 | 0.88 | 1.65 | 0.08 | T | C | 19.7 | 11.1 |
| 13 | 13362832 | 13, 2 | Teat necrosis | SINS | TT | TC | CC | 150 | 71 | 6 | 14.35 | 18.86 | 20.50 | 4.11 | 5.30 | 3.51 | C | T | 19.7 | 10.6 |
| 1 | 31190769 | 1, 2 | Tail swelling | Tail no bristles | AA | CA | CC | 3 | 67 | 166 | 1.00 | 0.16 | 0.03 | 0.00 | 0.07 | 0.01 | A | C | 19.6 | 11.0 |
| 13 | 34980722 | 13, 4 | Wall bleeding hind | Wall bleeding hind | TT | CT | CC | 2 | 24 | 206 | 0.00 | 0.50 | 0.92 | 0.00 | 0.28 | 0.02 | C | T | 19.6 | 10.8 |
| 8 | 1208125 | 8, 1 | Wall bleeding hind | Wall bleeding hind | GG | GA | AA | 210 | 21 | 1 | 0.91 | 0.43 | 0.00 | 0.02 | 0.20 | 0.00 | G | A | 19.6 | 10.8 |
| 15 | 27275926 | 15, 1 | Ear score | Ear score | GG | GA | AA | 145 | 76 | 13 | 2.03 | 2.79 | 3.77 | 0.09 | 2.59 | 0.20 | A | G | 19.5 | 10.9 |
| 16 | 44669358 | 16, 2 | Heels bleeding hind | SINS | GG | AG | AA | 11 | 50 | 165 | 6.64 | 15.16 | 16.78 | 4.27 | 3.27 | 4.84 | A | G | 19.5 | 10.5 |
| 9 | 90241577 | 9, 2 | Wall bleeding front | Ears shiny skin | TT | CT | CC | 170 | 59 | 7 | 0.85 | 0.53 | 0.00 | 0.03 | 0.39 | 0.00 | T | C | 19.5 | 11.0 |
| 17 | 57531936 | 17, 4 | Wall score | Wall score | TT | TC | CC | 20 | 108 | 103 | 0.85 | 1.76 | 1.86 | 0.22 | 1.66 | 0.05 | C | T | 19.4 | 10.7 |
| 1 | 253523847 | 1, 8 | Teat necrosis | Ears shiny skin | TT | TC | CC | 15 | 63 | 157 | 0.20 | 0.57 | 0.87 | 0.11 | 0.45 | 0.03 | C | T | 19.4 | 10.9 |
| 10 | 49785901 | 10, 2 | Wall bleeding hind | Ear score | GG | AG | AA | 4 | 51 | 179 | 0.00 | 1.73 | 2.62 | 0.00 | 1.41 | 0.07 | A | G | 19.2 | 10.7 |
| 1 | 35812331 | 1, 3 | Tail tip score | Tail tip score | AA | AC | CC | 212 | 23 | 1 | 0.56 | 1.78 | 5.00 | 0.06 | 1.20 | 0.00 | C | A | 19.1 | 10.7 |
| 15 | 75578605 | 15, 2 | SINS | Ears shiny skin | GG | GC | CC | 177 | 56 | 3 | 0.84 | 0.48 | 0.00 | 0.03 | 0.35 | 0.00 | G | C | 19.0 | 10.7 |
| 16 | 47984548 | 16, 2 | Nose back edema | Nose back edema | GG | GT | TT | 206 | 25 | 1 | 0.04 | 0.36 | 1.00 | 0.01 | 0.16 | 0.00 | T | G | 19.0 | 10.5 |
| 16 | 44669358 | 16, 2 | Heels bleeding hind | Ears shiny skin | GG | AG | AA | 11 | 52 | 172 | 0.09 | 0.56 | 0.85 | 0.09 | 0.42 | 0.03 | A | G | 19.0 | 10.6 |
| 12 | 14498120 | 12, 2 | Wall bleeding hind | Ear score | GG | GT | TT | 177 | 56 | 3 | 2.63 | 1.70 | 0.00 | 0.07 | 1.40 | 0.00 | G | T | 18.9 | 10.6 |
| 13 | 9300436 | 13, 1 | Wall bleeding hind | Wall bleeding front | GG | GC | CC | 214 | 17 | 4 | 0.91 | 0.41 | 0.25 | 0.02 | 0.15 | 0.25 | G | C | 18.9 | 10.6 |
| 13 | 56534476 | 13, 6 | Wall bleeding hind | Wall bleeding hind | GG | CG | CC | 211 | 18 | 3 | 0.91 | 0.50 | 0.00 | 0.02 | 0.24 | 0.00 | G | C | 18.9 | 10.4 |
| 13 | 53856072 | 13, 5 | Tail base score | Ears no bristles | TT | CT | CC | 74 | 116 | 46 | 0.77 | 0.75 | 0.24 | 0.05 | 0.67 | 0.06 | T | C | 18.8 | 10.5 |
| 11 | 70939109 | 11, 5 | Wall bleeding hind | Ears shiny skin | TT | CT | CC | 4 | 57 | 175 | 0.00 | 0.47 | 0.85 | 0.00 | 0.34 | 0.03 | C | T | 18.8 | 10.5 |
| 12 | 14407795 | 12, 1 | Wall bleeding hind | Ear score | GG | GA | AA | 176 | 56 | 4 | 2.65 | 1.64 | 0.75 | 0.07 | 1.34 | 0.75 | G | A | 18.7 | 10.5 |
| 13 | 56534476 | 13, 6 | Wall bleeding hind | Wall score | GG | CG | CC | 210 | 18 | 3 | 1.81 | 1.00 | 0.33 | 0.04 | 0.55 | 0.33 | G | C | 18.7 | 10.2 |
| 15 | 76941405 | 15, 4 | Ears shiny skin | SINS | GG | GC | CC | 7 | 57 | 163 | 6.00 | 14.14 | 16.97 | 3.56 | 4.52 | 4.57 | C | G | 18.6 | 10.0 |
| 15 | 76941405 | 15, 4 | Ear score | SINS | GG | GC | CC | 7 | 57 | 163 | 6.00 | 14.14 | 16.97 | 3.56 | 4.52 | 4.57 | C | G | 18.6 | 10.0 |
| 15 | 27275926 | 15, 1 | Ear score | Tail base swelling | GG | GA | AA | 145 | 76 | 13 | 0.25 | 0.68 | 0.69 | 0.04 | 0.58 | 0.13 | A | G | 18.6 | 10.3 |
| 6 | 18668130 | 6, 2 | Face score | SINS | GG | AG | AA | 6 | 60 | 160 | 21.67 | 18.93 | 14.55 | 6.02 | 5.10 | 4.25 | G | A | 18.5 | 9.9 |
| 2 | 140645128 | 2, 5 | Wall bleeding hind | Ears no bristles | TT | TC | CC | 59 | 139 | 38 | 0.31 | 0.78 | 0.74 | 0.06 | 0.71 | 0.07 | C | T | 18.4 | 10.3 |
| 12 | 34198370 | 12, 4 | Tail tip cabs | Tail tip scabs | TT | TC | CC | 90 | 111 | 34 | 0.11 | 0.31 | 0.71 | 0.03 | 0.22 | 0.08 | C | T | 18.2 | 10.1 |
| 13 | 13362832 | 13, 2 | Teat necrosis | Ears no bristles | TT | TC | CC | 179 | 54 | 3 | 0.77 | 0.30 | 0.33 | 0.03 | 0.17 | 0.33 | T | C | 18.2 | 10.1 |
| 16 | 18970172 | 16, 1 | Wall score | Wall bleeding front | GG | AG | AA | 10 | 41 | 182 | 0.50 | 0.61 | 0.94 | 0.17 | 0.45 | 0.02 | A | G | 18.2 | 10.0 |
| 1 | 212887602 | 1, 6 | SINS | Ears shiny skin | AA | AC | CC | 8 | 55 | 172 | 0.25 | 0.47 | 0.85 | 0.16 | 0.34 | 0.03 | C | A | 18.1 | 10.1 |
| 13 | 53856072 | 13, 5 | Tail base score | Ears shiny skin | TT | CT | CC | 74 | 116 | 46 | 0.85 | 0.83 | 0.37 | 0.04 | 0.76 | 0.07 | T | C | 18.1 | 10.1 |
| 17 | 57531936 | 17, 4 | Wall score | Wall bleeding hind | TT | TC | CC | 20 | 108 | 104 | 0.40 | 0.89 | 0.93 | 0.11 | 0.83 | 0.02 | C | T | 18.1 | 9.9 |
| 5 | 85068157 | 5, 3 | Face score | SINS | TT | CT | CC | 3 | 64 | 160 | 20.00 | 19.16 | 14.55 | 0.00 | 5.28 | 4.25 | T | C | 18.1 | 9.7 |
| 14 | 91808934 | 14, 2 | Wall bleeding hind | SINS | TT | CT | CC | 165 | 56 | 6 | 16.85 | 14.36 | 4.83 | 4.63 | 4.45 | 1.94 | T | C | 18.0 | 9.7 |
| 1 | 66905225 | 1, 5 | Wall bleeding hind | Wall bleeding hind | GG | GA | AA | 6 | 63 | 163 | 0.00 | 0.84 | 0.91 | 0.00 | 0.75 | 0.02 | A | G | 18.0 | 9.8 |
| 17 | 40157128 | 17, 3 | Wall score | Ears shiny skin | GG | GA | AA | 172 | 56 | 7 | 0.84 | 0.54 | 0.00 | 0.03 | 0.40 | 0.00 | G | A | 17.9 | 9.9 |
| 15 | 76941405 | 15, 4 | Ears shiny skin | Wall bleeding hind | GG | GA | AA | 173 | 51 | 6 | 0.87 | 0.94 | 0.00 | 0.03 | 0.87 | 0.00 | G | A | 17.9 | 9.7 |
| 15 | 76941405 | 15, 4 | Ears shiny skin | Ears shiny skin | GG | GA | AA | 176 | 52 | 6 | 0.85 | 0.52 | 0.00 | 0.03 | 0.38 | 0.00 | G | A | 17.8 | 9.9 |
| 11 | 71239679 | 11, 6 | Ears shiny skin | WallEinblH | TT | CT | CC | 6 | 50 | 175 | 0.00 | 0.94 | 0.87 | 0.00 | 0.87 | 0.03 | C | T | 17.8 | 9.7 |
| 15 | 75578605 | 15, 2 | SINS | SINS | GG | GC | CC | 6 | 54 | 167 | 4.83 | 14.35 | 16.83 | 1.94 | 4.49 | 4.63 | C | G | 17.8 | 9.6 |
| 11 | 71239679 | 11, 6 | Ears shiny skin | Ears shiny skin | TT | CT | CC | 6 | 51 | 178 | 0.00 | 0.51 | 0.84 | 0.00 | 0.37 | 0.03 | C | T | 17.8 | 9.8 |
| 12 | 14498120 | 12, 2 | Wall bleeding hind | Ears shiny skin | GG | GT | TT | 177 | 56 | 3 | 0.85 | 0.46 | 0.00 | 0.03 | 0.33 | 0.00 | G | T | 17.7 | 9.9 |
| 13 | 13362832 | 13, 2 | Teat necrosis | Ear score | TT | TC | CC | 179 | 54 | 3 | 2.63 | 1.67 | 0.33 | 0.07 | 1.35 | 0.33 | T | C | 17.7 | 9.8 |
| 7 | 49086851 | 7, 3 | Face score | Ears no bristles | GG | AG | AA | 176 | 59 | 1 | 0.77 | 0.32 | 0.00 | 0.03 | 0.20 | 0.00 | G | A | 17.7 | 9.8 |
| 14 | 75918198 | 14, 1 | Tail swelling | Wall bleeding hind | GG | AG | AA | 168 | 57 | 6 | 0.88 | 0.93 | 0.00 | 0.03 | 0.86 | 0.00 | G | A | 17.7 | 9.6 |
| 13 | 53856072 | 13, 4 | Tail base score | Ear score | TT | CT | CC | 74 | 116 | 46 | 2.58 | 2.62 | 1.43 | 0.11 | 2.43 | 0.17 | T | C | 17.7 | 9.8 |
| 14 | 100186814 | 14, 3 | Teat necrosis | Ear score | TT | TC | CC | 3 | 60 | 173 | 0.33 | 1.72 | 2.64 | 0.33 | 1.42 | 0.07 | C | T | 17.7 | 9.8 |
| 2 | 140645128 | 2, 5 | Wall bleeding hind | Ear score | TT | TC | CC | 59 | 139 | 38 | 1.58 | 2.66 | 2.58 | 0.15 | 2.50 | 0.16 | C | T | 17.6 | 9.8 |
| 11 | 70939109 | 11, 5 | Wall bleeding hind | Ears no bristles | TT | CT | CC | 4 | 57 | 175 | 0.00 | 0.35 | 0.77 | 0.00 | 0.22 | 0.03 | C | T | 17.6 | 9.8 |
| 1 | 31190769 | 1, 2 | Tail swelling | SINS | AA | CA | CC | 2 | 64 | 161 | 23.50 | 18.98 | 14.61 | 2.12 | 5.24 | 4.28 | A | C | 17.6 | 9.4 |
| 14 | 75918198 | 14, 1 | Tail swelling | Ears shiny skin | GG | AG | AA | 171 | 58 | 6 | 0.85 | 0.53 | 0.00 | 0.03 | 0.40 | 0.00 | G | A | 17.4 | 9.7 |
| 1 | 66905225 | 1, 5 | Wall bleeding hind | SINS | GG | GA | AA | 6 | 62 | 159 | 4.83 | 14.60 | 16.86 | 1.94 | 4.03 | 4.81 | A | G | 17.4 | 9.3 |
| 10 | 49785901 | 10, 2 | Wall bleeding hind | Ears no bristles | GG | AG | AA | 4 | 51 | 179 | 0.00 | 0.33 | 0.77 | 0.00 | 0.20 | 0.03 | A | G | 17.4 | 9.6 |
| 12 | 50607044 | 12, 6 | Ear score | Ear score | GG | GT | TT | 22 | 110 | 104 | 1.27 | 2.19 | 2.81 | 0.26 | 1.98 | 0.08 | T | G | 17.4 | 9.7 |
| 13 | 208309290 | 13, 8 | Wall bleeding front | Wall bleeding hind | GG | GA | AA | 201 | 30 | 1 | 0.92 | 0.53 | 0.00 | 0.02 | 0.34 | 0.00 | G | A | 17.4 | 9.5 |
| 12 | 44738423 | 12, 5 | Heels bleeding hind | Heels bleeding hind | TT | TC | CC | 41 | 136 | 59 | 1.07 | 1.86 | 2.07 | 0.11 | 1.73 | 0.09 | C | T | 17.4 | 9.7 |
| 15 | 76941405 | 15, 4 | Ears shiny skin | Wall score | GG | GA | AA | 172 | 51 | 6 | 1.74 | 1.86 | 0.17 | 0.05 | 1.74 | 0.17 | G | A | 17.4 | 9.4 |
| 4 | 106109743 | 4, 1 | Nose back edema | SINS | GG | GA | AA | 163 | 61 | 2 | 14.62 | 19.21 | 20.50 | 4.25 | 5.39 | 0.71 | A | G | 17.3 | 9.2 |
| 17 | 30666157 | 17, 2 | Wall bleeding front | Wall score | TT | CT | CC | 1 | 28 | 202 | 0.00 | 1.11 | 1.82 | 0.00 | 0.80 | 0.04 | C | T | 17.3 | 9.4 |
| 17 | 30666157 | 17, 2 | Wall score | Wall score | TT | CT | CC | 1 | 28 | 202 | 0.00 | 1.11 | 1.82 | 0.00 | 0.80 | 0.04 | C | T | 17.3 | 9.4 |
| 1 | 212887602 | 1, 6 | SINS | SINS | AA | AC | CC | 7 | 54 | 165 | 6.00 | 14.33 | 16.88 | 3.56 | 4.49 | 4.63 | C | A | 17.3 | 9.2 |
| 14 | 91808934 | 14, 2 | Wall bleeding hind | Wall bleeding hind | TT | CT | CC | 169 | 57 | 6 | 0.89 | 0.88 | 0.00 | 0.02 | 0.79 | 0.00 | T | C | 17.3 | 9.4 |
| 15 | 75578605 | 15, 2 | SINS | Wall bleeding hind | GG | GC | CC | 174 | 55 | 3 | 0.88 | 0.87 | 0.00 | 0.02 | 0.78 | 0.00 | G | C | 17.2 | 9.4 |
| 13 | 148170559 | 13, 7 | Tail base redness | Tail base score | TT | TC | CC | 76 | 118 | 42 | 0.61 | 1.17 | 2.00 | 0.11 | 0.97 | 0.17 | C | T | 17.2 | 9.5 |
| 1 | 66905225 | 1, 5 | Wall bleeding hind | Wall score | GG | GA | AA | 6 | 63 | 162 | 0.17 | 1.70 | 1.80 | 0.17 | 1.54 | 0.04 | A | G | 17.2 | 9.3 |
| 14 | 75918198 | 14, 1 | Tail swelling | Wall score | GG | AG | AA | 167 | 57 | 6 | 1.74 | 1.84 | 0.17 | 0.05 | 1.71 | 0.17 | G | A | 17.2 | 9.3 |
| 1 | 66905225 | 1, 5 | Wall bleeding hind | Ears shiny skin | GG | GA | AA | 6 | 64 | 166 | 0.00 | 0.55 | 0.85 | 0.00 | 0.42 | 0.03 | A | G | 17.1 | 9.5 |
| 11 | 71239679 | 11, 6 | Ears shiny skin | Wall score | TT | CT | CC | 6 | 50 | 174 | 0.17 | 1.84 | 1.75 | 0.17 | 1.70 | 0.04 | C | T | 17.1 | 9.2 |
| 16 | 62806092 | 16, 4 | Ears vein congestion | Ears vein congestion | TT | CT | CC | 6 | 54 | 176 | 1.50 | 1.31 | 0.85 | 0.22 | 1.17 | 0.03 | T | C | 17.0 | 9.5 |
| 9 | 90241577 | 9, 2 | Wall bleeding front | Ears no bristles | TT | CT | CC | 170 | 59 | 7 | 0.78 | 0.37 | 0.14 | 0.03 | 0.25 | 0.14 | T | C | 17.0 | 9.4 |
| 15 | 27275926 | 15, 1 | Ear score | Ears vein congestion | GG | GA | AA | 145 | 76 | 13 | 0.86 | 1.05 | 1.77 | 0.04 | 0.94 | 0.20 | A | G | 17.0 | 9.3 |
| 12 | 15058247 | 12, 3 | Coronary bands front | Tail base no bristles | TT | CT | CC | 26 | 124 | 86 | 0.85 | 0.36 | 0.17 | 0.07 | 0.28 | 0.04 | T | C | 17.0 | 9.4 |
| 15 | 75578605 | 15, 2 | SINS | Ears no bristles | GG | GC | CC | 177 | 56 | 3 | 0.77 | 0.32 | 0.00 | 0.03 | 0.20 | 0.00 | G | C | 16.9 | 9.4 |
| 7 | 26025361 | 7, 2 | Tail swelling | Tail base no bristles | GG | CG | CC | 2 | 40 | 192 | 1.00 | 0.75 | 0.26 | 0.00 | 0.61 | 0.03 | G | C | 16.9 | 9.3 |
| 16 | 63636863 | 16, 5 | Ears shiny skin | Ears no bristles | AA | AT | TT | 164 | 63 | 9 | 0.77 | 0.44 | 0.00 | 0.03 | 0.32 | 0.00 | A | T | 16.9 | 9.4 |
| 1 | 212887602 | 1, 6 | SINS | Ears no bristles | AA | AC | CC | 8 | 55 | 172 | 0.38 | 0.33 | 0.77 | 0.18 | 0.20 | 0.03 | C | A | 16.8 | 9.3 |
| 10 | 3028232 | 10, 1 | Tail tip score | SINS | GG | GA | AA | 166 | 58 | 3 | 14.73 | 18.91 | 24.00 | 4.38 | 5.09 | 5.29 | A | G | 16.8 | 9.0 |
| 16 | 63636863 | 16, 5 | Ears shiny skin | Ear score | AA | AT | TT | 164 | 63 | 9 | 2.65 | 1.87 | 0.89 | 0.08 | 1.60 | 0.20 | A | T | 16.8 | 9.3 |
| 15 | 76926106 | 15, 3 | Wall bleeding hind | Teats vein congestion | GG | GC | CC | 7 | 58 | 171 | 0.14 | 0.12 | 0.58 | 0.14 | 0.03 | 0.04 | C | G | 16.7 | 9.3 |
| 15 | 76941405 | 15, 4 | Ear score | Teats vein congestion | GG | GC | CC | 7 | 58 | 171 | 0.14 | 0.12 | 0.58 | 0.14 | 0.03 | 0.04 | C | G | 16.7 | 9.3 |
| 18 | 1374220 | 18, 1 | Tail tip cabs | Tail tip scabs | GG | AG | AA | 3 | 19 | 214 | 1.00 | 0.84 | 0.23 | 0.00 | 0.66 | 0.03 | G | A | 16.7 | 9.3 |
| 14 | 91808934 | 14, 2 | Wall bleeding hind | Wall score | TT | CT | CC | 168 | 57 | 6 | 1.77 | 1.75 | 0.17 | 0.04 | 1.59 | 0.17 | T | C | 16.7 | 9.1 |
| 15 | 75578605 | 15, 2 | SINS | Wall score | GG | GC | CC | 173 | 55 | 3 | 1.75 | 1.76 | 0.00 | 0.05 | 1.60 | 0.00 | G | C | 16.7 | 9.0 |
| 17 | 30666157 | 17, 2 | Wall bleeding front | Wall bleeding front | TT | CT | CC | 1 | 29 | 205 | 0.00 | 0.52 | 0.91 | 0.00 | 0.32 | 0.02 | C | T | 16.6 | 9.1 |
| 17 | 30666157 | 17, 2 | Wall score | Wall bleeding front | TT | CT | CC | 1 | 29 | 205 | 0.00 | 0.52 | 0.91 | 0.00 | 0.32 | 0.02 | C | T | 16.6 | 9.1 |
| 14 | 91808934 | 14, 2 | Wall bleeding hind | Ears no bristles | TT | CT | CC | 172 | 58 | 6 | 0.77 | 0.36 | 0.17 | 0.03 | 0.23 | 0.17 | T | C | 16.6 | 9.2 |
| 9 | 90241577 | 9, 2 | Wall bleeding front | SINS | TT | CT | CC | 163 | 57 | 7 | 16.80 | 14.63 | 5.86 | 4.77 | 4.18 | 3.24 | T | C | 16.5 | 8.8 |
| 16 | 44669358 | 16, 2 | Heels bleeding hind | Ears no bristles | GG | AG | AA | 11 | 52 | 172 | 0.18 | 0.38 | 0.77 | 0.12 | 0.25 | 0.03 | A | G | 16.4 | 9.0 |
| 7 | 26025361 | 7, 2 | Tail swelling | SINS | GG | CG | CC | 2 | 38 | 185 | 26.50 | 19.74 | 15.03 | 4.95 | 4.84 | 4.56 | G | C | 16.4 | 8.6 |
| 18 | 8384320 | 18, 3 | Tail base redness | Tail base score | TT | CT | CC | 72 | 104 | 60 | 0.64 | 1.06 | 1.87 | 0.11 | 0.85 | 0.15 | C | T | 16.4 | 9.1 |
| 2 | 15794455 | 2, 2 | Wall score | Wall score | TT | TC | CC | 5 | 88 | 138 | 1.00 | 1.45 | 1.93 | 0.45 | 1.28 | 0.03 | C | T | 16.4 | 8.9 |
| 17 | 30666157 | 17, 2 | Wall bleeding front | Tail tip scabs | TT | CT | CC | 1 | 29 | 206 | 1.00 | 0.76 | 0.22 | 0.00 | 0.59 | 0.03 | T | C | 16.4 | 9.0 |
| 17 | 30666157 | 17, 2 | Wall score | Tail tip scabs | TT | CT | CC | 1 | 29 | 206 | 1.00 | 0.76 | 0.22 | 0.00 | 0.59 | 0.03 | T | C | 16.4 | 9.0 |
| 1 | 271201482 | 1, 9 | Tail base score | Ear score | GG | GA | AA | 14 | 104 | 118 | 1.79 | 1.95 | 2.82 | 0.33 | 1.73 | 0.08 | A | G | 16.4 | 9.0 |
| 11 | 71239679 | 11, 6 | Ears shiny skin | SINS | TT | TC | CC | 171 | 49 | 6 | 16.70 | 14.63 | 4.83 | 4.77 | 4.18 | 1.94 | T | C | 16.3 | 8.6 |
| 13 | 148170559 | 13, 7 | Tail base redness | Ear score | TT | TC | CC | 76 | 118 | 42 | 1.74 | 2.64 | 2.81 | 0.14 | 2.45 | 0.10 | C | T | 16.3 | 9.0 |
| 7 | 49086851 | 7, 3 | Face score | Ear score | GG | AG | AA | 176 | 59 | 1 | 2.64 | 1.61 | 2.00 | 0.07 | 1.30 | 0.00 | G | A | 16.2 | 9.0 |
| 2 | 8270586 | 2, 1 | Ears vein congestion | Ears vein congestion | GG | GA | AA | 211 | 23 | 2 | 0.93 | 1.22 | 3.00 | 0.03 | 0.99 | 0.00 | A | G | 16.2 | 8.9 |
| 12 | 14407795 | 12, 1 | Wall bleeding hind | Ears shiny skin | GG | GA | AA | 176 | 56 | 4 | 0.85 | 0.46 | 0.25 | 0.03 | 0.33 | 0.25 | G | A | 16.1 | 8.9 |
| 6 | 10345325 | 6, 1 | Wall bleeding hind | SINS | GG | AG | AA | 6 | 61 | 160 | 18.00 | 19.10 | 14.63 | 6.87 | 5.15 | 4.28 |  |  | 16.1 | 8.5 |
| 11 | 16097396 | 11, 2 | Ears shiny skin | Ears shiny skin | GG | AG | AA | 204 | 30 | 2 | 0.81 | 0.33 | 0.00 | 0.03 | 0.15 | 0.00 | G | A | 16.0 | 8.8 |
| 1 | 253523847 | 1, 8 | Teat necrosis | Ears no bristles | TT | TC | CC | 15 | 63 | 157 | 0.20 | 0.44 | 0.78 | 0.11 | 0.32 | 0.03 | C | T | 16.0 | 8.8 |
| 16 | 44669358 | 16, 2 | Heels bleeding hind | Teats vein congestion | GG | AG | AA | 11 | 52 | 172 | 0.09 | 0.13 | 0.58 | 0.09 | 0.04 | 0.04 | A | G | 16.0 | 8.8 |
| 12 | 14407795 | 12, 1 | Wall bleeding hind | Ears no bristles | GG | GA | AA | 176 | 56 | 4 | 0.77 | 0.34 | 0.25 | 0.03 | 0.21 | 0.25 | G | A | 15.9 | 8.7 |
| 11 | 21453639 | 11, 4 | Coronary bands score | SINS | TT | CT | CC | 178 | 41 | 7 | 16.64 | 14.61 | 5.71 | 4.77 | 4.10 | 2.93 | T | C | 15.8 | 8.3 |
| 7 | 21333865 | 7, 1 | Tail tip cabs | Tail tip scabs | GG | GA | AA | 208 | 24 | 3 | 0.22 | 0.75 | 1.00 | 0.03 | 0.56 | 0.00 | A | G | 15.8 | 8.7 |
| 9 | 90241577 | 9, 2 | Wall bleeding front | Teats vein congestion | TT | CT | CC | 170 | 59 | 7 | 0.58 | 0.14 | 0.14 | 0.04 | 0.05 | 0.14 | T | C | 15.7 | 8.7 |
| 17 | 40157128 | 17, 3 | Wall score | SINS | GG | GA | AA | 167 | 52 | 7 | 16.77 | 14.56 | 6.14 | 4.79 | 4.11 | 3.89 | G | A | 15.7 | 8.3 |
| 12 | 14407795 | 12, 1 | Wall bleeding hind | Teats vein congestion | GG | GA | AA | 176 | 56 | 4 | 0.57 | 0.13 | 0.00 | 0.04 | 0.04 | 0.00 | G | A | 15.7 | 8.6 |
| 13 | 13362832 | 13, 2 | Teat necrosis | Teats vein congestion | TT | TC | CC | 179 | 54 | 3 | 0.56 | 0.11 | 0.00 | 0.04 | 0.02 | 0.00 | T | C | 15.6 | 8.6 |
| 12 | 14498120 | 12, 2 | Wall bleeding hind | Ears no bristles | GG | GT | TT | 177 | 56 | 3 | 0.76 | 0.36 | 0.00 | 0.03 | 0.23 | 0.00 | G | T | 15.6 | 8.6 |
| 13 | 13362832 | 13, 2 | Teat necrosis | Ears shiny skin | TT | TC | CC | 179 | 54 | 3 | 0.84 | 0.48 | 0.00 | 0.03 | 0.34 | 0.00 | T | C | 15.6 | 8.6 |
| 14 | 75918198 | 14, 1 | Tail swelling | SINS | GG | AG | AA | 164 | 56 | 6 | 16.66 | 14.98 | 4.83 | 4.78 | 4.31 | 1.94 | G | A | 15.6 | 8.2 |
| 14 | 100186814 | 14, 3 | Teat necrosis | Ears shiny skin | TT | TC | CC | 3 | 60 | 173 | 0.00 | 0.50 | 0.84 | 0.00 | 0.37 | 0.03 | C | T | 15.6 | 8.6 |
| 7 | 26025361 | 7, 2 | Tail swelling | Tail base swelling | GG | CG | CC | 2 | 40 | 192 | 1.00 | 0.83 | 0.33 | 0.00 | 0.70 | 0.03 | G | C | 15.5 | 8.5 |
| 1 | 212887602 | 1, 6 | SINS | Teats vein congestion | AA | AC | CC | 8 | 55 | 172 | 0.13 | 0.13 | 0.57 | 0.13 | 0.04 | 0.04 | C | A | 15.5 | 8.5 |
| 11 | 71239679 | 11, 6 | Ears shiny skin | Ears no bristles | TT | CT | CC | 6 | 51 | 178 | 0.17 | 0.35 | 0.76 | 0.17 | 0.22 | 0.03 | C | T | 15.5 | 8.5 |
| 16 | 18970172 | 16, 1 | Wall score | Wall bleeding hind | GG | AG | AA | 10 | 41 | 179 | 0.50 | 0.66 | 0.94 | 0.17 | 0.51 | 0.02 | A | G | 15.5 | 8.3 |
| 14 | 75918198 | 14, 1 | Tail swelling | Ears no bristles | GG | AG | AA | 171 | 58 | 6 | 0.77 | 0.38 | 0.17 | 0.03 | 0.25 | 0.17 | G | A | 15.5 | 8.5 |
| 1 | 14157691 | 1, 1 | Tail swelling | Tail no bristles | AA | CA | CC | 2 | 35 | 198 | 1.00 | 0.23 | 0.05 | 0.00 | 0.08 | 0.01 | A | C | 15.5 | 8.5 |
| 12 | 50607044 | 12, 6 | Ear score | Ears shiny skin | GG | GT | TT | 22 | 110 | 104 | 0.41 | 0.65 | 0.92 | 0.11 | 0.55 | 0.03 | T | G | 15.4 | 8.4 |
| 15 | 76926106 | 15, 3 | Wall bleeding hind | SINS | GG | GA | AA | 169 | 50 | 6 | 16.63 | 15.04 | 4.83 | 4.95 | 3.53 | 1.94 | G | A | 15.3 | 8.0 |
| 11 | 21365824 | 11, 3 | Coronary bands front | Ear score | AA | CA | CC | 3 | 41 | 192 | 0.33 | 1.61 | 2.57 | 0.33 | 1.24 | 0.07 | C | A | 15.3 | 8.4 |
| 14 | 100186814 | 14, 3 | Teat necrosis | Ears no bristles | TT | TC | CC | 3 | 60 | 173 | 0.33 | 0.35 | 0.77 | 0.33 | 0.23 | 0.03 | C | T | 15.3 | 8.4 |
| 15 | 76941405 | 15, 4 | Ears shiny skin | Ears no bristles | GG | GA | AA | 176 | 52 | 6 | 0.77 | 0.37 | 0.17 | 0.03 | 0.23 | 0.17 | G | A | 15.3 | 8.3 |
| 12 | 14498120 | 12, 2 | Wall bleeding hind | Teats vein congestion | GG | GT | TT | 177 | 56 | 3 | 0.56 | 0.13 | 0.00 | 0.04 | 0.04 | 0.00 | G | T | 15.1 | 8.3 |
| 17 | 7734948 | 17, 1 | Tail base score | Tail base score | GG | TG | TT | 2 | 115 | 119 | 0.00 | 0.70 | 1.57 | 0.00 | 0.54 | 0.11 | T | G | 15.1 | 8.3 |
| 3 | 22988098 | 3, 1 | Wall bleeding front | Wall bleeding front | GG | GA | AA | 16 | 101 | 116 | 0.63 | 0.74 | 0.99 | 0.13 | 0.66 | 0.01 | A | G | 15.1 | 8.2 |
| 3 | 22988098 | 3, 1 | Wall score | Wall bleeding front | GG | GA | AA | 16 | 101 | 116 | 0.63 | 0.74 | 0.99 | 0.13 | 0.66 | 0.01 | A | G | 15.1 | 8.2 |
| 2 | 104384195 | 2, 4 | Ears no bristles | Ears no bristles | GG | GA | AA | 141 | 81 | 14 | 0.78 | 0.54 | 0.07 | 0.04 | 0.43 | 0.07 | G | A | 15.0 | 8.2 |
| 18 | 7597561 | 18, 2 | Tail base score | Tail base swelling | TT | TC | CC | 53 | 113 | 70 | 0.68 | 0.46 | 0.16 | 0.06 | 0.37 | 0.04 | T | C | 14.9 | 8.2 |
| 9 | 5511145 | 9, 1 | Wall bleeding hind | Ear score | AA | AT | TT | 170 | 65 | 1 | 2.64 | 1.71 | 1.00 | 0.07 | 1.41 | 0.00 | A | T | 14.9 | 8.2 |
| 11 | 21365824 | 11, 3 | Coronary bands front | Ears shiny skin | AA | CA | CC | 3 | 41 | 192 | 0.00 | 0.44 | 0.82 | 0.00 | 0.28 | 0.03 | C | A | 14.9 | 8.2 |
| 13 | 148170559 | 13, 7 | Tail base redness | Ears no bristles | TT | TC | CC | 76 | 118 | 42 | 0.39 | 0.76 | 0.83 | 0.06 | 0.68 | 0.06 | C | T | 14.8 | 8.1 |
| 13 | 148170559 | 13, 7 | Tail base redness | Ears shiny skin | TT | TC | CC | 76 | 118 | 42 | 0.51 | 0.82 | 0.95 | 0.06 | 0.75 | 0.03 | C | T | 14.7 | 8.1 |
| 1 | 243296440 | 1, 7 | Ear score | Ear score | GG | AG | AA | 47 | 108 | 81 | 2.89 | 2.57 | 1.81 | 0.14 | 2.40 | 0.13 | G | A | 14.7 | 8.1 |
| X | 32069776 | X, 1 | Tail base swelling | Tail base score | GG | GA | AA | 7 | 43 | 184 | 2.00 | 2.02 | 0.90 | 0.82 | 1.12 | 1.04 | G | A | 16.2 | 8.9 |
| X | 122362632 | X, 2 | Tail bleeding | SINS | GG | GA | AA | 184 | 34 | 7 | 14.9 | 20.4 | 19.6 | 4.29 | 5.87 | 4.58 | A | G | 17.2 | 9.1 |

**Supplemental Table 6**  Positional candidate genes up to 1 Mbp apart from significant SNPs

| Gene symbol | Accession No | NCBI Gene ID | SSC | Start | Stop | Distance from SNP | GeneCards rank |
| --- | --- | --- | --- | --- | --- | --- | --- |
| TRIM68 | NC_010451.4 | 100738170 | 9 | 5502931 | 5516936 | -1212 | 17983 |
| MT1A | NC_010448.4 | 397417 | 6 | 18672016 | 18673673 | 4715 | 12149 |
| SCNN1G | NC_010445.4 | 100522244 | 3 | 22977940 | 23013384 | 7564 | 6201 |
| F2 | NC_010444.4 | 100144442 | 2 | 15793257 | 15819151 | 11749 | 3098 |
| SAMHD1 | NC_010459.5 | 100154500 | 17 | 40117348 | 40171935 | -12487 | 30804 |
| TRIM33 | NC_010446.5 | 100516192 | 4 | 106058197 | 106195145 | 16928 | 10095 |
| FAH | NC_010449.5 | 100623036 | 7 | 49047833 | 49087790 | -19040 | 15583 |
| MOBP | NC_010455.5 | 100152154 | 13 | 24138078 | 24186838 | 22433 | 22470 |
| SMURF2 | NC_010454.4 | 100627676 | 12 | 14368683 | 14492129 | 22611 | 20968 |
| CD96 | NC_010455.5 | 100627543 | 13 | 148103424 | 148192272 | -22711 | 24902 |
| CNTNAP5 | NC_010457.5 | 100622103 | 15 | 26875305 | 27740340 | 31897 | 27770 |
| MT3 | NC_010448.4 | 397123 | 6 | 18634735 | 18636134 | -32696 | 12142 |
| PTPRD | NC_010443.5 | 100627072 | 1 | 211832409 | 214013013 | 35109 | 1720 |
| TPP2 | NC_010453.5 | 100511152 | 11 | 70943142 | 71009381 | 37153 | 20548 |
| ZNF408 | NC_010444.4 | 100515769 | 2 | 15829489 | 15834549 | 37564 | 3099 |
| WNK1 | NC_010447.5 | 406235 | 5 | 68000792 | 68142533 | 37935 | 11532 |
| FOXN1 | NC_010454.4 | 100625164 | 12 | 44764427 | 44796138 | 41860 | 21632 |
| ITIH4 | NC_010455.5 | 396799 | 13 | 34928455 | 34948250 | -42370 | 22998 |
| GRIN3A | NC_010443.5 | 100153054 | 1 | 243263202 | 243416739 | 43531 | 1985 |
| CUEDC1 | NC_010454.4 | 100511955 | 12 | 34141649 | 34167731 | -43680 | 21438 |
| ARHGAP1 | NC_010444.4 | 100515942 | 2 | 15834285 | 15854181 | 49778 | 3100 |
| RTN3 | NC_010444.4 | 100512087 | 2 | 8291750 | 8354920 | 52749 | 2778 |
| RNF20 | NC_010443.5 | 100154259 | 1 | 243225711 | 243254465 | -56352 | 1982 |
| SLC46A1 | NC_010454.4 | 100628018 | 12 | 44677499 | 44686277 | -56535 | 21627 |
| ECD | NC_010456.5 | 100157830 | 14 | 75958868 | 75992622 | 57547 | 26794 |
| ATP10B | NC_010458.4 | 100626997 | 16 | 62569813 | 62918484 | -61944 | 30184 |
| ITIH3 | NC_010455.5 | 100157235 | 13 | 34911104 | 34924881 | -62730 | 22997 |
| RPSA | NC_010455.5 | 641351 | 13 | 24072551 | 24081536 | -62982 | 22463 |
| UNC119 | NC_010454.4 | 100625256 | 12 | 44801265 | 44807334 | 65877 | 21633 |
| ALOX5AP | NC_010453.5 | 397392 | 11 | 7371328 | 7438111 | -66029 | 20171 |
| MRPS23 | NC_010454.4 | 100511221 | 12 | 34123679 | 34129574 | -71744 | 21437 |
| SARM1 | NC_010454.4 | 100627920 | 12 | 44654364 | 44676942 | -72770 | 21626 |
| FBXO39 | NC_010454.4 | 106504168 | 12 | 50635915 | 50723841 | 72834 | 21813 |
| LRP2 | NC_010457.5 | 100519689 | 15 | 75565299 | 75754604 | 81347 | 28444 |
| ITIH1 | NC_010455.5 | 396963 | 13 | 34892335 | 34906378 | -81366 | 22994 |
| SRSF1 | NC_010454.4 | 654327 | 12 | 34278363 | 34282164 | 81894 | 21442 |
| RBL1 | NC_010459.5 | 100153296 | 17 | 40201214 | 40279318 | 83138 | 30811 |
| NPR3 | NC_010458.4 | 100626363 | 16 | 18841540 | 18929813 | -84496 | 29803 |
| MARK2 | NC_010444.4 | 100737238 | 2 | 8153870 | 8216141 | -85581 | 2772 |
| ZFAND6 | NC_010449.5 | 100155656 | 7 | 48967402 | 49034711 | -85795 | 15581 |
| VTN | NC_010454.4 | 397192 | 12 | 44650532 | 44653916 | -86199 | 21624 |
| SLC13A5 | NC_010454.4 | 100520976 | 12 | 50679900 | 50710985 | 88399 | 21815 |
| BMP7 | NC_010459.5 | 492315 | 17 | 57567770 | 57676516 | 90207 | 31064 |
| ATG13 | NC_010444.4 | 100516128 | 2 | 15856859 | 15916122 | 92036 | 3101 |
| DOCK2 | NC_010458.4 | 100512021 | 16 | 53843248 | 54248708 | 93003 | 30154 |
| GGT6 | NC_010454.4 | 100523568 | 12 | 50507501 | 50514578 | -96005 | 21805 |
| CST7 | NC_010459.5 | 100152012 | 17 | 30758000 | 30766472 | 96079 | 30578 |
| TMEM199 | NC_010454.4 | 100623454 | 12 | 44636379 | 44641536 | -99466 | 21621 |
| CPB2 | NC_010453.5 | 100155038 | 11 | 21234447 | 21298250 | -99476 | 20312 |
| NUP93 | NC_010448.4 | 100626040 | 6 | 18711869 | 18827705 | 101657 | 12150 |
| MYO3A | NC_010452.4 | 100515334 | 10 | 49392119 | 49623575 | -102112 | 19886 |
| SLC25A38 | NC_010455.5 | 100622220 | 13 | 24030571 | 24043866 | -102807 | 22459 |
| NEK4 | NC_010455.5 | 106504146 | 13 | 34844972 | 34909028 | -103722 | 22992 |
| POLDIP2 | NC_010454.4 | 100624252 | 12 | 44626447 | 44636302 | -107049 | 21620 |
| BBS2 | NC_010448.4 | 100622382 | 6 | 18542383 | 18575338 | -109270 | 12139 |
| ATL3 | NC_010444.4 | 100512265 | 2 | 8361579 | 8408920 | 114664 | 2779 |
| TNFAIP1 | NC_010454.4 | 100737417 | 12 | 44616840 | 44626330 | -116838 | 21619 |
| KPNA2 | NC_010454.4 | 397218 | 12 | 14283092 | 14296749 | -117875 | 20963 |
| CNR1 | NC_010443.5 | 100520160 | 1 | 56466022 | 56493275 | 118702 | 463 |
| RCOR2 | NC_010444.4 | 100526032 | 2 | 8146680 | 8153680 | -120406 | 2771 |
| SCNN1B | NC_010445.4 | 100627971 | 3 | 22832259 | 22902592 | -120673 | 6197 |
| PRKG2 | NC_010450.4 | 100512796 | 8 | 136754698 | 136854589 | 121597 | 17659 |
| IFT20 | NC_010454.4 | 100623555 | 12 | 44609586 | 44616685 | -125288 | 21618 |
| TXNDC17 | NC_010454.4 | 100521639 | 12 | 50734669 | 50737309 | 128945 | 21817 |
| CEP95 | NC_010454.4 | 100624364 | 12 | 14507841 | 14571651 | 131951 | 20973 |
| ABCB11 | NC_010457.5 | 100518154 | 15 | 75402296 | 75487092 | -133911 | 28434 |
| MOXD1 | NC_010443.5 | 100151998 | 1 | 31282491 | 31367589 | 134271 | 294 |
| SPNS2 | NC_010454.4 | 100523398 | 12 | 50454051 | 50491172 | -134433 | 21803 |
| CCR8 | NC_010455.5 | 100622393 | 13 | 23994097 | 24014849 | -135552 | 22458 |
| BRINP3 | NC_010452.4 | 100512672 | 10 | 2963237 | 3367629 | 137201 | 19420 |
| SPCS1 | NC_010455.5 | 100135675 | 13 | 34839921 | 34841605 | -139959 | 22990 |
| APAF1 | NC_010447.5 | 100513283 | 5 | 85166616 | 85258751 | 144527 | 11724 |
| TAAR1 | NC_010443.5 | 100157645 | 1 | 31037826 | 31050816 | -146448 | 278 |
| RAD52 | NC_010447.5 | 100141308 | 5 | 68140846 | 68223587 | 148489 | 11533 |
| DNAJC9 | NC_010456.5 | 100739807 | 14 | 76064598 | 76070301 | 149252 | 26796 |
| LRP4 | NC_010444.4 | 100518293 | 2 | 15616137 | 15672876 | -149949 | 3095 |
| PLA2G12B | NC_010456.5 | 100152597 | 14 | 75754525 | 75778933 | -151469 | 26786 |
| LPIN1 | NC_010445.4 | 100170845 | 3 | 124940557 | 125074137 | 151504 | 8612 |
| DSN1 | NC_010459.5 | 100152867 | 17 | 39992769 | 40017388 | -152050 | 30800 |
| PRSS2 | NC_010460.4 | 100525899 | 18 | 7443327 | 7446625 | -152585 | 31295 |
| GNL3 | NC_010455.5 | 100155235 | 13 | 34820243 | 34828523 | -156339 | 22987 |
| RNF4 | NC_010450.4 | 692189 | 8 | 1353532 | 1380637 | 158960 | 16623 |
| ALDOB | NC_010443.5 | 100515523 | 1 | 243127870 | 243141504 | -161753 | 1979 |
| LAMC3 | NC_010443.5 | 100157958 | 1 | 271004423 | 271071223 | -163659 | 2375 |
| DDX5 | NC_010454.4 | 102158045 | 12 | 14571712 | 14580740 | 168431 | 20974 |
| VNN1 | NC_010443.5 | 397246 | 1 | 31003338 | 31023731 | -177235 | 277 |
| OIT3 | NC_010456.5 | 100513952 | 14 | 75726353 | 75753461 | -178291 | 26785 |
| OTUB1 | NC_010444.4 | 100302092 | 2 | 8088528 | 8095555 | -178545 | 2768 |
| NELFA | NC_010450.4 | 100514855 | 8 | 1018443 | 1039091 | -179358 | 16614 |
| ATP7B | NC_010453.5 | 100049684 | 11 | 15892549 | 15942070 | -180087 | 20243 |
| PRRC2B | NC_010443.5 | 100511912 | 1 | 271336780 | 271430936 | 182376 | 2386 |
| POLG2 | NC_010454.4 | 110255987 | 12 | 14582075 | 14601576 | 184031 | 20975 |
| BIVM | NC_010453.5 | 100512068 | 11 | 71110463 | 71138861 | 185553 | 20553 |
| VSX1 | NC_010459.5 | 100620386 | 17 | 30848497 | 30856014 | 186099 | 30581 |
| CX3CR1 | NC_010455.5 | 100622126 | 13 | 23944386 | 23957567 | -189049 | 22452 |
| SPNS3 | NC_010454.4 | 100523208 | 12 | 50388973 | 50445297 | -189909 | 21802 |
| LCP1 | NC_010453.5 | 100156254 | 11 | 21126504 | 21223543 | -190801 | 20311 |
| G6PC2 | NC_010457.5 | 100518871 | 15 | 75382442 | 75388662 | -193053 | 28432 |
| CST3 | NC_010459.5 | 733672 | 17 | 30471056 | 30474486 | -193386 | 30567 |
| RPN2 | NC_010459.5 | 100154432 | 17 | 40322364 | 40378866 | 193487 | 30814 |
| BCL2A1 | NC_010449.5 | 100156860 | 7 | 48887227 | 48897667 | -194404 | 15580 |
| ERBIN | NC_010458.4 | 100511418 | 16 | 44415106 | 44533353 | -195129 | 30040 |
| SLC12A3 | NC_010448.4 | 110260927 | 6 | 18844752 | 18885497 | 196995 | 12151 |
| IKBIP | NC_010447.5 | 100512167 | 5 | 85258455 | 85278235 | 200188 | 11725 |
| SUB1 | NC_010458.4 | 110257216 | 16 | 18758886 | 18779421 | -201019 | 29802 |
| COG7 | NC_010445.4 | 100627695 | 3 | 22740424 | 22826589 | -204592 | 6193 |
| BMP3 | NC_010450.4 | 100512974 | 8 | 136880456 | 136904739 | 209551 | 17660 |
| PTEN | NC_010456.5 | 100156264 | 14 | 99929590 | 100021619 | -211210 | 27000 |
| HMGB1 | NC_010453.5 | 445521 | 11 | 7195556 | 7321071 | -212435 | 20167 |
| ERCC5 | NC_010453.5 | 102163752 | 11 | 71141823 | 71168326 | 215966 | 20554 |
| SLC25A3 | NC_010447.5 | 100302698 | 5 | 85284405 | 85292336 | 220214 | 11726 |
| BPTF | NC_010454.4 | 100525424 | 12 | 14112943 | 14254810 | -223919 | 20959 |
| UBE3C | NC_010460.4 | 100511906 | 18 | 1542454 | 1654274 | 224144 | 31191 |
| VNN2 | NC_010443.5 | 100153984 | 1 | 30961520 | 30970945 | -224537 | 275 |
| PBRM1 | NC_010455.5 | 100525781 | 13 | 34689718 | 34821117 | -225305 | 22984 |
| AMPD1 | NC_010446.5 | 100101472 | 4 | 105868834 | 105892171 | -229241 | 10091 |
| NSD2 | NC_010450.4 | 100515039 | 8 | 938832 | 1018032 | -229693 | 16613 |
| NEK8 | NC_010454.4 | 100514006 | 12 | 44963095 | 44975092 | 230671 | 21646 |
| AMBRA1 | NC_010444.4 | 100519700 | 2 | 15935354 | 16119082 | 232763 | 3103 |
| ALG11 | NC_010453.5 | 100152645 | 11 | 15851352 | 15876452 | -233494 | 20242 |
| LCP2 | NC_010458.4 | 100511843 | 16 | 53696974 | 53741736 | -233620 | 30149 |
| GHRH | NC_010459.5 | 100499556 | 17 | 40386725 | 40398312 | 235391 | 30815 |
| MIR146A | NC_010458.4 | 100526391 | 16 | 63044261 | 63044338 | 238208 | 30188 |
| POMT1 | NC_010443.5 | 100525803 | 1 | 271432196 | 271448584 | 238908 | 2387 |
| HERPUD1 | NC_010448.4 | 100622579 | 6 | 18901171 | 18913427 | 239169 | 12152 |
| TRAF4 | NC_010454.4 | 100518396 | 12 | 44975639 | 44981650 | 240222 | 21647 |
| PITPNM3 | NC_010454.4 | 100524106 | 12 | 50806302 | 50898316 | 245265 | 21819 |
| ESR1 | NC_010443.5 | 397435 | 1 | 14217032 | 14604906 | 253278 | 146 |
| GAD2 | NC_010452.4 | 396929 | 10 | 49316682 | 49391013 | -256112 | 19885 |
| MACROD1 | NC_010444.4 | 100522538 | 2 | 7939125 | 8087656 | -257196 | 2767 |
| TRPV6 | NC_010460.4 | 100171400 | 18 | 7332253 | 7348383 | -257243 | 31292 |
| NRAS | NC_010446.5 | 100739349 | 4 | 105845913 | 105853769 | -259902 | 10090 |
| NLK | NC_010454.4 | 100511657 | 12 | 44402990 | 44553527 | -260165 | 21615 |
| CSTL1 | NC_010459.5 | 100515284 | 17 | 30403841 | 30407571 | -260451 | 30561 |
| RBM38 | NC_010459.5 | 100125973 | 17 | 57783156 | 57802495 | 260890 | 31067 |
| NOSTRIN | NC_010457.5 | 100518697 | 15 | 75283897 | 75346901 | -263206 | 28428 |
| FIBCD1 | NC_010443.5 | 100523302 | 1 | 270919793 | 270956753 | -263209 | 2374 |
| TMPO | NC_010447.5 | 100512350 | 5 | 85317648 | 85346528 | 263931 | 11727 |
| RNLS | NC_010456.5 | 100155046 | 14 | 100312592 | 100604953 | 271959 | 27004 |
| TRIM21 | NC_010451.4 | 100302538 | 9 | 5782133 | 5790628 | 275236 | 17995 |
| HSPH1 | NC_010453.5 | 100048931 | 11 | 7733874 | 7759528 | 275953 | 20179 |
| MANBAL | NC_010459.5 | 100514259 | 17 | 40420745 | 40449657 | 278073 | 30816 |
| CXCL12 | NC_010456.5 | 494460 | 14 | 91516383 | 91543857 | -278814 | 26953 |
| HTATIP2 | NC_010444.4 | 100521603 | 2 | 39035337 | 39052178 | -279129 | 3254 |
| TRPV5 | NC_010460.4 | 100622162 | 18 | 7285186 | 7341965 | -283986 | 31291 |
| BBS5 | NC_010457.5 | 100156983 | 15 | 75850860 | 75874950 | 284300 | 28448 |
| NAPB | NC_010459.5 | 100515106 | 17 | 30361439 | 30396105 | -287385 | 30560 |
| PECAM1 | NC_010454.4 | 396941 | 12 | 14667643 | 14723296 | 287675 | 20977 |
| CCDC77 | NC_010447.5 | 100513353 | 5 | 67725757 | 67756516 | -292592 | 11527 |
| UBE2G1 | NC_010454.4 | 100522828 | 12 | 50258037 | 50368621 | -293715 | 21800 |
| EPX | NC_010454.4 | 100514365 | 12 | 34486139 | 34498879 | 294139 | 21452 |
| LETM1 | NC_010450.4 | 100514302 | 8 | 898178 | 926500 | -295786 | 16612 |
| MCU | NC_010456.5 | 100153794 | 14 | 75518533 | 75721502 | -298181 | 26784 |
| AKIRIN2 | NC_010443.5 | 100519991 | 1 | 56047188 | 56075342 | -299682 | 457 |
| CSRNP1 | NC_010455.5 | 100519275 | 13 | 23833130 | 23845198 | -300861 | 22446 |
| PYGB | NC_010459.5 | 100621536 | 17 | 30940457 | 30995688 | 301916 | 30583 |
| PTTG1 | NC_010458.4 | 397015 | 16 | 63104698 | 63112382 | 302448 | 30189 |
| AIPL1 | NC_010454.4 | 100524458 | 12 | 50903358 | 50917187 | 303229 | 21821 |
| MKS1 | NC_010454.4 | 100514538 | 12 | 34499139 | 34512191 | 307295 | 21453 |
| TGIF2 | NC_010459.5 | 110257437 | 17 | 39840649 | 39858162 | -307723 | 30795 |
| GREB1 | NC_010445.4 | 100511013 | 3 | 125099352 | 125233965 | 310816 | 8618 |
| NT5DC2 | NC_010455.5 | 100154818 | 13 | 34659249 | 34679530 | -311333 | 22978 |
| NEK3 | NC_010453.5 | 100513679 | 11 | 15772578 | 15798424 | -311895 | 20240 |
| MYRIP | NC_010455.5 | 100156583 | 13 | 24347574 | 24556743 | 312134 | 22474 |
| SLC19A1 | NC_010455.5 | 100579176 | 13 | 207986094 | 208007855 | -312316 | 25817 |
| NLRC5 | NC_010448.4 | 100135667 | 6 | 18933179 | 19036288 | 316604 | 12154 |
| PTPRN2 | NC_010460.4 | 102158899 | 18 | 721125 | 1389609 | -318853 | 31187 |
| FGFR3 | NC_010450.4 | 100514115 | 8 | 879151 | 894968 | -321066 | 16611 |
| HS3ST2 | NC_010445.4 | 100522426 | 3 | 23255936 | 23366570 | 323155 | 6205 |
| SLU7 | NC_010458.4 | 100517791 | 16 | 63118426 | 63145655 | 325949 | 30190 |
| NXT1 | NC_010459.5 | 100514739 | 17 | 30338379 | 30341629 | -326153 | 30558 |
| PRKCD | NC_010455.5 | 100622955 | 13 | 35290835 | 35323245 | 326318 | 23007 |
| TNIP2 | NC_010450.4 | 100518977 | 8 | 1530534 | 1554677 | 334481 | 16626 |
| STAB1 | NC_010455.5 | 100525609 | 13 | 34630448 | 34659371 | -335813 | 22976 |
| KEL | NC_010460.4 | 100511542 | 18 | 7242378 | 7279345 | -336700 | 31289 |
| MGAM | NC_010460.4 | 102160115 | 18 | 7871529 | 7997137 | 336772 | 31330 |
| MDK | NC_010444.4 | 100359358 | 2 | 16130787 | 16133791 | 337834 | 3106 |
| CKAP2 | NC_010453.5 | 100515274 | 11 | 15751717 | 15767068 | -338004 | 20238 |
| SRC | NC_010459.5 | 100154503 | 17 | 40470970 | 40524862 | 340788 | 30817 |
| LPO | NC_010454.4 | 100516395 | 12 | 34525113 | 34554031 | 341202 | 21454 |
| ATAD1 | NC_010456.5 | 100157471 | 14 | 99802939 | 99885562 | -342564 | 26997 |
| NECTIN3 | NC_010455.5 | 100518152 | 13 | 148448581 | 148578920 | 343192 | 24909 |
| SYT6 | NC_010446.5 | 100152816 | 4 | 106420614 | 106485468 | 343298 | 10099 |
| UBFD1 | NC_010445.4 | 100523987 | 3 | 22633492 | 22655283 | -343711 | 6187 |
| CTCFL | NC_010459.5 | 100126237 | 17 | 57860231 | 57891094 | 343727 | 31068 |
| ZFP90 | NC_010448.4 | 100620865 | 6 | 18312931 | 18335458 | -343936 | 12136 |
| STIP1 | NC_010444.4 | 100623923 | 2 | 7911443 | 7939000 | -345365 | 2765 |
| ORC3 | NC_010443.5 | 100519878 | 1 | 55982132 | 56044244 | -347759 | 456 |
| SIM1 | NC_010443.5 | 100154026 | 1 | 67213685 | 67294455 | 348845 | 532 |
| MIR144 | NC_010454.4 | 104796811 | 12 | 45088958 | 45089033 | 350573 | 21651 |
| NAV2 | NC_010444.4 | 100521067 | 2 | 39263939 | 40083539 | 350853 | 3261 |
| DNAH11 | NC_010451.4 | 100620543 | 9 | 90420044 | 90768327 | 352609 | 18969 |
| MNX1 | NC_010460.4 | 100511165 | 18 | 1724930 | 1730724 | 353607 | 31192 |
| DGKZ | NC_010444.4 | 100519533 | 2 | 16134046 | 16176043 | 360590 | 3107 |
| ABHD12 | NC_010459.5 | 100621636 | 17 | 30997477 | 31056307 | 360735 | 30584 |
| ZFR | NC_010458.4 | 100624470 | 16 | 18561909 | 18656341 | -361047 | 29800 |
| KDM5A | NC_010447.5 | 100294704 | 5 | 67628791 | 67716143 | -361261 | 11525 |
| NDUFAB1 | NC_010445.4 | 100312976 | 3 | 22619458 | 22631575 | -362582 | 6184 |
| COL18A1 | NC_010455.5 | 100624675 | 13 | 207907835 | 207984082 | -363332 | 25815 |
| MPO | NC_010454.4 | 100517120 | 12 | 34557031 | 34570091 | 365191 | 21455 |
| MYL9 | NC_010459.5 | 100157760 | 17 | 39788165 | 39795240 | -365426 | 30793 |
| HSDL2 | NC_010443.5 | 100155587 | 1 | 253124358 | 253191309 | -366014 | 2073 |
| ABL1 | NC_010443.5 | 100524544 | 1 | 270761641 | 270906708 | -367308 | 2372 |
| NISCH | NC_010455.5 | 100156417 | 13 | 34595788 | 34630306 | -367675 | 22974 |
| FERMT3 | NC_010444.4 | 100525326 | 2 | 7886781 | 7909892 | -372250 | 2764 |
| FLOT2 | NC_010454.4 | 100518752 | 12 | 45102205 | 45120503 | 372931 | 21652 |
| WDR48 | NC_010455.5 | 100518629 | 13 | 23735566 | 23786312 | -379086 | 22440 |
| PPIG | NC_010457.5 | 100155786 | 15 | 75940397 | 75979168 | 381178 | 28453 |
| FGF14 | NC_010453.5 | 100156844 | 11 | 70251374 | 70861941 | -382452 | 20542 |
| ERC1 | NC_010447.5 | 100513988 | 5 | 68223722 | 68608705 | 382486 | 11534 |
| TKT | NC_010455.5 | 100127150 | 13 | 35348921 | 35377518 | 382498 | 23010 |
| TAS2R38 | NC_010460.4 | 100624167 | 18 | 7982188 | 7983921 | 385494 | 31331 |
| SERPINE3 | NC_010453.5 | 100157886 | 11 | 16465521 | 16500580 | 385655 | 20252 |
| PRPF4 | NC_010443.5 | 100622501 | 1 | 253906258 | 253925683 | 392124 | 2090 |
| SH3BP2 | NC_010450.4 | 100626548 | 8 | 1583169 | 1619038 | 392979 | 16627 |
| TSPOAP1 | NC_010454.4 | 100514918 | 12 | 34578698 | 34606623 | 394291 | 21456 |
| VEGFB | NC_010444.4 | 100521837 | 2 | 7872873 | 7876149 | -396075 | 2760 |
| ANKFY1 | NC_010454.4 | 100522641 | 12 | 50167959 | 50251861 | -397134 | 21798 |
| SLBP | NC_010450.4 | 100513605 | 8 | 802940 | 818161 | -397575 | 16607 |
| ARFGAP2 | NC_010444.4 | 100513395 | 2 | 15390547 | 15401230 | -398567 | 3093 |
| SLC10A2 | NC_010453.5 | 100625109 | 11 | 71328801 | 71348779 | 399681 | 20560 |
| SEMA3G | NC_010455.5 | 100157653 | 13 | 34574014 | 34585686 | -400872 | 22970 |
| PCK1 | NC_010459.5 | 100144531 | 17 | 57930507 | 57936523 | 401579 | 31071 |
| GINS1 | NC_010459.5 | 106504085 | 17 | 31060540 | 31076807 | 402517 | 30585 |
| E2F6 | NC_010445.4 | 100737827 | 3 | 125248232 | 125271039 | 403793 | 8620 |
| CREB3L1 | NC_010444.4 | 100516663 | 2 | 16186206 | 16220948 | 409122 | 3108 |
| PACSIN3 | NC_010444.4 | 100512824 | 2 | 15380297 | 15389893 | -409360 | 3092 |
| MIR142 | NC_010454.4 | 100498767 | 12 | 34609378 | 34609457 | 411048 | 21457 |
| CLEC5A | NC_010460.4 | 397050 | 18 | 8004423 | 8014390 | 411846 | 31332 |
| PHF12 | NC_010454.4 | 100515341 | 12 | 45129864 | 45174755 | 413887 | 21655 |
| TRIML2 | NC_010459.5 | 100737283 | 17 | 7310656 | 7326072 | -416584 | 30393 |
| RARS2 | NC_010443.5 | 100518457 | 1 | 55906508 | 55982094 | -416646 | 455 |
| PLCB3 | NC_010444.4 | 100525148 | 2 | 7844931 | 7860183 | -418029 | 2757 |
| CCNC | NC_010443.5 | 100153639 | 1 | 66472622 | 66499754 | -419037 | 519 |
| PAPSS2 | NC_010456.5 | 100156262 | 14 | 99718299 | 99816346 | -419492 | 26996 |
| RNF183 | NC_010443.5 | 100622036 | 1 | 253924122 | 253966702 | 421565 | 2092 |
| APBB1IP | NC_010452.4 | 100514179 | 10 | 49125074 | 49242864 | -425990 | 19884 |
| SUPT4H1 | NC_010454.4 | 100515097 | 12 | 34622775 | 34629681 | 427858 | 21458 |
| CD93 | NC_010459.5 | 100155660 | 17 | 30233275 | 30240041 | -429499 | 30557 |
| BAP1 | NC_010455.5 | 100154798 | 13 | 34544570 | 34553538 | -431668 | 22967 |
| BAD | NC_010444.4 | 100521065 | 2 | 7831192 | 7842864 | -433558 | 2755 |
| DDB2 | NC_010444.4 | 100513197 | 2 | 15342614 | 15370623 | -437837 | 3091 |
| MSI2 | NC_010454.4 | 100737226 | 12 | 33537163 | 33974845 | -442366 | 21433 |
| DCP1A | NC_010455.5 | 100623102 | 13 | 35392592 | 35455639 | 443394 | 23013 |
| PLK1 | NC_010445.4 | 396953 | 3 | 22537814 | 22550901 | -443741 | 6179 |
| FAM209B | NC_010459.5 | 100134988 | 17 | 57087126 | 57088955 | -443896 | 31057 |
| THBD | NC_010459.5 | 100157642 | 17 | 30219870 | 30223499 | -444473 | 30556 |
| SYCP1 | NC_010446.5 | 100513924 | 4 | 105592354 | 105730413 | -448360 | 10086 |
| ZBP1 | NC_010459.5 | 100144524 | 17 | 57975982 | 57986117 | 449114 | 31072 |
| KCNK4 | NC_010444.4 | 100524785 | 2 | 7817062 | 7824390 | -449860 | 2753 |
| ANKS1B | NC_010447.5 | 100513089 | 5 | 84080505 | 85148742 | -453534 | 11719 |
| CDH3 | NC_010448.4 | 100187727 | 6 | 18190971 | 18236184 | -454553 | 12135 |
| USP54 | NC_010456.5 | 100518042 | 14 | 76307223 | 76439842 | 455335 | 26804 |
| ACP2 | NC_010444.4 | 100513002 | 2 | 15334015 | 15342531 | -456182 | 3090 |
| LYRM9 | NC_010454.4 | 100623359 | 12 | 44271407 | 44289687 | -457876 | 21613 |
| OLFML3 | NC_010446.5 | 100158055 | 4 | 106566978 | 106571086 | 459289 | 10100 |
| TINAG | NC_010449.5 | 100153159 | 7 | 26448612 | 26522993 | 460442 | 15232 |
| ADD1 | NC_010450.4 | 100517353 | 8 | 1627146 | 1710653 | 460775 | 16629 |
| TFAM | NC_010456.5 | 397279 | 14 | 92261713 | 92279731 | 461788 | 26967 |
| KLHL23 | NC_010457.5 | 100520221 | 15 | 76029145 | 76051670 | 461803 | 28459 |
| ESRRA | NC_010444.4 | 100322868 | 2 | 7803504 | 7812868 | -462400 | 2751 |
| ERN2 | NC_010445.4 | 100624656 | 3 | 22510067 | 22537872 | -464129 | 6176 |
| SGTB | NC_010458.4 | 100513246 | 16 | 44184536 | 44225896 | -464142 | 30036 |
| NR1H3 | NC_010444.4 | 397553 | 2 | 15315283 | 15336785 | -468421 | 3089 |
| PRDM12 | NC_010443.5 | 100522920 | 1 | 270723880 | 270739468 | -469808 | 2370 |
| PRDX5 | NC_010444.4 | 397273 | 2 | 7798672 | 7801955 | -470273 | 2749 |
| NNAT | NC_010459.5 | 449004 | 17 | 40625990 | 40629523 | 470629 | 30819 |
| MTMR12 | NC_010458.4 | 102167243 | 16 | 18461120 | 18537893 | -470666 | 29798 |
| SLC6A12 | NC_010447.5 | 100512716 | 5 | 67550285 | 67573858 | -471657 | 11523 |
| SP8 | NC_010451.4 | 100627196 | 9 | 89764671 | 89767428 | -475528 | 18958 |
| DNAH1 | NC_010455.5 | 100156016 | 13 | 34455154 | 34544147 | -481072 | 22965 |
| SLC35A1 | NC_010443.5 | 100517983 | 1 | 55859619 | 55895785 | -483245 | 454 |
| PIP | NC_010460.4 | 100621376 | 18 | 7110528 | 7117274 | -483660 | 31285 |
| PTBP3 | NC_010443.5 | 768100 | 1 | 252986716 | 253093059 | -483960 | 2072 |
| CERS6 | NC_010457.5 | 100126279 | 15 | 74927088 | 75261558 | -484282 | 28426 |
| LMBR1 | NC_010460.4 | 100737654 | 18 | 1795462 | 1934951 | 490987 | 31194 |
| SCN11A | NC_010455.5 | 100739142 | 13 | 23602378 | 23692281 | -492696 | 22438 |
| CCDC88B | NC_010444.4 | 100525503 | 2 | 7767924 | 7784625 | -494312 | 2747 |
| FABP6 | NC_010458.4 | 397423 | 16 | 63297594 | 63303675 | 494543 | 30194 |
| PMEPA1 | NC_010459.5 | 100144523 | 17 | 57999287 | 58054329 | 494872 | 31073 |
| ALAD | NC_010443.5 | 100621486 | 1 | 254015426 | 254027584 | 497658 | 2096 |
